# Reasons for missing evidence in rehabilitation meta-analyses: a cross-sectional meta-research study

**DOI:** 10.1186/s12874-023-02064-7

**Published:** 2023-10-21

**Authors:** Stefano Giuseppe Lazzarini, Marzia Stella Yousif, Silvia Bargeri, Greta Castellini, Silvia Gianola

**Affiliations:** 1 IRCCS Fondazione Don Carlo Gnocchi, Milan, Italy; 2 Department of Clinical Science and Translational Medicine, Faculty of Medicine and Surgery, University of Rome Tor Vergata, Rome, Italy; 3 Unit of Clinical Epidemiology, IRCCS Istituto Ortopedico Galeazzi, Milan, Italy

**Keywords:** Missing evidence, Rehabilitation, Core outcome sets, Protocol registration

## Abstract

**Background:**

Systematic reviews of randomized controlled trials are the best evidence for informing on intervention effectiveness. Their results, however, can be biased due to omitted evidence in the quantitative analyses. We aimed to assess the proportion of randomized controlled trials omitted from meta-analyses in the rehabilitation field and explore related reasons.

**Methods:**

This is a cross-sectional meta-research study. For each systematic review included in a published selected sample in the rehabilitation field, we identified an index meta-analysis on the primary outcome and the main comparison. We then looked at all the studies considered eligible for the chosen comparison in the systematic review and identified those trials that have been omitted (i.e., not included) from each index meta-analysis. Reasons for omission were collected based on an eight-reason classification. We used descriptive statistics to describe the proportion of omitted trials overall and according to each reason.

**Results:**

Starting from a cohort of 827 systematic reviews, 131 index meta-analyses comprising a total of 1761 eligible trials were selected. Only 16 index meta-analyses included all eligible studies while 15 omitted studies without providing references. From the remaining 100 index meta-analyses, 717 trials (40,7%) were omitted overall. Specific reasons for omission were: "unable to distinguish between selective reporting and inadequate planning" (39,3%, *N* = 282), "inadequate planning" (17%, *N* = 122), "justified to be not included" (15,1%, *N* = 108), "incomplete reporting" (8,4%, *N* = 60), "selective reporting" (3,3%, *N* = 24) and other situations (e.g., outcome present but no motivation for omission) (5,2%, *N* = 37). The 11,7% (*N* = 84) of omitted trials were not assessed due to non-English language or full text not available.

**Conclusions:**

Almost half of the eligible trials were omitted from their index meta-analyses. Better reporting, protocol registration, definition and adoption of core outcome sets are needed to prevent omission of evidence in systematic reviews.

**Supplementary Information:**

The online version contains supplementary material available at 10.1186/s12874-023-02064-7.

## Introduction

The best evidence to assess interventions is informed by systematic reviews (SRs) of randomized controlled trials (RCTs). In a SR, the clinical effectiveness and safety is evaluated by calculating the weighted pooled estimate for the interventions on a specific outcome through a meta-analysis. The quantitative effect estimates, however, can be biased when a meta-analysis fails to include all the published and unpublished studies on a specific topic regarding that specific outcome. Indeed, systematic reviews' validity may be compromised not only when they selectively include trials, outcomes and results but also when results of some eligible trials are unavailable for inclusion.

This problem has been known as “non-reporting bias”, which can occur in different ways in RCTs [1–3]. For instance, it comprises both "selective reporting", when results are selected based on the nature of results, and "incomplete reporting", when results are reported in a way that cannot be used in meta-analysis [2, 4]. An example might be found in the lack of reporting or selective reporting of harms in published clinical trials, which can give a false impression of safety leading to misinformation for clinical and policy decisions [5]. It has been supported that statistically significant results are more likely to be published or reported in a complete way than non-significant ones [6–8]: including only such results might overestimate the effects of an intervention or underestimate its undesirable effect, leading to the uptake of interventions that were actually ineffective or harmful. Similarly, incomplete reporting can prevent studies' outcome data to be included in meta-analyses, thus resulting in an analysis of a subset of data which is a biased representation of all recorded outcomes [9–11]. RCTs failing to plan and measure important outcomes may also be seen as a missed opportunity and a waste of research [10], impacting on the reliability of the meta-analysis.

Several studies investigated the issues of selective outcome reporting [1, 12–18], incomplete reporting [19–22] and waste of research due to lack of planning [23–27] in several biomedical fields, showing an under-recognised problem that affects the conclusions in a substantial proportion of systematic reviews.

In rehabilitation, the quality of reporting and conducting in RCTs is still suboptimal in various fields (e.g, orthopedics, rheumatology, neurology) [28–30], influencing the validity of the effect estimates of rehabilitation interventions. To the best of our knowledge, there has been no assessment of the impact of evidence omission in meta-analyses in this specific field.

### Objectives

Starting from a recent meta-research study including 827 SRs in the rehabilitation field [31], the primary aim of this cross-sectional meta-research study was to assess the proportion of RCTs omitted from the index meta-analysis of Cochrane (CSRs) and non-Cochrane systematic review (nCSRs) for outcome-related issues. Secondly, we aimed to compare this proportion among CSRs and nCSRs.

## Materials and methods

### Study design

This is a cross-sectional meta-research study [32, 33]. The protocol was registered on Open Science Framework (OSF) (https://osf.io/p25zy/). Since the reporting checklist for methods research studies is currently under development [34], we adapted items from the Preferred Reporting Items for Systematic Reviews and Meta-analyses (PRISMA) for reporting meta-research studies [35].

### Selection and characteristics of systematic reviews

We started from the sample of Gianola et al. [31], which collected 827 SRs published in 2020 in the rehabilitation field, encompassing different areas such as orthopedics, neurology, geriatrics. We selected SRs of interventions including RCTs only with at least one pairwise meta-analysis (of at least two RCTs). SRs should be previously registered with a protocol available from a repository (i.e., PROSPERO, OSF) or published in a peer-reviewed journal. Empty reviews (i.e., reviews with no included studies) [36], reviews with no meta-analysis, reviews with dose–response meta-analyses, network meta-analyses, and meta-analyses of individual participant data have been excluded.

General characteristics were extracted from the SRs sample: country of the corresponding author, number of studies included in the SR and in the index meta-analysis, source of funding, source of protocol registration, reporting of a list of excluded studies, and if the SR excluded studies because they do not report any outcome of interest. Furthermore, we explored whether the SRs searched for unpublished literature and if the SRs planned to assess, assessed or discussed non-reporting bias of an entire study (i.e., publication bias) or of a planned outcome within a study (i.e., selective reporting).

### Identification of index meta-analysis

To perform the assessment, we selected an index meta-analysis (IMA) from each SR as our unit of analysis. We identified as IMA the meta-analysis on the primary outcome of the main comparison, as defined by the SR’s authors. In case of multiple meta-analyses on the primary outcome, or unclear definition of primary outcomes and comparisons, we considered as IMA the first meta-analysis reported in the results section. For each IMA, we then collected the outcome and the number of RCTs included.

### Identification of the primary studies omitted from the index meta-analysis

We first identified all the RCTs considered eligible for the chosen comparison either from the list of the included studies of the SRs or, when available, from the list of excluded studies. Particularly, we looked at studies that have been excluded from the SRs because they do not report any outcome of interest, which, for example, may potentially be selectively not reported in the primary study. For this reason, we also retrieved and assessed the full texts and protocols of these trials, when available. We finally identified all RCTs omitted (i.e., not included) from the IMA.

**Fig. 1:**
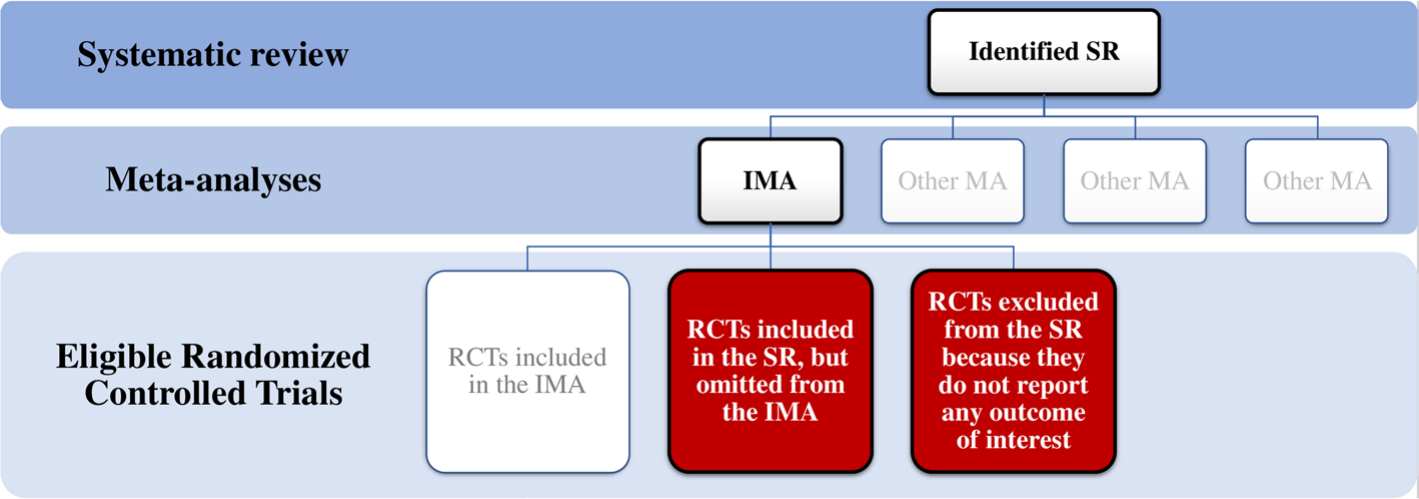
Process of IMA and omitted RCTs selection. Legend: *IMA* Index meta-analysis, *MA* Meta-analysis, *RCTs* Randomized controlled trials, *SR* Systematic review

The process of selection from the SR to the IMA and the omitted studies is represented in Fig. 1**.**

### Assessment of the reasons for omission

For each omitted RCT, we extracted the presence of a registered protocol, whether the trial was retrieved from the SR list of included or excluded studies and if the outcome identified by the IMA was planned and/or reported (either completely or incompletely) by each trial. To collect this information, we read each trial's full text and, if available, the registered protocol and/or statistical analysis plan, looking for discrepancies between them and between different sections of the published trial (i.e., abstract, methods, and results).

We then assessed the reason for omission, following the adapted classification of Yordanov et al. [10] (Table 1): a) *inadequate planning*; b) *selective reporting*; c) *incomplete reporting*; d) *unable to distinguish between selective reporting and inadequate planning*; e) *other situations*; f) *justified to be not included.* Non-English trials or trials with full text not available were not assessed, although still representing omitted trials.

**Table 1:** Reasons for omission, adapted from Yordanov et al. [10]

*Reason for omission*	*Explanation*
***Inadequate planning***	Whether the outcome was not planned according to the protocol nor reported in the trial reports
***Selective reporting***	Whether the outcome was planned according to the protocol, but not reported in the trial reports
***Incomplete reporting***	Whether the outcome was reported in the trial reports, but not in a way that allowed its inclusion in the meta-analysis (i.e., medians and interquartile ranges with no reference to the distribution of the data, data presented as means with no measure of variability, data presented graphically only, results presented just as p-values, Z or t values)
***Unable to distinguish between selective reporting and inadequate planning***	Whether no protocol or registry entry was available and the outcome was not reported in the reports
***Other situations***	The dichotomous outcome was listed in the trial reports, but there was no event
	The outcome concerned adverse events, but there was no event
	If it was judged that the outcome had been reported in a way that would allow it to be included in the meta-analysis, but the SR authors did not include it
***Justified to be not included***	In case the study could not be included in the meta-analysis for reasons related to the nature of the data (e.g., results reported as median and interquartile range as they were not normally distributed, or reported as mean change and standard deviation or 95% confidence interval) or to specific choices of the SR authors (e.g., the SR only included in the meta-analysis studies that had assessed an outcome with one or more selected outcome measures and the study in question assessed the same outcome with a different outcome measure)
***Not assessed—Language***	If the study was published in a language other than English
***Not assessed—Not found and not possible to judge***	In case the full text of the study was not retrieved

Legend: *SR* Systematic review

We adapted the category "*Inadequate planning*" of Yordanov et al. [10], considering when the omitted outcome had to be planned or it’s likely that the omitted outcome had to be planned.

For each omitted RCT we further extracted the publication year. Considering an uptake of one year for the 2013 SPIRIT (Standard Protocol Items: Recommendations for Interventional Trials) statement [37] to be disseminated in the scientific community, we decided to use 2014 as the cut-off year and to investigate *post-hoc* whether the publication year of the RCTs affected the classification of the omitted studies.

Before starting the assessment, a calibration phase was performed by two reviewers (SGL, MSY) piloting a small sample of 20 SRs (randomly selected and not equally distributed among CSRs and nCSRs). Disagreements were discussed with a third reviewer (SG). The final assessment was performed by one assessor. As a quality assessment measure, 30% of the sample was cross-checked by the second assessor and disagreements were discussed with a third reviewer (SG).

### Statistical analysis

We used descriptive statistics to assess the proportion of omitted RCTs overall (i.e., any reason for omission) and for each reason. We also assessed the proportion of omitted RCTs focusing only on those with registered protocol. Moreover, we assessed the proportion of IMAs with at least one omitted trial for each reason. We then descriptively compared these proportions in CSRs and nCSR.

## Results

### Selection and general characteristics of the included systematic reviews

Starting from a cohort of 827 SRs identified by Gianola et al. [31], 131 SRs and IMAs were selected for assessment (references in Appendix 1). The general characteristics of the included SRs are summarised in Supplementary Table 1 and reported in Appendix 2. 16,8% (*N* = 22) were CSR and 83,2% (*N* = 109) were nCSR.

Of the included SRs, 85,5% (*N *= 112) mentioned or assessed non-reporting biases (assessment of selective reporting in the Cochrane Risk of Bias Tool, assessment or planning of assessment of publication bias through visual assessment of funnel plots or appropriate statistical tests).

Seventy-seven SRs (58,8%) excluded studies because they do not report any outcome of interest (due to "no relevant outcome data" or similar reasons), and sixty of these (45,8% of the whole sample) did not report a list of excluded studies with detailed exclusion reasons for each study either (Supplementary Table 2).

### Characteristics of the index meta-analyses

Overall, the 131 IMAs included a median number of 6 (interquartile range [IQR] 3—10,5) and a total number of 1044 RCTs. Of these, 16 IMAs included all the eligible studies, whereas 15 IMAs reported the exclusion of RCTs because they do not report any outcome of interest but we were not able to assess them due to bibliographic references not reported. The remaining 100 IMAs omitted a total of 717 RCTs (median 3; IQR 1 – 7). Of these, 87,7% (*N* = 629) were retrieved from the list of included studies, while the remaining 12,3% (*N* = 88) were retrieved from the list of studies excluded because they do not report any outcome of interest. The characteristics of the IMAs are reported in Supplementary Table 3.

### Assessment of the reasons for omission

Overall, 717 out of 1761 total eligible RCTs (40,7%) have been omitted from the corresponding IMAs. The proportions of omitted RCTs for each of the reasons for omission are reported in Table 2.

**Table 2:** Proportion of omitted RCTs for each reason for omission

*Reason for omission*	Omitted RCTs		Corresponding IMAs^$^	
	N	%	N*	%
**Inadequate planning**	122	17%	40	40%
**Selective reporting**	24	3,3%	15	15%
**Incomplete reporting**	60	8,4%	38	38%
**Unable to distinguish between selective reporting and inadequate planning**	282	39,3%	68	68%
**Justified to be not included**	108	15,1%	43	43%
**Other situations**	37	5,2%	16	16%
**Not assessed – Language**	20	2,8%	8	8%
**Not assessed – Not found and not possible to judge**	64	8,9%	22	22%
**TOTAL**	717	100%	100	

^*^number of meta-analyses with at least one omitted study for each reason for omission; ^$^ The total number exceeds 100% because some meta-analyses omitted studies due to more than one reason

Legend: *IMAs* Index meta-analyses, *N* Number, *RCTs* Randomized controlled trials

The assessments of all primary studies, with their classification and rationale, are included in Appendix 3. The quality assessment on 30% of the assessed RCTs provided an almost perfect agreement between the two assessors (Cohen’s κ = 0,82).

Overall, only 29,3% (*N *= 210) of the assessed RCTs were registered (Supplementary Table 4). Table 3 reports the reasons for omission of registered RCTs only.

**Table 3:** Proportion of omitted registered RCTs for each reason for omission

*Reason for omission*	Omitted RCTs		Corresponding IMAs^$^	
	N	%	N*	%
**Inadequate planning**	122	58,1%	40	59,7%
**Selective reporting**	16	7,6%	11	16,4%
**Incomplete reporting**	22	10,5%	16	23,9%
**Unable to distinguish between selective reporting and inadequate planning**	0	0,0%	0	0,0%
**Justified to be not included**	40	19,0%	29	43,3%
**Other situations**	10	4,8%	6	9%
**Not assessed – Language**	0	0,0%	0	0,0%
**Not assessed – Not found and not possible to judge**	0	0,0%	0	0,0%
**TOTAL**	210	100%	67	

^*^number of meta-analyses with at least one omitted registered RCT for each reason for omission; ^$^ The total number exceeds 100% because some meta-analyses omitted studies due to more than one reason

Legend: *IMAs* Index meta-analyses, *N* Number, *RCTs* Randomized controlled trials

### Assessment of the reasons for omission according to the publication year 

According to the publication year cut-off, proportion of studies omitted from the IMA changes across reasons: those assessed as "*Unable to distinguish between selective reporting and inadequate planning*" considerably reduced, whereas those assessed as "*Inadequate planning*" more than doubled (Supplementary Table 5).

### Comparison between CSRs and nCSRs

**Fig. 2:**
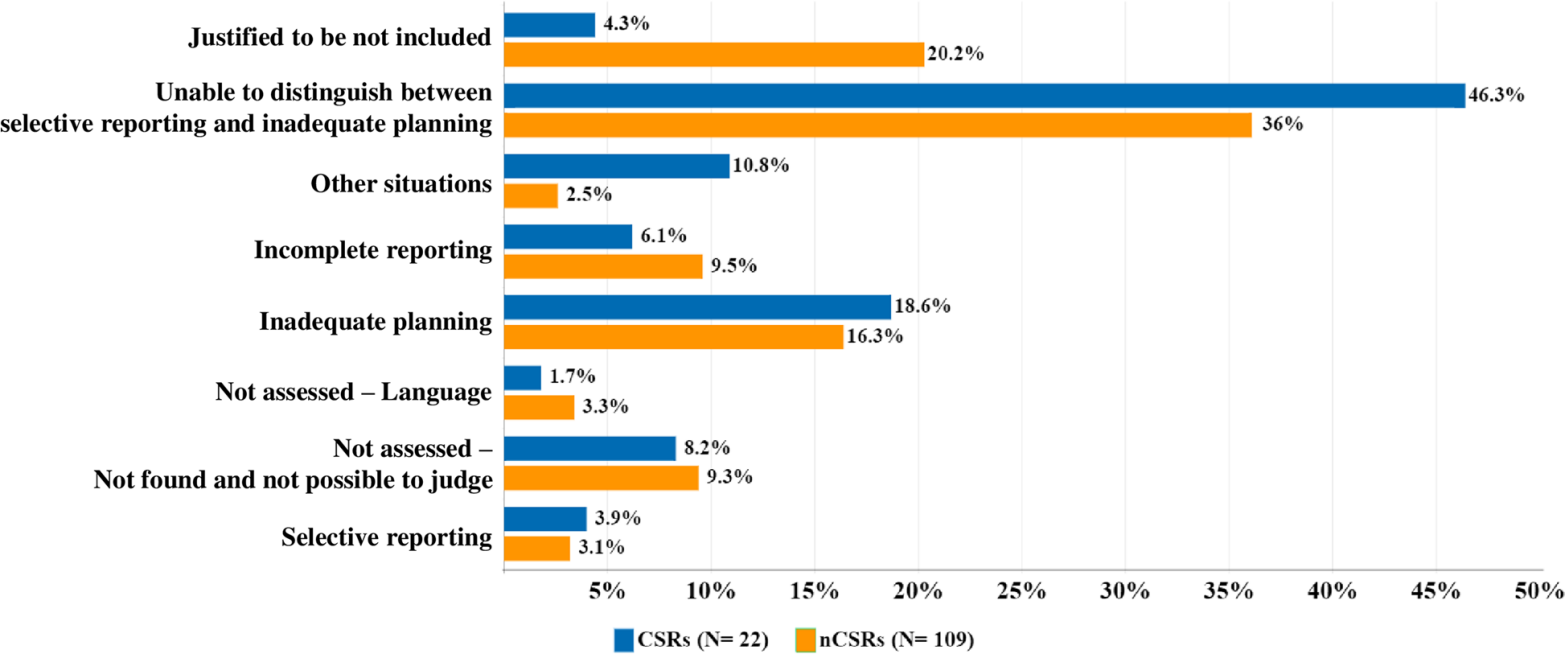
Comparison of the proportion of omitted RCTs for each reason for omission between CSRs and nCSRs. Abbreviations: *CSRs* Cochrane Systematic Reviews, *nCSRs* Non-Cochrane Systematic Reviews

Comparing CSRs and nCSRs, trial omission occurred in 59,2% (231 out of 390 eligible RCTs) and 35,4% (486 out of 1371 eligible RCTs) of the eligible studies, respectively (Supplementary Table 3). Figure 2 shows the comparison of the proportion of studies omitted for each reason between CSRs and nCSRs, in descending order. The reasons with greater differences are "Justified to be not included" (Δ% -15,9%), "Unable to distinguish between selective reporting and inadequate planning" (Δ% 10,3%) and "Other situations" (Δ% 8,3%).

## Discussion

Considering 131 SRs and corresponding IMAs, which included a total number of 1044 RCTs, our results show that omission of evidence occurred in more than 40% of eligible studies (40,7%; 717/1761) in 100 IMAs, comprising both studies already included in the SRs and studies retrieved from the list of excluded studies because they do not report any outcome of interest. Only 16 IMAs included all eligible studies, whereas 15 IMAs omitted studies excluded because they do not report any outcome of interest without providing the references.

At SRs level, almost 60% (77/131) of the selected SRs excluded studies because they do not report any outcome of interest. Furthermore, almost four of out five of these (60/77) did not provide a list of excluded studies either. These were in all cases nCRS as CSRs have a systematic ad hoc format for collecting and reporting the characteristics of the excluded studies [38]. This should be acknowledged as it prevented us to achieve a complete assessment and consequently the proportion of omitted trials has been underestimated.

At IMA level, some choices of SR authors prevented the possible inclusion of some trials that would have otherwise been included. For example, RCTs may have been omitted because they measured the outcome in a way that was different than what planned by the SR (e.g., different outcome measure than the one(s) identified by the SR, dichotomized outcome instead of continuous outcome or vice versa, different time frame than the one(s) selected by the SR).

At RCT level, 3,3% (24/717) and 8,4% (60/717) were omitted due to *selective reporting* and *incomplete reporting*, respectively. It is important to carefully evaluate the results of these meta-analyses, considering that certain RCTs may have been omitted due to the presumed negative or unfavourable nature of the results based on the magnitude or direction of the effect. If these excluded trials were included, they could potentially shift the effect estimate from positive to null or negative results. Considering registered RCTs only, the proportion of studies omitted due to *selective reporting* and *incomplete reporting* increases to 7,6% and 10,5%, respectively. To possibly overcome these issues, results of registered RCTs in the rehabilitation field should be made publicly available at ClinicalTrials.gov or at any other registry within one year as it already is for trials on drugs and devices [39], since it has been shown that results at ClinicalTrials.gov seem to be more completely reported than in published reports [40].

Almost 40% (39,3%, 282/717) of the omitted RCTs were classified as "*Unable to distinguish between selective reporting and inadequate planning*", not contributing to 68% (*N* = 68) of meta-analyses. This is a direct consequence of trial non-registration, which occurred in 59% (423/717) of omitted RCTs although it is common knowledge that *“studies involving human beings must be registered”* as stated in the Declaration of Helsinki [41]. Nevertheless, a great number of studies are still not previously registered. Since 2014, however, it should be acknowledged that this phenomenon has been improving, as shown in Supplementary Table 4.

Planning problems were observed in 17% (122/717) of the RCTs with a missing contribution in 40% (*N* = 40) of the meta-analyses. The creation and implementation of core outcome sets will help reduce research waste and judge when a study has truly failed in planning and measuring an important outcome [42, 43]. When considering registered RCTs only, planning issues represent the main reason for omission.

Studies that were legitimately omitted from the IMAs (i.e., *"Justified to be not included"*) accounted for 15,1% (108/717) of the omitted RCTs, including those reporting results in a different modality (i.e., mean change and standard deviations, repeated measures time x treatment) (2%, 14/717) and those that provided results as medians due to the non-normal distribution of the data (0,6%, 4/717). Additionally, other RCTs were legitimately omitted because used a different outcome measure than the one(s) selected by the SR authors (7,5%, 54/717), because measured outcome at a different time frame (2%, 14/717), because they were secondary analyses or follow-ups of studies included in the IMA or of studies omitted from the IMA (and consequently already assessed) (1,3%, 9/717) or for other reasons (1,8%, 13/717).

Comparing CSRs and nCSRs, the former present a higher proportion of omitted RCTs (59,2% vs. 35,4%, respectively). This may be a consequence of the fact that CSRs had more comprehensive searches (i.e., published sources and unpublished sources) [44], included sources with more often incomplete or hardly accessible data (i.e., congress abstracts, theses) and provided a list of excluded studies, being more methodologically rigorous and showing better reporting and higher quality [45, 46] compared to nCSRs. Conversely, the majority of nCSRs (94/109) did not provide a list of excluded studies, thus reducing the number of RCTs assessed from nCSRs and underestimating the proportion of omitted RCTs from these sources. Among RCTs omitted form CSRs and nCSRs, it seems that differences may exist, but they are probably related to the specific outcomes/comparisons addressed by the individual SRs rather than a real difference between CSRs and nCSRs.

### Comparison with previous studies

Our results slightly differ from those obtained by Yordanov et al. [10], who showed that, in a sample of CSRs of different medical fields, 78% of RCTs included did not contribute to meta-analyses of the most important outcomes showing a waste of research in a large part avoidable. Specifically, they reported a higher proportion of studies omitted due to inadequate planning and incomplete reporting. However, in Yordanov et al. a) only CRSs in different fields of medicine published between 2011 and 2014 were used to identify studies to be assessed, whereas we included both CSRs and nCSRs published in 2020 in the rehabilitation field; b) all the meta-analyses contributing to the Summary of Findings were evaluated, whereas we focused on IMAs only; c) only RCTs published after 2010 were assessed to maximise the possibility of identifying study registrations or protocols, whereas we considered RCTs irrespective of the publication year; d) studies excluded from the SRs because they do not report any outcome of interest were not assessed, whereas we assessed them. Furthermore, studies that were omitted because they reported data differently than what planned by the SR were classified as incomplete reporting by Yordanov et al., whereas in the present work an additional reason ("Justified") was added.

### Strengths and limitations

To the best of our knowledge, this is the first study in the rehabilitation field to focus on omitted studies from IMAs and investigate the reasons behind this. We assessed a high number of SRs, including both CSRs and nCSRs on several interventions and clinical conditions and more than 700 RCTs omitted from their IMAs.

The present study has some limitations: i) there might be studies that were improperly assessed or conversely unassessed that should have been assessed because of poor reporting of SRs on included and not included studies and lack of exclusion reason(s); ii) the identification of omitted RCTs was solely based on the studies included (and not) in each SR, but it was not possible to quantify omitted trials due to inaccuracy in the selection by the SRs; iii) we read the RCTs' protocols only when the reference and/or registration number were reported by the authors and we did not search registries or contact authors for clarification; iv) RCTs that were included in the meta-analysis were not assessed; v) SRs with meta-analysis of one study only, SRs with no meta-analysis and empty SRs were not included, but omission of trials might occur in these cases as well (particularly in excluded studies for the empty ones); vi) non-English studies have not been assessed. These considerations suggest that this phenomenon may have been underestimated in this work.

Finally, due to paucity of core outcome set available in literature and the wide range of health conditions addressed by our sample, we did not consider the core outcome sets for assessing the "*Inadequate planning*" omission reason but we limited our assessment to the lack of planning of the outcome in the registered protocol, when available.

### Clinical and research implications

From a clinical point of view, our results warn clinicians, consumers and policy makers about the reliability of the effect estimates provide by meta-analyses in the rehabilitation field irrespective of the quality of the reviews. Since missing results might systematically differ from the included ones [2], failing to include some RCTs may substantially bias the results of the meta-analyses. The impact of such missing results might be particularly serious when omission is caused by the nature of the results based on magnitude or direction of the effect.

From a research point of view, our results may serve as a call for researchers to improve reporting in RCTs, to register clinical trials on international reviewed registries, such as ClinicalTrials.gov and the WHO International Clinical Trials Registry, and to plan and measure outcomes that are relevant to the consumers and the public health [42]. Additionally, our work may also recommend systematic reviewers to systematically check RCTs protocols to identify whether the outcome was planned or likely to be measured, but not reported in the publication even in trials that do not report any outcome of interest in order to transparently justify the reasons for exclusion [47].

## Conclusion

Almost half of the eligible RCTs have been omitted from the index meta-analyses of CSRs and nCSRs for outcome-related reasons, representing a missed opportunity to include evidence in rehabilitation research. Compared to nCSRs, CSRs omitted a higher proportion of eligible studies. Our results highlight the urgent need for better reporting and implementation of core outcome sets for each clinical condition to be produced and used in the design of clinical studies in rehabilitation. As well, previous registration for every clinical study should be systematically performed.

### Supplementary Information

**Additional file 1: Appendix 1.** References of included systematic reviews.

**Additional file 2: Appendix 2.** Characteristics of included systematic reviews.

**Additional file 3: Appendix 3.** Assessment of the reason for omission.

**Additional file 4. **Prisma Checklist.

**Additional file 5: Supplementary Table 1. **Characteristics of included reviews.

**Additional file 6: Supplementary Table 2. **Absolute frequencies, relative frequencies and column percentages obtained by cross-referencing the information on the list of excluded studies with detailed exclusion reasons for each study and the exclusion of studies because they do not report any outcome of interest.

**Additional file 7: Supplementary Table 3. **Characteristics of the index meta–analyses.

**Additional file 8: Supplementary Table 4. **Information concerning a registered protocol.

**Additional file 9: Supplementary Table 5. **Comparison of the proportion of omitted RCTs for each reason for omission according to year of publication.

## Data Availability

All data generated or analysed during this study are included in this published article and stored at https://osf.io/p25zy/.

## Abbreviations

CSR: Cochrane systematic review
IMA: Index meta-analysis
IQR: Interquartile range
nCSR: Non-Cochrane systematic review
OSF: Open Science Framework
PRISMA: Preferred Reporting Items for Systematic Reviews and Meta-analyses
RCT: Randomized controlled trial
SPIRIT: Standard Protocol Items: Recommendations for Interventional Trials
SR: Systematic review
