# Supplementary material for: Reasons for missing evidence in rehabilitation meta-analyses: a cross-sectional meta-research study
Source: BMC Med Res Methodol. 2023 Oct 21;23:245. doi: 10.1186/s12874-023-02064-7 (PMC10590516; doi:10.1186/s12874-023-02064-7)
Supplement: Supplementary file 1 — Additional file 1: Appendix 1. References of included systematic reviews. [file 12874_2023_2064_MOESM1_ESM.docx]

**Appendix 1 – References of included systematic reviews**

1. Alghamdi SM, Barker RE, Alsulayyim ASS, Alasmari AM, Banya WAS, Polkey MI, et al. Use of oscillatory positive expiratory pressure (OPEP) devices to augment sputum clearance in COPD: a systematic review and meta-analysis. Thorax. 2020;75(10):855-63.

2. Almeida KAM, Rocha AP, Carvas N, Pinto A. Rehabilitation Interventions for Shoulder Dysfunction in Patients With Head and Neck Cancer: Systematic Review and Meta-Analysis. Phys Ther. 2020;100(11):1997-2008.

3. Amedoro A, Berardi A, Conte A, Pelosin E, Valente D, Maggi G, et al. The effect of aquatic physical therapy on patients with multiple sclerosis: A systematic review and meta-analysis. Mult Scler Relat Disord. 2020;41:102022.

4. Araújo PA, Starling JMP, Oliveira VC, Gontijo APB, Mancini MC. Combining balance-training interventions with other active interventions may enhance effects on postural control in children and adolescents with cerebral palsy: a systematic review and meta-analysis. Braz J Phys Ther. 2020;24(4):295-305.

5. Arora M, Harvey LA, Glinsky JV, Nier L, Lavrencic L, Kifley A, et al. Electrical stimulation for treating pressure ulcers. Cochrane Database Syst Rev. 2020;1(1):Cd012196.

6. Avendaño-Coy J, Comino-Suárez N, Grande-Muñoz J, Avendaño-López C, Gómez-Soriano J. Extracorporeal shockwave therapy improves pain and function in subjects with knee osteoarthritis: A systematic review and meta-analysis of randomized clinical trials. Int J Surg. 2020;82:64-75.

7. Azambuja ACM, de Oliveira LZ, Sbruzzi G. Inspiratory Muscle Training in Patients With Heart Failure: What Is New? Systematic Review and Meta-Analysis. Phys Ther. 2020;100(12):2099-109.

8. Barclay RE, Stevenson TJ, Poluha W, Semenko B, Schubert J. Mental practice for treating upper extremity deficits in individuals with hemiparesis after stroke. Cochrane Database Syst Rev. 2020;5(5):Cd005950.

9. Beckmann M, Bruun-Olsen V, Pripp AH, Bergland A, Smith T, Heiberg KE. Effect of exercise interventions in the early phase to improve physical function after hip fracture - A systematic review and meta-analysis. Physiotherapy. 2020;108:90-7.

10. Biazus-Sehn LF, Schuch FB, Firth J, Stigger FS. Effects of physical exercise on cognitive function of older adults with mild cognitive impairment: A systematic review and meta-analysis. Arch Gerontol Geriatr. 2020;89:104048.

11. Bjarnason-Wehrens B, Nebel R, Jensen K, Hackbusch M, Grilli M, Gielen S, et al. Exercise-based cardiac rehabilitation in patients with reduced left ventricular ejection fraction: The Cardiac Rehabilitation Outcome Study in Heart Failure (CROS-HF): A systematic review and meta-analysis. Eur J Prev Cardiol. 2020;27(9):929-52.

12. Bossen D, Broekema A, Visser B, Brons A, Timmerman A, van Etten-Jamaludin F, et al. Effectiveness of Serious Games to Increase Physical Activity in Children With a Chronic Disease: Systematic Review With Meta-Analysis. J Med Internet Res. 2020;22(4):e14549.

13. Bricca A, Harris LK, Jäger M, Smith SM, Juhl CB, Skou ST. Benefits and harms of exercise therapy in people with multimorbidity: A systematic review and meta-analysis of randomised controlled trials. Ageing Res Rev. 2020;63:101166.

14. Burge AT, Cox NS, Abramson MJ, Holland AE. Interventions for promoting physical activity in people with chronic obstructive pulmonary disease (COPD). Cochrane Database Syst Rev. 2020;4(4):Cd012626.

15. Cabanas-Valdés R, Serra-Llobet P, Rodriguez-Rubio PR, López-de-Celis C, Llauró-Fores M, Calvo-Sanz J. The effectiveness of extracorporeal shock wave therapy for improving upper limb spasticity and functionality in stroke patients: a systematic review and meta-analysis. Clin Rehabil. 2020;34(9):1141-56.

16. Candelaria D, Randall S, Ladak L, Gallagher R. Health-related quality of life and exercise-based cardiac rehabilitation in contemporary acute coronary syndrome patients: a systematic review and meta-analysis. Qual Life Res. 2020;29(3):579-92.

17. Carneiro L, Afonso J, Ramirez-Campillo R, Murawska-Ciałowciz E, Marques A, Clemente FM. The Effects of Exclusively Resistance Training-Based Supervised Programs in People with Depression: A Systematic Review and Meta-Analysis of Randomized Controlled Trials. Int J Environ Res Public Health. 2020;17(18).

18. Casey MB, Smart KM, Segurado R, Doody C. Multidisciplinary-based Rehabilitation (MBR) Compared With Active Physical Interventions for Pain and Disability in Adults With Chronic Pain: A Systematic Review and Meta-analysis. Clin J Pain. 2020;36(11):874-86.

19. Chae CS, Jun JH, Im S, Jang Y, Park GY. Effectiveness of Hydrotherapy on Balance and Paretic Knee Strength in Patients With Stroke: A Systematic Review and Meta-Analysis of Randomized Controlled Trials. Am J Phys Med Rehabil. 2020;99(5):409-19.

20. Chaovalit S, Taylor NF, Dodd KJ. Sit-to-stand exercise programs improve sit-to-stand performance in people with physical impairments due to health conditions: a systematic review and meta-analysis. Disabil Rehabil. 2020;42(9):1202-11.

21. Chen L, Ye L, Liu H, Yang P, Yang B. Extracorporeal Shock Wave Therapy for the Treatment of Osteoarthritis: A Systematic Review and Meta-Analysis. Biomed Res Int. 2020;2020:1907821.

22. Chiu HC, Ada L, Bania TA. Mechanically assisted walking training for walking, participation, and quality of life in children with cerebral palsy. Cochrane Database Syst Rev. 2020;11(11):Cd013114.

23. Chow G, Gan JKE, Chan JKY, Wu XV, Klainin-Yobas P. Effectiveness of psychosocial interventions among older adults with mild cognitive impairment: a systematic review and meta-analysis. Aging Ment Health. 2021;25(11):1986-97.

24. Corregidor-Sánchez AI, Segura-Fragoso A, Rodríguez-Hernández M, Jiménez-Rojas C, Polonio-López B, Criado-Álvarez JJ. Effectiveness of virtual reality technology on functional mobility of older adults: systematic review and meta-analysis. Age Ageing. 2021;50(2):370-9.

25. Dai W, Leng X, Wang J, Hu X, Ao Y. Rehabilitation regimen for non-surgical treatment of Achilles tendon rupture: A systematic review and meta-analysis of randomised controlled trials. J Sci Med Sport. 2021;24(6):536-43.

26. de Almeida SIL, Gomes da Silva M, Marques A. Home-Based Physical Activity Programs for People With Dementia: Systematic Review and Meta-Analysis. Gerontologist. 2020;60(8):600-8.

27. de Lima FF, Cavalheri V, Silva BSA, Grigoletto I, Uzeloto JS, Ramos D, et al. Elastic Resistance Training Produces Benefits Similar to Conventional Resistance Training in People With Chronic Obstructive Pulmonary Disease: Systematic Review and Meta-Analysis. Phys Ther. 2020;100(11):1891-905.

28. De Miguel-Rubio A, Rubio MD, Salazar A, Camacho R, Lucena-Anton D. Effectiveness of Virtual Reality on Functional Performance after Spinal Cord Injury: A Systematic Review and Meta-Analysis of Randomized Controlled Trials. J Clin Med. 2020;9(7).

29. de Oliveira Silva D, Pazzinatto MF, Rathleff MS, Holden S, Bell E, Azevedo F, et al. Patient Education for Patellofemoral Pain: A Systematic Review. J Orthop Sports Phys Ther. 2020;50(7):388-96.

30. Di Tella S, Pagliari C, Blasi V, Mendozzi L, Rovaris M, Baglio F. Integrated telerehabilitation approach in multiple sclerosis: A systematic review and meta-analysis. J Telemed Telecare. 2020;26(7-8):385-99.

31. Dobler CC, Morrow AS, Farah MH, Beuschel B, Majzoub AM, Wilson ME, et al. Nonpharmacologic Therapies in Patients With Exacerbation of Chronic Obstructive Pulmonary Disease: A Systematic Review With Meta-Analysis. Mayo Clin Proc. 2020;95(6):1169-83.

32. Dos Santos IK, Ashe MC, Cobucci RN, Soares GM, de Oliveira Maranhão TM, Dantas PMS. The effect of exercise as an intervention for women with polycystic ovary syndrome: A systematic review and meta-analysis. Medicine (Baltimore). 2020;99(16):e19644.

33. Duarte RV, Nevitt S, McNicol E, Taylor RS, Buchser E, North RB, et al. Systematic review and meta-analysis of placebo/sham controlled randomised trials of spinal cord stimulation for neuropathic pain. Pain. 2020;161(1):24-35.

34. Duncan S, McAuley DF, Walshe M, McGaughey J, Anand R, Fallis R, et al. Interventions for oropharyngeal dysphagia in acute and critical care: a systematic review and meta-analysis. Intensive Care Med. 2020;46(7):1326-38.

35. Ebadi S, Henschke N, Forogh B, Nakhostin Ansari N, van Tulder MW, Babaei-Ghazani A, et al. Therapeutic ultrasound for chronic low back pain. Cochrane Database Syst Rev. 2020;7(7):Cd009169.

36. Elsner B, Kugler J, Pohl M, Mehrholz J. Transcranial direct current stimulation (tDCS) for improving activities of daily living, and physical and cognitive functioning, in people after stroke. Cochrane Database Syst Rev. 2020;11(11):Cd009645.

37. Estévez-López F, Maestre-Cascales C, Russell D, Álvarez-Gallardo IC, Rodriguez-Ayllon M, Hughes CM, et al. Effectiveness of Exercise on Fatigue and Sleep Quality in Fibromyalgia: A Systematic Review and Meta-analysis of Randomized Trials. Arch Phys Med Rehabil. 2021;102(4):752-61.

38. Fandim JV, Saragiotto BT, Porfírio GJM, Santana RF. Effectiveness of virtual reality in children and young adults with cerebral palsy: a systematic review of randomized controlled trial. Braz J Phys Ther. 2021;25(4):369-86.

39. Farrell D, Artom M, Czuber-Dochan W, Jelsness-Jørgensen LP, Norton C, Savage E. Interventions for fatigue in inflammatory bowel disease. Cochrane Database Syst Rev. 2020;4(4):Cd012005.

40. Ferlito JV, Pecce SAP, Oselame L, De Marchi T. The blood flow restriction training effect in knee osteoarthritis people: a systematic review and meta-analysis. Clin Rehabil. 2020;34(11):1378-90.

41. Fernández López R, Antolí A. Computer-based cognitive interventions in acquired brain injury: A systematic review and meta-analysis of randomized controlled trials. PLoS One. 2020;15(7):e0235510.

42. Ferreira V, Carvas N, Jr., Artilheiro MC, Pompeu JE, Hassan SA, Kasawara KT. Interactive Video Gaming Improves Functional Balance in Poststroke Individuals: Meta-Analysis of Randomized Controlled Trials. Eval Health Prof. 2020;43(1):23-32.

43. Galeoto G, Polidori AM, Spallone M, Mollica R, Berardi A, Vanacore N, et al. Evaluation of physiotherapy and speech therapy treatment in patients with apraxia: a systematic review and meta-analysis. Clin Ter. 2020;171(5):e454-e65.

44. Galvão-Moreira LV, de Castro LO, Moura ECR, de Oliveira CMB, Nogueira Neto J, Gomes L, et al. Pool-based exercise for amelioration of pain in adults with fibromyalgia syndrome: A systematic review and meta-analysis. Mod Rheumatol. 2021;31(4):904-11.

45. Gamble K, Chiu A, Peiris C. Core Stability Exercises in Addition to Usual Care Physiotherapy Improve Stability and Balance After Stroke: A Systematic Review and Meta-analysis. Arch Phys Med Rehabil. 2021;102(4):762-75.

46. García-Muñoz C, Cortés-Vega MD, Heredia-Rizo AM, Martín-Valero R, García-Bernal MI, Casuso-Holgado MJ. Effectiveness of Vestibular Training for Balance and Dizziness Rehabilitation in People with Multiple Sclerosis: A Systematic Review and Meta-Analysis. J Clin Med. 2020;9(2).

47. Gates NJ, Rutjes AW, Di Nisio M, Karim S, Chong LY, March E, et al. Computerised cognitive training for 12 or more weeks for maintaining cognitive function in cognitively healthy people in late life. Cochrane Database Syst Rev. 2020;2(2):Cd012277.

48. Gianola S, Castellini G, Pecoraro V, Monticone M, Banfi G, Moja L. Effect of Muscular Exercise on Patients With Muscular Dystrophy: A Systematic Review and Meta-Analysis of the Literature. Front Neurol. 2020;11:958.

49. Grønfeldt BM, Lindberg Nielsen J, Mieritz RM, Lund H, Aagaard P. Effect of blood-flow restricted vs heavy-load strength training on muscle strength: Systematic review and meta-analysis. Scand J Med Sci Sports. 2020;30(5):837-48.

50. Gutiérrez-Espinoza H, Araya-Quintanilla F, Cereceda-Muriel C, Álvarez-Bueno C, Martínez-Vizcaíno V, Cavero-Redondo I. Effect of supervised physiotherapy versus home exercise program in patients with subacromial impingement syndrome: A systematic review and meta-analysis. Phys Ther Sport. 2020;41:34-42.

51. Hall LM, Neumann P, Hodges PW. Do features of randomized controlled trials of pelvic floor muscle training for postprostatectomy urinary incontinence differentiate successful from unsuccessful patient outcomes? A systematic review with a series of meta-analyses. Neurourol Urodyn. 2020;39(2):533-46.

52. Han CY, Miller M, Yaxley A, Baldwin C, Woodman R, Sharma Y. Effectiveness of combined exercise and nutrition interventions in prefrail or frail older hospitalised patients: a systematic review and meta-analysis. BMJ Open. 2020;10(12):e040146.

53. He Y, Li K, Chen Q, Yin J, Bai D. Repetitive Transcranial Magnetic Stimulation on Motor Recovery for Patients With Stroke: A PRISMA Compliant Systematic Review and Meta-analysis. Am J Phys Med Rehabil. 2020;99(2):99-108.

54. Hislop AC, Collins NJ, Tucker K, Deasy M, Semciw AI. Does adding hip exercises to quadriceps exercises result in superior outcomes in pain, function and quality of life for people with knee osteoarthritis? A systematic review and meta-analysis. Br J Sports Med. 2020;54(5):263-71.

55. Hopewell S, Copsey B, Nicolson P, Adedire B, Boniface G, Lamb S. Multifactorial interventions for preventing falls in older people living in the community: a systematic review and meta-analysis of 41 trials and almost 20 000 participants. Br J Sports Med. 2020;54(22):1340-50.

56. Huang Q, Yan P, Xiong H, Shuai T, Liu J, Zhu L, et al. Extracorporeal Shock Wave Therapy for Treating Foot Ulcers in Adults With Type 1 and Type 2 Diabetes: A Systematic Review and Meta-Analysis of Randomized Controlled Trials. Can J Diabetes. 2020;44(2):196-204.e3.

57. Husted RS, Juhl C, Troelsen A, Thorborg K, Kallemose T, Rathleff MS, et al. The relationship between prescribed pre-operative knee-extensor exercise dosage and effect on knee-extensor strength prior to and following total knee arthroplasty: a systematic review and meta-regression analysis of randomized controlled trials. Osteoarthritis Cartilage. 2020;28(11):1412-26.

58. Imamura S, Inagaki T, Terada J, Nagashima K, Katsura H, Tatsumi K. Long-term efficacy of pulmonary rehabilitation with home-based or low frequent maintenance programs in patients with chronic obstructive pulmonary disease: a meta-analysis. Ann Palliat Med. 2020;9(5):2606-15.

59. Jansen SC, Abaraogu UO, Lauret GJ, Fakhry F, Fokkenrood HJ, Teijink JA. Modes of exercise training for intermittent claudication. Cochrane Database Syst Rev. 2020;8(8):Cd009638.

60. Jaqueline da Cunha M, Rech KD, Salazar AP, Pagnussat AS. Functional electrical stimulation of the peroneal nerve improves post-stroke gait speed when combined with physiotherapy. A systematic review and meta-analysis. Ann Phys Rehabil Med. 2021;64(1):101388.

61. Kamonseki DH, Lopes EP, van der Meer HA, Calixtre LB. Effectiveness of manual therapy in patients with tension-type headache. A systematic review and meta-analysis. Disabil Rehabil. 2022;44(10):1780-9.

62. Khattab S, Wiley E, Fang H, Richardson J, MacDermid J, Tang A. The effects of exercise on cognition post-stroke: are there sex differences? A systematic review and meta-analysis. Disabil Rehabil. 2021;43(25):3574-91.

63. Kim Y, Vakula MN, Waller B, Bressel E. A systematic review and meta-analysis comparing the effect of aquatic and land exercise on dynamic balance in older adults. BMC Geriatr. 2020;20(1):302.

64. Klil-Drori S, Klil-Drori AJ, Pira S, Rej S. Exercise Intervention for Late-Life Depression: A Meta-Analysis. J Clin Psychiatry. 2020;81(1).

65. Laver KE, Adey-Wakeling Z, Crotty M, Lannin NA, George S, Sherrington C. Telerehabilitation services for stroke. Cochrane Database Syst Rev. 2020;1(1):Cd010255.

66. Lee KH, Lee JY, Kim B. Person-Centered Care in Persons Living With Dementia: A Systematic Review and Meta-analysis. Gerontologist. 2022;62(4):e253-e64.

67. Li J, Zhu W, Gao X, Li X. Comparison of Arthroscopic Partial Meniscectomy to Physical Therapy following Degenerative Meniscus Tears: A Systematic Review and Meta-analysis. Biomed Res Int. 2020;2020:1709415.

68. Liao CD, Chen HC, Kuo YC, Tsauo JY, Huang SW, Liou TH. Effects of Muscle Strength Training on Muscle Mass Gain and Hypertrophy in Older Adults With Osteoarthritis: A Systematic Review and Meta-Analysis. Arthritis Care Res (Hoboken). 2020;72(12):1703-18.

69. Luo L, Meng H, Wang Z, Zhu S, Yuan S, Wang Y, et al. Effect of high-intensity exercise on cardiorespiratory fitness in stroke survivors: A systematic review and meta-analysis. Ann Phys Rehabil Med. 2020;63(1):59-68.

70. Maginador G, Lixandrão ME, Bortolozo HI, Vechin FC, Sarian LO, Derchain S, et al. Aerobic Exercise-Induced Changes in Cardiorespiratory Fitness in Breast Cancer Patients Receiving Chemotherapy: A Systematic Review and Meta-Analysis. Cancers (Basel). 2020;12(8).

71. Martinez-Calderon J, Flores-Cortes M, Morales-Asencio JM, Fernandez-Sanchez M, Luque-Suarez A. Which Interventions Enhance Pain Self-efficacy in People With Chronic Musculoskeletal Pain? A Systematic Review With Meta-analysis of Randomized Controlled Trials, Including Over 12 000 Participants. J Orthop Sports Phys Ther. 2020;50(8):418-30.

72. Mateo S, Di Marco J, Cucherat M, Gueyffier F, Rode G. Inconclusive efficacy of intervention on upper-limb function after tetraplegia: A systematic review and meta-analysis. Ann Phys Rehabil Med. 2020;63(3):230-40.

73. McGregor G, Powell R, Kimani P, Underwood M. Does contemporary exercise-based cardiac rehabilitation improve quality of life for people with coronary artery disease? A systematic review and meta-analysis. BMJ Open. 2020;10(6):e036089.

74. Mehrholz J, Thomas S, Kugler J, Pohl M, Elsner B. Electromechanical-assisted training for walking after stroke. Cochrane Database Syst Rev. 2020;10(10):Cd006185.

75. Mendes LA, Lima IN, Souza T, do Nascimento GC, Resqueti VR, Fregonezi GA. Motor neuroprosthesis for promoting recovery of function after stroke. Cochrane Database Syst Rev. 2020;1(1):Cd012991.

76. Mendonça LM, Leite HR, Zwerver J, Henschke N, Branco G, Oliveira VC. How strong is the evidence that conservative treatment reduces pain and improves function in individuals with patellar tendinopathy? A systematic review of randomised controlled trials including GRADE recommendations. Br J Sports Med. 2020;54(2):87-93.

77. Mihai EE, Dumitru L, Mihai IV, Berteanu M. Long-Term Efficacy of Extracorporeal Shock Wave Therapy on Lower Limb Post-Stroke Spasticity: A Systematic Review and Meta-Analysis of Randomized Controlled Trials. J Clin Med. 2020;10(1).

78. Moisset X, Pereira B, Ciampi de Andrade D, Fontaine D, Lantéri-Minet M, Mawet J. Neuromodulation techniques for acute and preventive migraine treatment: a systematic review and meta-analysis of randomized controlled trials. J Headache Pain. 2020;21(1):142.

79. Morishita S, Hamaue Y, Fukushima T, Tanaka T, Fu JB, Nakano J. Effect of Exercise on Mortality and Recurrence in Patients With Cancer: A Systematic Review and Meta-Analysis. Integr Cancer Ther. 2020;19:1534735420917462.

80. Moucheboeuf G, Griffier R, Gasq D, Glize B, Bouyer L, Dehail P, et al. Effects of robotic gait training after stroke: A meta-analysis. Ann Phys Rehabil Med. 2020;63(6):518-34.

81. Muñoz-Vigueras N, Prados-Román E, Valenza MC, Granados-Santiago M, Cabrera-Martos I, Rodríguez-Torres J, et al. Speech and language therapy treatment on hypokinetic dysarthria in Parkinson disease: Systematic review and meta-analysis. Clin Rehabil. 2021;35(5):639-55.

82. Nascimento LR, da Silva LA, Araújo Barcellos JVM, Teixeira-Salmela LF. Ankle-foot orthoses and continuous functional electrical stimulation improve walking speed after stroke: a systematic review and meta-analyses of randomized controlled trials. Physiotherapy. 2020;109:43-53.

83. Nascimento LR, Flores LC, de Menezes KKP, Teixeira-Salmela LF. Water-based exercises for improving walking speed, balance, and strength after stroke: a systematic review with meta-analyses of randomized trials. Physiotherapy. 2020;107:100-10.

84. Naunton J, Street G, Littlewood C, Haines T, Malliaras P. Effectiveness of progressive and resisted and non-progressive or non-resisted exercise in rotator cuff related shoulder pain: a systematic review and meta-analysis of randomized controlled trials. Clin Rehabil. 2020;34(9):1198-216.

85. Navarro-Santana MJ, Gómez-Chiguano GF, Somkereki MD, Fernández-de-Las-Peñas C, Cleland JA, Plaza-Manzano G. Effects of joint mobilisation on clinical manifestations of sympathetic nervous system activity: a systematic review and meta-analysis. Physiotherapy. 2020;107:118-32.

86. Nayak P, Mahmood A, Natarajan M, Hombali A, Prashanth CG, Solomon JM. Effect of aquatic therapy on balance and gait in stroke survivors: A systematic review and meta-analysis. Complement Ther Clin Pract. 2020;39:101110.

87. Oliveira VH, Mendonça KM, Monteiro KS, Silva IS, Santino TA, Nogueira PAM. Physical therapies for postural abnormalities in people with cystic fibrosis. Cochrane Database Syst Rev. 2020;3(3):Cd013018.

88. Orgeta V, McDonald KR, Poliakoff E, Hindle JV, Clare L, Leroi I. Cognitive training interventions for dementia and mild cognitive impairment in Parkinson's disease. Cochrane Database Syst Rev. 2020;2(2):Cd011961.

89. Pan H, Zhang P, Zhang Z, Yang Q. Arthroscopic partial meniscectomy combined with medical exercise therapy versus isolated medical exercise therapy for degenerative meniscal tear: A meta-analysis of randomized controlled trials. Int J Surg. 2020;79:222-32.

90. Paravlic AH, Tod D, Milanovic Z. Mental Simulation Practice Has Beneficial Effects on Patients' Physical Function Following Lower Limb Arthroplasty: A Systematic Review and Meta-analysis. Arch Phys Med Rehabil. 2020;101(8):1447-61.

91. Parmenter BJ, Mavros Y, Ritti Dias R, King S, Fiatarone Singh M. Resistance training as a treatment for older persons with peripheral artery disease: a systematic review and meta-analysis. Br J Sports Med. 2020;54(8):452-61.

92. Pazzianotto-Forti EM, Moreno MA, Plater E, Baruki SBS, Rasera-Junior I, Reid WD. Impact of Physical Training Programs on Physical Fitness in People With Class II and III Obesity: A Systematic Review and Meta-Analysis. Phys Ther. 2020;100(6):963-78.

93. Plaza-Manzano G, Gómez-Chiguano GF, Cleland JA, Arías-Buría JL, Fernández-de-Las-Peñas C, Navarro-Santana MJ. Effectiveness of percutaneous electrical nerve stimulation for musculoskeletal pain: A systematic review and meta-analysis. Eur J Pain. 2020;24(6):1023-44.

94. Pogrebnoy D, Dennett A. Exercise Programs Delivered According to Guidelines Improve Mobility in People With Stroke: A Systematic Review and Meta-analysis. Arch Phys Med Rehabil. 2020;101(1):154-65.

95. Pozuelo-Carrascosa DP, Carmona-Torres JM, Laredo-Aguilera JA, Latorre-Román P, Párraga-Montilla JA, Cobo-Cuenca AI. Effectiveness of Respiratory Muscle Training for Pulmonary Function and Walking Ability in Patients with Stroke: A Systematic Review with Meta-Analysis. Int J Environ Res Public Health. 2020;17(15).

96. Prosperini L, Tomassini V, Castelli L, Tacchino A, Brichetto G, Cattaneo D, et al. Exergames for balance dysfunction in neurological disability: a meta-analysis with meta-regression. J Neurol. 2021;268(9):3223-37.

97. Riberholt CG, Wagner V, Lindschou J, Gluud C, Mehlsen J, Møller K. Early head-up mobilisation versus standard care for patients with severe acquired brain injury: A systematic review with meta-analysis and Trial Sequential Analysis. PLoS One. 2020;15(8):e0237136.

98. Roberts KE, Rickett K, Feng S, Vagenas D, Woodward NE. Exercise therapies for preventing or treating aromatase inhibitor-induced musculoskeletal symptoms in early breast cancer. Cochrane Database Syst Rev. 2020;1(1):Cd012988.

99. Robson EK, Hodder RK, Kamper SJ, O'Brien KM, Williams A, Lee H, et al. Effectiveness of Weight-Loss Interventions for Reducing Pain and Disability in People With Common Musculoskeletal Disorders: A Systematic Review With Meta-Analysis. J Orthop Sports Phys Ther. 2020;50(6):319-33.

100. Rueda JR, Mugueta-Aguinaga I, Vilaró J, Rueda-Etxebarria M. Myofunctional therapy (oropharyngeal exercises) for obstructive sleep apnoea. Cochrane Database Syst Rev. 2020;11(11):Cd013449.

101. Sabe M, Kaiser S, Sentissi O. Physical exercise for negative symptoms of schizophrenia: Systematic review of randomized controlled trials and meta-analysis. Gen Hosp Psychiatry. 2020;62:13-20.

102. Saunders DH, Sanderson M, Hayes S, Johnson L, Kramer S, Carter DD, et al. Physical fitness training for stroke patients. Cochrane Database Syst Rev. 2020;3(3):Cd003316.

103. Shahabi S, Shabaninejad H, Kamali M, Jalali M, Ahmadi Teymourlouy A. The effects of ankle-foot orthoses on walking speed in patients with stroke: a systematic review and meta-analysis of randomized controlled trials. Clin Rehabil. 2020;34(2):145-59.

104. Silva S, Borges LR, Santiago L, Lucena L, Lindquist AR, Ribeiro T. Motor imagery for gait rehabilitation after stroke. Cochrane Database Syst Rev. 2020;9(9):Cd013019.

105. Singh B, Hayes SC, Spence RR, Steele ML, Millet GY, Gergele L. Exercise and colorectal cancer: a systematic review and meta-analysis of exercise safety, feasibility and effectiveness. Int J Behav Nutr Phys Act. 2020;17(1):122.

106. Skelly AC, Chou R, Dettori JR, Turner JA, Friedly JL, Rundell SD, et al. AHRQ Comparative Effectiveness Reviews. Noninvasive Nonpharmacological Treatment for Chronic Pain: A Systematic Review Update. Rockville (MD): Agency for Healthcare Research and Quality (US); 2020.

107. Smith TO, Gilbert AW, Sreekanta A, Sahota O, Griffin XL, Cross JL, et al. Enhanced rehabilitation and care models for adults with dementia following hip fracture surgery. Cochrane Database Syst Rev. 2020;2(2):Cd010569.

108. Su JJ, Yu DSF, Paguio JT. Effect of eHealth cardiac rehabilitation on health outcomes of coronary heart disease patients: A systematic review and meta-analysis. J Adv Nurs. 2020;76(3):754-72.

109. Surace SJ, Deitch J, Johnston RV, Buchbinder R. Shock wave therapy for rotator cuff disease with or without calcification. Cochrane Database Syst Rev. 2020;3(3):Cd008962.

110. Takahashi K, Momosaki R, Yasufuku Y, Nakamura N, Maeda K. Nutritional Therapy in Older Patients With Hip Fractures Undergoing Rehabilitation: A Systematic Review and Meta-Analysis. J Am Med Dir Assoc. 2020;21(9):1364-.e6.

111. Tomazoni SS, Almeida MO, Bjordal JM, Stausholm MB, Machado C, Leal-Junior ECP, et al. Photobiomodulation therapy does not decrease pain and disability in people with non-specific low back pain: a systematic review. J Physiother. 2020;66(3):155-65.

112. van Nispen RM, Virgili G, Hoeben M, Langelaan M, Klevering J, Keunen JE, et al. Low vision rehabilitation for better quality of life in visually impaired adults. Cochrane Database Syst Rev. 2020;1(1):Cd006543.

113. Waldauf P, Jiroutková K, Krajčová A, Puthucheary Z, Duška F. Effects of Rehabilitation Interventions on Clinical Outcomes in Critically Ill Patients: Systematic Review and Meta-Analysis of Randomized Controlled Trials. Crit Care Med. 2020;48(7):1055-65.

114. Wang J, Ren D, Liu Y, Wang Y, Zhang B, Xiao Q. Effects of early mobilization on the prognosis of critically ill patients: A systematic review and meta-analysis. Int J Nurs Stud. 2020;110:103708.

115. Wilhelm MP, Donaldson M, Griswold D, Learman KE, Garcia AN, Learman SM, et al. The Effects of Exercise Dosage on Neck-Related Pain and Disability: A Systematic Review With Meta-analysis. J Orthop Sports Phys Ther. 2020;50(11):607-21.

116. Woodley SJ, Lawrenson P, Boyle R, Cody JD, Mørkved S, Kernohan A, et al. Pelvic floor muscle training for preventing and treating urinary and faecal incontinence in antenatal and postnatal women. Cochrane Database Syst Rev. 2020;5(5):Cd007471.

117. Xie Q, Chen X, Xiao J, Liu S, Yang L, Chen J, et al. Acupuncture combined with speech rehabilitation training for post-stroke dysarthria: A systematic review and meta-analysis of randomized controlled trials. Integr Med Res. 2020;9(4):100431.

118. Xu C, Su X, Ma S, Shu Y, Zhang Y, Hu Y, et al. Effects of Exercise Training in Postoperative Patients With Congenital Heart Disease: A Systematic Review and Meta-Analysis of Randomized Controlled Trials. J Am Heart Assoc. 2020;9(5):e013516.

119. Yan RB, Zhang XL, Li YH, Hou JM, Chen H, Liu HL. Effect of transcranial direct-current stimulation on cognitive function in stroke patients: A systematic review and meta-analysis. PLoS One. 2020;15(6):e0233903.

120. Yang X, He H, Ye W, Perry TA, He C. Effects of Pulsed Electromagnetic Field Therapy on Pain, Stiffness, Physical Function, and Quality of Life in Patients With Osteoarthritis: A Systematic Review and Meta-Analysis of Randomized Placebo-Controlled Trials. Phys Ther. 2020;100(7):1118-31.

121. Yau DKW, Underwood MJ, Joynt GM, Lee A. Effect of preparative rehabilitation on recovery after cardiac surgery: A systematic review. Ann Phys Rehabil Med. 2021;64(2):101391.

122. Ye LF, Wang SM, Wang LH. Efficacy and Safety of Exercise Rehabilitation for Heart Failure Patients With Cardiac Resynchronization Therapy: A Systematic Review and Meta-Analysis. Front Physiol. 2020;11:980.

123. Yeh SW, Lin LF, Tam KW, Tsai CP, Hong CH, Kuan YC. Efficacy of robot-assisted gait training in multiple sclerosis: A systematic review and meta-analysis. Mult Scler Relat Disord. 2020;41:102034.

124. Yoo JI, Oh MK, Chun SW, Lee SU, Lee CH. The effect of focused extracorporeal shock wave therapy on myofascial pain syndrome of trapezius: A systematic review and meta-analysis. Medicine (Baltimore). 2020;99(7):e19085.

125. Yu B, Qiu H, Li J, Zhong C, Li J. Noninvasive Brain Stimulation Does Not Improve Neuropathic Pain in Individuals With Spinal Cord Injury: Evidence From a Meta-Analysis of 11 Randomized Controlled Trials. Am J Phys Med Rehabil. 2020;99(9):811-20.

126. Yue W, Han X, Luo J, Zeng Z, Yang M. Effect of music therapy on preterm infants in neonatal intensive care unit: Systematic review and meta-analysis of randomized controlled trials. J Adv Nurs. 2021;77(2):635-52.

127. Zhao H, Xie Y, Wang J, Li X, Li J. Pulmonary Rehabilitation Can Improve the Functional Capacity and Quality of Life for Pneumoconiosis Patients: A Systematic Review and Meta-Analysis. Biomed Res Int. 2020;2020:6174936.

128. Zhao Q, Dong C, Liu Z, Li M, Wang J, Yin Y, et al. The effectiveness of aquatic physical therapy intervention on disease activity and function of ankylosing spondylitis patients: a meta-analysis. Psychol Health Med. 2020;25(7):832-43.

129. Zhou XL, Wang LN, Wang J, Zhou L, Shen XH. Effects of exercise interventions for specific cognitive domains in old adults with mild cognitive impairment: A meta-analysis and subgroup analysis of randomized controlled trials. Medicine (Baltimore). 2020;99(31):e20105.

130. Zhu F, Zhang M, Wang D, Hong Q, Zeng C, Chen W. Yoga compared to non-exercise or physical therapy exercise on pain, disability, and quality of life for patients with chronic low back pain: A systematic review and meta-analysis of randomized controlled trials. PLoS One. 2020;15(9):e0238544.

131. Ziebart C, Bobos P, Furtado R, MacDermid JC, Bryant D, Szekeres M, et al. The Efficacy of Fall Hazards Identification on Fall Outcomes: A Systematic Review With Meta-analysis. Arch Rehabil Res Clin Transl. 2020;2(3):100065.
