# Supplementary material for: Reasons for missing evidence in rehabilitation meta-analyses: a cross-sectional meta-research study
Source: BMC Med Res Methodol. 2023 Oct 21;23:245. doi: 10.1186/s12874-023-02064-7 (PMC10590516; doi:10.1186/s12874-023-02064-7)
Supplement: Supplementary file 2 — Additional file 2: Appendix 2. Characteristics of included systematic reviews. [file 12874_2023_2064_MOESM2_ESM.docx]

**Appendix 2 – Characteristics of included systematic reviews**

***Legend***: 1= Frist author; 2= Country of the corresponding authors; 3= Journal; 4= Sources of funding; 5= Number of included studies; 6= Authors referred to a protocol? (PROSPERO registration/link in a repository (es. OSF), or published); 7= Grey literature or unpublished source?; 8= Reason for exclusion reported and available for detailed individual assessment; 9= Studies excluded because they do not report any outcome of interest; 10= Outcomes examined; 11= Outcome reporting bias mentioned; 12= Primary meta-analysis - Outcome to be assessed; 13= Protocol reporting primary meta-analysis outcome in general among outcomes; 14= Protocol reporting primary meta-analysis outcome among primary outcomes; 15= Primary meta-analysis - N included studies

| 1 | 2 | 3 | 4 | 5 | 6 | 7 | 8 | 9 | 10 | 11 | 12 | 13 | 14 | 15 |
| --- | --- | --- | --- | --- | --- | --- | --- | --- | --- | --- | --- | --- | --- | --- |
| Alghamdi | England | Thorax | No Profit | 8 | PROSPERO CRD42016041835 | N | N | N | Systematic review reporting "all reported primary and secondary outcomes of COPD were extracted", while the protocol reports "Changes in the sputum production of patients" as main outcome and "Frequency of exacerbations, Length of stay and Patient reported measures of sputum clearance ability" as additional outcomes. | Y | HRQoL measures (CAT and SGRQ) | N | N | 2 |
| Almeida | Brazil | Physical therapy | None | 7 | PROSPERO CRD42017080500 | Y | N | N | Not specified, protocol reporting "Any instrument that can measure shoulder muscle strength, function, range of motion, pain, tilt and misalignment will be included" | Y | Disfunction | N | N | 3 |
| Amedoro | Italy | Multiple sclerosis and related disorders | None | 11 | PROSPERO CRD42019143149 | N | N | N | Not specified, protocol reporting "The primary outcomes are the improvement of neurological aspects related to the MS, in particular fatigue, motor deficiencies, muscle strength or tone alterations, postural balance. The secondary outcome concerns depression and quality of life." | N | Modified fatigue impact scale (MFIS) Physical | Y | Y | 3 |
| Araújo | Brazil | Brazilian journal of physical therapy | No Profit | 7 | PROSPERO CRD42016043272 | N | N | Y | Postural stability | Y | Postural stability | Y | Y | 7 |
| Arora | Australia | The Cochrane database of systematic reviews | No Profit | 20 | Protocol (doi: 10.1002/14651858.CD012196) | Y | Y | N | Proportion of pressure ulcers healed, composite measures of pressure ulcers that captured different aspects of severity, surface area of pressure ulcers, time to complete healing, complications/adverse events, rate of pressure ulcer healing expressed as percentage rate of healing per week, quality of life, depression, consumers' perception of treatment effectiveness | Y | Proportion of pressure ulcers healed | Y | Y | 11 |
| Avendaño-Coy | Spain | International journal of surgery (London, England) | None | 14 | PROSPERO CRD42019126507 | Y | N | N | Subjective perception of pain, the Western Ontario and McMaster Universities Osteoarthritis Index (WOMAC), adverse effects, range of motion, functional tests, such as walking or climbing stairs. | N | Pain | Y | Y | 13 |
| Azambuja | Brazil | Physical therapy | No Profit | 14 | PROSPERO CRD42017080339 | N | N | N | Respiratory muscle strength, pulmonary function, functional capacity (assessed by distance walked in the 6-Minute Walk Test [6MWT] and maximum oxygen consumption [Vo2peak]), quality of life (assessed with the Minnesota Living With Heart Failure Questionnaire), and dyspnea | Y | Maximal inspiratory pressure (MIP) | Y | Y | 9 |
| Barclay | Canada | The Cochrane database of systematic reviews | None | 25 | Previous review (doi: 10.1002/14651858.CD005950.pub4) | Y | Y | N | Upper extremity activity, upper extremity impairment, activities of daily living, health-related quality of life, indicators of the economic costs of interventions, any measurement of adverse effects | Y | Upper extremity activity | Y | Y | 15 |
| Beckmann | Norway | Physiotherapy | No Profit | 9 | PROSPERO CRD42018091135 | N | N | Y | Physical function assessed using walking ability, walking speed, balance, muscle strength, mobility, and endurance. Pain, quality of life. | N | Physical function | Y | Y | 9 |
| Biazus-Sehn | Brazil | Archives of gerontology and geriatrics | No Profit | 27 | PROSPERO CRD42016037182 | N | N | Y | Global cognitive function or any specific domains of cognition, including cognitive speed, verbal fluency, immediate recall, delayed recall, working memory, executive function or attention | Y | Global Cognitive Function | N | N | 16 |
| Bjarnason-Wehrens | Germany | European journal of preventive cardiology | No Profit | 25 | PROSPERO CRD42017054833 | Y | N | Y | 1: All-cause mortality 2: Cardiovascular mortality; Hospitalization of any course; Hospitalization due to worsening heart failure; Combined endpoint of mortality and hospitalization of any cause; Cardiopulmonary exercise capacity (peak oxygen uptake (VO2peak)); Quality of life (Short-Form-36-Questionnaire [SF36], Minnesota Living With Heart Failure Questionnaire or other validated questionnaires). | Y | Time to event all-cause-mortality | Y | Y | 19 |
| Bossen | Netherlands | Journal of medical Internet research | No Profit | 9 | PROSPERO CRD42018070662 | N | N | N | Level of physical activity, cardiorespiratory endurance, muscular strength, body composition, and quality of life | Y | Level of physical activity [moderate to vigorous physical activity (minutes per day)] | Y | Y | 7 |
| Bricca | Denmark | Ageing research reviews | No Profit | 23 | PROSPERO CRD42020150628 | Y | N | Y | Health-related quality of life (HRQoL), physical function, depression, anxiety and adverse events | Y | Health-related quality of life | Y | Y | 13 |
| Burge | Australia | The Cochrane database of systematic reviews | None | 76 | Protocol (doi: 10.1002/14651858.CD012626) | Y | Y | Y | Participation in physical activity or sedentary behaviour, Health-related quality of life, Exercise capacity, Adverse events, Adherence to intervention | Y | Physical activity: change in step count (steps per day) | Y | Y | 3 |
| Cabanas-Valdés | Spain | Clinical rehabilitation | None | 24 | PROSPERO CRD42018099194 | N | N | N/A | Upper limb spasticity and functionality | Y | Spasticity | Y | Y | 2 |
| Candelaria | Australia | Quality of life research | Not Reported | 14 | PROSPERO CRD42018109816 | Y | N | Y | HRQoL | Y | HRQoL- SF36 domain of physical functioning | Y | Y | 5 |
| Carneiro | Portugal | International journal of environmental research and public health | None | 4 | International Platform of Registered Systematic Review and Meta-analysis Protocols (INPLASY202070004) | N | Y | N | Mean change in depressive symptoms; Physical (e.g., performance tests, body composition, perceived exertion); (ii) psychosocial (e.g., body image and appearance, reporting of positive or negative feelings, self-esteem, cognitive evaluations, memory and concentration tasks) | Y | Depressive symptoms | Y | Y | 4 |
| Casey | Ireland | The Clinical journal of pain | No Profit | 31 | PROSPERO CRD42018117824 | Y | N | Y | Pain and disability | Y | Pain | Y | Y | 19 |
| Chae | South Korea | American journal of physical medicine & rehabilitation | Not Reported | 11 | PROSPERO CRD42019131894 | N | N | Y | Balance and strength | Y | Balance [Berg Balance Scale] | Y | Y | 10 |
| Chaovalit | Australia | Disability and rehabilitation | Not Reported | 8 | PROSPERO CRD42017067780 | N | Y | N | Any patient related outcomes: Impairments (e.g., strength, spasticity, balance, weight bearing) Activities (e.g., gait, sit to stand, functional reach) Participation (e.g., home, sporting, work, school, social) | Y | Sit-to-stand performance | Y | Y | 4 |
| Chen | China | BioMed research international | No Profit | 32 | PROSPERO CRD42019120534 | N | N | Y | Pain and function | Y | Pain | Y | Y | 11 |
| Chiu | Taiwan | The Cochrane database of systematic reviews | None | 17 | Protocol (doi: 10.1002/14651858.CD013114) | Y | Y | N | Mobility, Gross motor function, Adverse events, participation, Quality of life | Y | Gross motor function | Y | Y | 2 |
| Chow | Czech Republic | Aging & mental health | Mixed | 43 | PROSPERO CRD42019132255 | Y | N | Y | Cognitive function, including memory and executive function (primary outcome), depression and ADL (secondary outcomes) | Y | Memory | Y | Y | 5 |
| Corregidor-Sánchez | Spain | Age and ageing | None | 18 | PROSPERO CRD42019131630 | N | Y | N | Activities of daily living, Functional mobility, Falls risk in elderly people | Y | Functional mobility | Y | Y | 3 |
| Dai | China | Journal of science and medicine in sport | No Profit | 8 | PROSPERO CRD42020218986 | N | N | Y | The primary outcome measure was re-rupture rate after nonoperative treatment. Secondary outcome measures included complication rate, functional outcome scores, quality-of-life out- comes, return to sporting activity rate, return to work rate, resource use after nonoperative treatment. | Y | Re-rupture rate | Y | Y | 8 |
| de Almeida | Portugal | The Gerontologist | No Profit | 16 | PROSPERO CRD42017059951 | N | N | N | Not pre-specified, protocol reporting "All measures of or related physical activity or exercise intervention will be considered" | N | Cognitive function, measured with Mini-Mental Status Examination (MMSE) | N | N | 3 |
| de Lima | Brazil | Physical therapy | No Profit | 8 | PROSPERO CRD42018091731 | N | N | Y | Muscle strength, Functional exercise capacity, Quality of life, Dyspnea | N | Muscle Strength | Y | Y | 3 |
| De Miguel-Rubio | Spain | Journal of clinical medicine | None | 7 | PROSPERO CRD42018093855 | N | N | Y | Functional performance | Y | Functional performance [FIM scale] | Y | Y | 3 |
| de Oliveira Silva | Australia | The Journal of orthopaedic and sports physical therapy | Not Reported | 8 | PROSPERO CRD42018088671 | N | N | Y | Self-reported pain, self-reported function, objective function, quality of life, lower-limb strength, and psychological factors (ie, depression, anxiety). | Y | Self-reported pain | Y | Y | 3 |
| Di Tella | Italy | Journal of telemedicine and telecare | No Profit | 9 | PROSPERO CRD42018096682 | Y | N | N | Motor outcomes comprised motor impairment (balance and mobility) and patient-reported motor disability. Cognitive outcomes comprised measures of: executive functions; processing speed; verbal fluency; visual memory; and working memory. Participation outcomes comprised: fatigue; everyday functioning; quality of life; selfefficacy; and depression | Y | Motor outcomes | Y | Y | 4 |
| Dobler | Australia | Mayo Clinic proceedings | No Profit | 30 | PROSPERO CRD42018111609 | Y | N | Y | Repeat exacerbations; dyspnea; mortality; quality of life; hospital readmission; ICU admission; functional capacity (ie, timed walking tests and endurance tests); need for intubation; symptoms; lung function; and adverse events (AEs). | Y | Dyspnea ( numerica scales, VAS, Borg) at end of intervention | Y | Y | 3 |
| Dos Santos | Brazil | Medicine | No Profit | 10 | PROSPERO CRD42017058869 | N | N | Y | Menstrual cycle, hormonal levels, ovulation rate (reproductive function); and minor outcomes: metabolic parameters (HOMA-IR), and body composition (weight (kg), body mass index (BMI), waist circumference (cm), and waist hip ratio. | Y | Reproductive function (hormones) | Y | Y | 6 |
| Duarte | United States | Pain | Mixed | 8 | PROSPERO CRD42018090412 | N | Y | N | Pain | Y | Pain | Y | Y | 6 |
| Duncan | England | Intensive care medicine | No Profit | 22 | PROSPERO CRD42018116849 | Y | N | Y | Time taken to return to oral intake; Change in incidence of aspiration and change in severity of secretions and pharyngeal residue; Adverse events associated with the intervention; Incidence of pneumonia; Economic and resource cost; Quality of life; Nutritional status | Y | Aspiration incidence post-intervention | Y | Y | 4 |
| Ebadi | Iran | The Cochrane database of systematic reviews | No Profit | 10 | Previous review (doi: 10.1002/14651858.CD009169.pub2) | Y | Y | N | Symptoms (e.g. pain), back-specific functional status, overall improvement or satisfaction with treatment, well-being, disability, lumbar range of motion, muscle strength, endurance. | Y | Pain [VAS] | Y | Y | 8 |
| Elsner | Germany | The Cochrane database of systematic reviews | No Profit | 67 | Previous review (doi: 10.1002/14651858.CD009645.pub4) | Y | Y | Y | Activities of daily living, upper limb function, lower limb function, muscle strength, cognitive abilities (including spatial neglect), safety | Y | Activities of daily living | Y | Y | 19 |
| Estévez-López | Spain | Archives of physical medicine and rehabilitation | No Profit | 41 | PROSPERO CRD42018118005 | N | N | N/A | Fatigue and sleep quality | N | Fatigue | Y | Y | 17 |
| Fandim | Brazil | Brazilian journal of physical therapy | None | 38 | PROSPERO CRD42018102759 | Y | N | N/A | Upper limb function, lower limb function (divided into gait and strength of the lower limbs), and postural control and balance; global motor function, spatial functions, perception and cognition, motivation, motor learning, and adverse events | Y | Upper limb function | Y | Y | 11 |
| Farrell | Ireland | The Cochrane database of systematic reviews | No Profit | 14 | Protocol (doi: 10.1002/14651858.CD012005) | Y | Y | Y | Fatigue, loss of energy, vigour and vitality, quality of life, adverse events. | N | Fatigue | Y | Y | 2 |
| Ferlito | Brazil | Clinical rehabilitation | None | 5 | PROSPERO CRD42020137000 | N | N | Y | Measure of strength (maximum voluntary force production) and mass muscle, pain/discomfort during and after exercise, Knee’s functionality of and quality of life | N | Knee muscle strength | Y | Y | 3 |
| Fernández López | Spain | PloS one | No Profit | 8 | PROSPERO CRD42019138833 | Y | N | Y | Evaluation of cognition using standardized tests | Y | Verbal working memory | N | N | 6 |
| Ferreira | Canada | Evaluation & the health professions | None | 11 | PROSPERO CRD42015024685 | N | N | Y | Functional balance and mobility | N | Functional balance | Y | Y | 6 |
| Galeoto | Italy | La Clinica terapeutica | Not Reported | 5 | PROSPERO CRD42017065429 | N | N | N | Apraxic limb functions, difficulty in carrying out transitive and intransitive gestures, level of independence in carrying out ADLs, articulation’s accuracy and buccofacial performing movements | Y | Ideomotor Apraxia’s (IMA) test | Y | Y | 2 |
| Galvão-Moreira | Brazil | Modern rheumatology | No Profit | 14 | PROSPERO CRD42019136755 | N | Y | Y | Not clearly specified in the selection criteria, protocol reporting "reduction in pain scores" AND "reduction of fibromyalgia impact in patients well-being and functionality". | N | Pain [VAS score] | Y | Y | 10 |
| Gamble | Australia | Archives of physical medicine and rehabilitation | Not Reported | 12 | PROSPERO CRD42017077834 | N | N | N | "Primary outcomes of interest were all patient health outcomes that align with the World Health Organization’s International Classification of Functioning, Disability, and Health framework. The outcomes were classified under the following headings: body function, activity, and participation. Based on these classifications, we considered measures of lung function, trunk control, and static balance to be measures of body function, and measures of complex balance activities were considered to represent activity. Secondary outcomes were adverse events." | Y | Trunk control | Y | Y | 8 |
| García-Muñoz | Spain | Journal of clinical medicine | None | 7 | PROSPERO CRD42019134230 | N | N | N/A | The primary outcome measures were balance and dizziness. Secondary outcomes were fatigue, walking speed and depression. | N | postural control | Y | Y | 2 |
| Gates | Netherlands | The Cochrane database of systematic reviews | No Profit | 8 | Previous review (doi: 10.1002/14651858.CD012277.pub2) | Y | Y | Y | Global cognitive functioning, episodic memory, speed of processing, executive function, attention/working memory, verbal fluency, quality of life/psychological well-being, daily function, adverse events | Y | Global cognitive function | Y | Y | 2 |
| Gianola | Italy | Frontiers in neurology | No Profit | 13 | PROSPERO CRD42019127456 | Y | Y | Y | Muscle strength, endurance during walking, motor abilities, fatigue, adverse events | Y | Muscle strength | Y | Y | 9 |
| Grønfeldt | Denmark | Scandinavian journal of medicine & science in sports | Not Reported | 16 | PROSPERO CRD42014013382 | N | N | Y | Maximal muscle strength | Y | Maximal muscle strength | Y | Y | 16 |
| Gutiérrez-Espinoza | Chile | Physical therapy in sport : official journal of the Association of Chartered Physiotherapists in Sports Medicine | None | 4 | PROSPERO CRD42018086348 | N | N | N | Shoulder or upper extremity function, pain intensity and active or passive range of motion | Y | Shoulder function | Y | Y | 4 |
| Hall | Australia | Neurourology and urodynamics | No Profit | 22 | PROSPERO CRD42017059589 | N | N | Y | Number of men incontinent at 3 months | Y | Number of men incontinent at 3 months | Y | Y | 5 |
| Han | Australia | BMJ open | None | 11 | PROSPERO CRD42020153934 | N | N | Y | At least one of the frailty indicators: nutritional status, physical function, cognitive function and mood, physical activity, mobility, energy, psychology or frailty biomarkers. Health related quality of life (HRQOL), patient and caregiver anxiety and depression, caregiver burden, adverse events (including falls, delirium, medical complications, delayed hospital discharge and emergency department) and cost-effectiveness/health resource utilisation. | N | Reduction in frailty | Y | Y | 3 |
| He | China | American journal of physical medicine & rehabilitation | No Profit | 20 | PROSPERO CRD42017058219 | N | N | Y | Motor function, grip strength, upper limb motor function, activities of daily living, stroke severity, safety | Y | Motor Function | Y | Y | 15 |
| Hislop | Australia | British journal of sports medicine | None | 8 | PROSPERO CRD42017057857 | N | N | N | Pain, self-reported physical function, physical function tests or QoL | Y | Pain | Y | Y | 5 |
| Hopewell | England | British journal of sports medicine | Profit | 41 | PROSPERO CRD42018102549 | Y | N | Y | Our primary outcome was the rate of falls (ie, number of falls per person-years). Secondary outcomes included the risk of: sustaining one or more falls; recurrent falls (defined as two or more falls in a specified time period); one or more fall-related fractures; a fall that required hospital admission; and a fall that required medical attention (eg, attended hospital emergency department, required general practitioner (GP) consultation) and health-related quality of life (measured using validated scale, eg, EQ-5D or similar). | Y | Rate of falls (≥12 months’ follow-up) | Y | Y | 20 |
| Huang | China | Canadian journal of diabetes | No Profit | 8 | PROSPERO CRD42018118096 | N | N | N/A | Not clearly stated, protocol reports "wound surface area, percentage of re-epithelialization, number of complete healing, number of unchanged ulcers, blood flow perfusion rates, average wound healing time and ulcer-related pain" | Y | Wound surface area | Y | Y | 2 |
| Husted | Denmark | Osteoarthritis and cartilage | No Profit | 12 | PROSPERO CRD42018076308 | N | N | Y | Knee-extensor strength, knee pain, patient reported physical function (e.g., activities of daily living), knee-related performance-based function (e.g., ability to climb stairs) and adverse events. | Y | Knee-extensor strength | Y | Y | 12 |
| Imamura | Japan | Annals of palliative medicine | No Profit | 7 | PROSPERO CRD42019109718 | N | N | Y | Exercise capacity and HRQOL | Y | Exercise capacity [6MWD] | Y | Y | 5 |
| Jansen | Netherlands | The Cochrane database of systematic reviews | No Profit | 10 | Previous review (doi: 10.1002/14651858.CD009638.pub2) | Y | Y | Y | Maximal treadmill walking distance or time, Pain-free treadmill walking distance or time, Health-related quality of life scores, Self-reported functional impairment | Y | Maximum walking distance | Y | Y | 6 |
| Jaqueline da Cunha | Brazil | Annals of physical and rehabilitation medicine | No Profit | 14 | PROSPERO CRD42019127552 | Y | N | Y | Gait speed, active ankle dorsiflexion mobility, balance and functional mobility. | Y | Gait speed | Y | Y | 12 |
| Kamonseki | Brazil | Disability and rehabilitation | No Profit | 15 | PROSPERO CRD42019114214 | N | N | Y | Pain intensity, pain frequency and impact of headache | Y | Pain intensity | Y | Y | 8 |
| Khattab | Canada | Disability and rehabilitation | None | 17 | PROSPERO CRD42018092757 | N | N | Y | Attention and processing speed, memory, executive functioning, visuospatial ability, language, and global cognition | Y | Memory (episodic memory and overall memory) | Y | Y | 7 |
| Kim | United States | BMC geriatrics | None | 11 | Protocol details can be accessed via (https://osf.io/9bc4y) | N | N | Y | Trial and protocol state "at least one outcome related to dynamic balance" | Y | Dynamic steady-state balance | Y | Y | 3 |
| Klil-Drori | Canada | The Journal of clinical psychiatry | None | 9 | PROSPERO CRD42018103292 | N | N | Y | Depressive symptoms assessed by validated measure: MADRS, PHQ-9, SCID, GDS-15, GDS-30, or any other validated scale | Y | Depressive symptoms | Y | Y | 9 |
| Laver | Australia | The Cochrane database of systematic reviews | Not Reported | 22 | Previous review (doi: 10.1002/14651858.CD010255.pub2) | Y | Y | N | Activities of daily living, self-care and domestic life, mobility, balance, satisfaction, self-reported health-related quality of life, depression, upper limb function, cognitive function, functional communication, cost-effectiveness, adverse events | Y | Activities of daily living | Y | Y | 2 |
| Lee | South Korea | The Gerontologist | No Profit | 36 | PROSPERO CRD42020169376 | N | N | Y | Outcomes including depression, cognition, agitation, and neuropsychiatric symptoms | N | Depression | Y | N | 20 |
| Li | China | BioMed research international | No Profit | 6 | PROSPERO CRD42019125653 | Y | N | N/A | Functional outcome, pain and adverse events | Y | Function | Y | Y | 6 |
| Liao | Taiwan | Arthritis care & research | No Profit | 19 | PROSPERO CRD42019125118 | Y | N | Y | Muscle mass outcomes (lean body mass, lower extremity lean mass, and fat-free mass) and muscle volume outcomes (any measure of muscle morphology, such as muscle CSA, myofiber CSA, muscle thickness, and muscle circumference) | Y | Muscle mass | Y | Y | 4 |
| Luo | China | Annals of physical and rehabilitation medicine | No Profit | 31 | PROSPERO CRD42019130311 | Y | N | Y | The primary outcome measures were changes in the peak/ maximum level of oxygen uptake (VO2peak/VO2max), 6MWT, and fastest 10MWT. Secondary outcome measures were any adverse events (e.g. falls, pain, injuries). | Y | VO2 peak | Y | Y | 15 |
| Maginador | Brazil | Cancers | None | 9 | PROSPERO CRD42019134584 | N | N | Y | Maximal Volume of Oxygen Uptake (VO2max) | Y | Maximal Volume of Oxygen Uptake (VO2max) | Y | Y | 9 |
| Martinez-Calderon | Spain | The Journal of orthopaedic and sports physical therapy | No Profit | 60 | PROSPERO CRD42018117361 | Y | N | Y | Pain self-efficacy | Y | Pain self-efficacy | Y | Y | 4 |
| Mateo | France | Annals of physical and rehabilitation medicine | No Profit | 29 | PROSPERO CRD42018098506 | N | N | Y | Hand-arm function, upper-limb strength, and functional independence | Y | Hand-arm function | Y | N | 4 |
| McGregor | England | BMJ open | None | 24 | PROSPERO CRD42018110197 | N | N | Y | Quality of Life | Y | Quality of life [36-Item Short Form Survey Instrument (SF-36) physical function domain] | Y | Y | 5 |
| Mehrholz | England | The Cochrane database of systematic reviews | No Profit | 62 | Previous review (doi: 10.1002/14651858.CD006185.pub5) | Y | Y | N | Ability to walk independently, walking speed, walking capacity, adverse outcomes, withdrawal from the study for any reason | Y | Ability to walk independently | Y | Y | 18 |
| Mendes | Brazil | The Cochrane database of systematic reviews | No Profit | 4 | Protocol (doi: 10.1002/14651858.CD012991) | Y | Y | Y | 1: Independence in ADL (FIM), Barthel Index (BI), Motor Assessment Scale (MAS), Activities involving limbs 2: Participation scales of HRQoL, Exercise capacity, Balance, Adverse events | Y | Activities involving limbs | Y | Y | 2 |
| Mendonça | Brazil | British journal of sports medicine | None | 9 | PROSPERO CRD42017072082 | N | N | N | Pain and function | Y | Pain | Y | Y | 2 |
| Mihai | Romania | Journal of clinical medicine | None | 7 | PROSPERO CRD42020207093 | N | N | N | The primary outcome was spasticity grade assessed mainly while using the MAS. Other scales, such as Modified Modified Ashworth Scale (MMAS) and MTS, were also used for spasticity grade assessment. Secondary outcomes were passive range of motion (PROM), pain intensity, gait assessment, electrophysiological parameters, and adverse events related to the ESWT application. | N | Spasticity | Y | Y | 7 |
| Moisset | France | The journal of headache and pain | None | 38 | PROSPERO CRD42020181494 | N | N | Y | Number of headache days/month for preventive treatment, proportion of pain-free patients 2 h after treatment for acute treatment; quality of life, need for rescue medication, and Patient Global Impression of Change score | Y | Proportion of pain-free patients 2 h after treatment | Y | Y | 2 |
| Morishita | Japan | Integrative cancer therapies | No Profit | 8 | PROSPERO CRD42019140268 | N | N | Y | Mortality and recurrence | Y | Mortality | Y | Y | 8 |
| Moucheboeuf | France | Annals of physical and rehabilitation medicine | Not Reported | 33 | PROSPERO CRD42018092227 | Y | N | N/A | Gait speed, gait endurance, Berg Balance Scale (BBS), Functional Ambulation Classification (FAC) and Timed Up and Go scores. | Y | Gait speed | Y | Y | 11 |
| Muñoz-Vigueras | Spain | Clinical rehabilitation | None | 15 | PROSPERO CRD4201911 9605 | N | N | N | Not clearly reported, the protocol states "Sound pressure level (sustained phonation, reading, monologue), Forced vital capacity, Parkinson disease questionnaire" among the outcomes. | Y | Vocal loudness - Sound pressure level [sustained phonation subtask] | Y | Y | 6 |
| Nascimento_a | Brazil | Physiotherapy | Not Reported | 11 | PROSPERO CRD42019130988 | N | N | Y | Walking speed and balance | N | Walking speed | Y | Y | 2 |
| Nascimento_b | Brazil | Physiotherapy | No Profit | 13 | PROSPERO CRD42018108419 | N | Y | Y | Walking speed, balance, and strength | N | Walking speed | Y | Y | 5 |
| Naunton | Australia | Clinical rehabilitation | None | 7 | PROSPERO CRD42019136513 | Y | Y | N | Composite pain and function, overall pain, pain with activity, and pain at rest, number of participants experiencing an adverse event | Y | Composite pain and function | Y | Y | 4 |
| Navarro-Santana | Spain | Physiotherapy | None | 18 | PROSPERO CRD42018089991 | N | Y | N | Any outcome evaluating the function of the sympathetic nervous system (i.e. skin conductance or skin temperature) was evaluated. | Y | changes in skin conductance | Y | Y | 18 |
| Nayak | India | Complementary therapies in clinical practice | None | 11 | PROSPERO CRD42018104315 | N | N | Y | Balance and gait | Y | Balance [Berg Balance Scale] | Y | Y | 3 |
| Oliveira | Brazil | The Cochrane database of systematic reviews | No Profit | 2 | Protocol (doi: 10.1002/14651858.CD013018) | Y | Y | N | 1: QoL; pain; trunk deformity 2: Treatment success; Change in pulmonary function; Change in functional capacity; Adverse effects; Adherence to treatment; Ease of access to intervention | Y | Quality of life | Y | Y | 2 |
| Orgeta | England | The Cochrane database of systematic reviews | No Profit | 7 | Protocol (doi: 10.1002/14651858.CD011961) | Y | Y | N | 1: Measures of cognitive function: global cognition, executive function, attention, memory (specifically verbal memory), and visual processing 2: Measures of function (e.g. activities of daily living); Measures of quality of life; Measures of neuropsychiatric symptoms including depression, anxiety, and apathy assessed by a validated rating scale; Measures of carer outcomes including quality of life, experience of carer burden, well-being, or mood; Adverse effects (e.g. on mood, awareness of cognitive difficulties) | Y | Global cognition | Y | Y | 6 |
| Pan | China | International journal of surgery (London, England) | None | 6 | RESEARCH REGISTRY (Reviewregistry884) | N | N | Y | VAS, WOMAC and range of motion, LKSS, KOOS and postoperative complications. | Y | Pain [VAS] | Y | Y | 5 |
| Paravlic | Slovenia | Archives of physical medicine and rehabilitation | Not Reported | 7 | PROSPERO CRD42019118886 | N | N | N | Both objective and subjective measures of patients functional recovery and health status will be included. Gait parameters, maximal muscle strength and/or power, balance, self-reported measures of functional recovery assessed by questionnaires (e.g., Oxford Knee Score, Lower Extremity Functional Score, SF-12-36 etc.) self perceived pain. | N | physical performance in general | Y | Y | 7 |
| Parmenter | Australia | British journal of sports medicine | None | 18 | PROSPERO CRD42017081184 | N | N | N | Claudication onset distance, total walking distance, muscle strength | Y | Claudication onset distance | Y | Y | 8 |
| Pazzianotto-Forti | Brazil | Physical therapy | None | 26 | PROSPERO CRD42017068169 | N | N | Y | Outcomes related to physical fitness (ie, muscular strength, muscular endurance, cardiovascular endurance, and flexibility) | Y | Walking speed | N | N | 2 |
| Plaza-Manzano | Spain | European journal of pain (London, England) | Not Reported | 19 | PROSPERO CRD42019131331 | N | Y | N | Pain intensity, disability | Y | Pain intensity | Y | Y | 9 |
| Pogrebnoy | Australia | Archives of physical medicine and rehabilitation | Not Reported | 8 | PROSPERO CRD42018109506 | N | N | Y | mobility outcomes which are measured objectively (Eg: 6minWT, TUG, 10mwt) or improve physical activity levels as measured by step count, minutes of vigorous, moderate or light activity or metabolic equivalents (METS). | N | Habitual walking speed | Y | Y | 5 |
| Pozuelo-Carrascosa | Spain | International journal of environmental research and public health | No Profit | 19 | PROSPERO CRD42020182082 | N | Y | Y | Respiratory function, respiratory muscle strength and functional capacity (walking ability, dyspnea, balance, activities of daily life) | Y | First second forced expiratory volume (FEV1) | Y | Y | 11 |
| Prosperini | Italy | Journal of neurology | No Profit | 41 | PROSPERO CRD42020161568 | Y | N | Y | Balance | Y | Balance | Y | Y | 41 |
| Riberholt | Denmark | PloS one | No Profit | 5 | PROSPERO CRD42018088790 | Y | Y | N | Mortality or poor functional outcome, Serious adverse events, Non-serious adverse events, Level of consciousness. | Y | Mortality or poor functional outcome | Y | Y | 4 |
| Roberts | Australia | The Cochrane database of systematic reviews | None | 7 | Protocol (doi: 10.1002/14651858.CD012988) | Y | Y | Y | Symptoms of AIMSS (pain, stiffness, and grip strength), safety (including adverse events), incidence of AIMSS, Persistence and compliance of women continuing to take their AI medication due to the intervention, health-related quality of life, cancer-specific quality of life, cancer-specific survival, overall survival | Y | Overall change in worst pain scores | Y | Y | 4 |
| Robson | Australia | The Journal of orthopaedic and sports physical therapy | None | 44 | PROSPERO CRD42016043134 | Y | N | Y | Pain intensity and disability, weight, body mass index, physical performance measures, physical activity, dietary outcomes, mental health, and quality of life | Y | Pain intensity | Y | Y | 10 |
| Rueda | Spain | The Cochrane database of systematic reviews | None | 9 | Protocol (doi: 10.1002/14651858.CD013449) | Y | Y | Y | 1: Daytime sleepiness, Morbidity and mortality. 2: Quality of life, Sleep quality, Adverse events and side effects, Apnoea-Hypopnoea Index (AHI) and Snoring | Y | Daytime sleepiness | Y | Y | 2 |
| Sabe | Switzerland | General hospital psychiatry | None | 17 | PROSPERO CRD42019133359 | Y | N | N/A | Functional capacity, change in peak oxygen consumption (VO2 peak) | Y | maximal oxygen consumption | Y | Y | 23 |
| Saunders | England | The Cochrane database of systematic reviews | Not Reported | 75 | Previous review (doi: 10.1002/14651858.CD003316.pub6) | Y | Y | N | Death, death or dependence, disability, adverse events, vascular risk factors, physical fitness, mobility, physical function, health status and quality of life, mood, cognitive function | Y | Death | Y | Y | 32 |
| Shahabi | Iran | Clinical rehabilitation | None | 14 | PROSPERO CRD42018111421 | Y | Y | Y | Walking speed, balance, walking ability, functional mobility, independence of walking, postural sway | Y | Walking speed | Y | Y | 3 |
| Silva | Brazil | The Cochrane database of systematic reviews | No Profit | 21 | Protocol (doi: 10.1002/14651858.CD013019) | Y | Y | Y | 1: ability to walk (walking speed and dependence on personal assistance) 2: Walking endurance, Motor function, Functional mobility, Adverse events | Y | Walking speed | Y | Y | 6 |
| Singh | France | The international journal of behavioral nutrition and physical activity | None | 19 | PROSPERO CRD42020164152 | N | N | N | Safety (adverse events), feasibility (Recruitment, withdrawal and exercise adherence rates), health outcomes (objectively-assessed and/or self-reported QoL, aerobic fitness, fatigue, upper-body strength, lower-body strength, anxiety, depression, sleep, body fat percentage and body mass index). | Y | Safety | Y | Y | 19 |
| Skelly | United States | AHRQ Comparative Effectiveness Reviews | None | 31 | PROSPERO CRD42019132457 | Y | Y | Y | Function/disability/pain interference, pain, Psychological distress (including measures of depression and anxiety), quality of life, opioid use, sleep quality, sleep disturbance, Healthcare utilization | Y | Function | Y | Y | 11 |
| Smith | England | The Cochrane database of systematic reviews | No Profit | 7 | Previous review (doi: 10.1002/14651858.CD010569.pub2) | Y | Y | N | Health-related quality of life, activities of daily living, cognitive function, behaviour, pain, all-cause mortality, adverse events, use of health and social care resources, costs of hospitalisation, hospital readmission, health and social care support in the community or in residential or nursing home care, and costs to people with dementia who have had a hip fracture and to their carers | Y | All-cause mortality | Y | N | 3 |
| Su | China | Journal of advanced nursing | None | 14 | PROSPERO CRD42018116445 | N | N | Y | Behavioural, physiological and clinical outcomes | Y | Physical activity time | Y | Y | 4 |
| Surace | Australia | The Cochrane database of systematic reviews | No Profit | 32 | Protocol (doi: 10.1002/14651858.CD008962) | Y | Y | N | Participant-reported pain relief of 30% or greater, Mean pain score or mean change in pain score, Disability or function, Composite endpoints measuring 'success' of treatment such as participants feeling no further symptoms, Quality of life, Number of participant withdrawals, Number of participants experiencing any adverse event, Proportion of participants achieving pain score below 30/100 mm on VAS, ROM active preferred over passive measures, size of the calcification, number of participants with complete or partial resolution (defined or not) of calcific deposits | Y | Mean pain score or mean change in pain score | Y | N | 6 |
| Takahashi | Japan | Journal of the American Medical Directors Association | None | 10 | PROSPERO CRD42016053313 | N | N | N | Mortality, complications, muscle strength, activities of daily living (ADL), QOL, falls, re-fractures, and adverse events (pneu- monia, liver failure, kidney failure). | Y | Mortality | Y | N | 4 |
| Tomazoni | Brazil | Journal of physiotherapy | No Profit | 12 | PROSPERO CRD42018088242 | Y | Y | N | Pain intensity, disability, Overall improvement or satisfaction with treatment, Quality of life, Work status, Adverse events | Y | Pain | Y | Y | 5 |
| van Nispen | Netherlands | The Cochrane database of systematic reviews | No Profit | 44 | Protocol (doi: 10.1002/14651858.CD006543) | Y | Y | Y | Health-related QOL (HRQOL), vision-related QOL (VRQOL) or related patient-reported outcomes | Y | Vision-related quality of life | Y | Y | 2 |
| Waldauf | Czech Republic | Critical care medicine | Mixed | 43 | PROSPERO CRD42019132255 | Y | N | Y | Outcomes included ICU- and end-of-study mortality (defined as mortality at the last follow-up point), the length of stay (LOS) in ICU and in hospital, the duration of mechanical ventilation and/or ventilator-free days at day 28, and any long-term functional outcome. | Y | Mortality | Y | Y | 38 |
| Wang | China | International journal of nursing studies | Not Reported | 39 | PROSPERO CRD42020167189 | Y | N | N | Main: intensive care unit-acquired weakness rate, the length of mechanical ventilation, the length of ICU stay (care unit stay), the length of hospital stay, ICU mortality, hospital mortality Additional: MRC scores, handgrip strength, Barthel Index score, the incidence of VAP, DVT, pressure sore and ICU-delirium, MCS and PCS of SF-36 | Y | Intensive care unit-acquired weakness rate | Y | Y | 8 |
| Wilhelm | United States | The Journal of orthopaedic and sports physical therapy | None | 14 | PROSPERO CRD42017063956 | N | N | Y | Pain and disability | Y | Pain | Y | Y | 13 |
| Woodley | England | The Cochrane database of systematic reviews | No Profit | 46 | Previous review (doi: 10.1002/14651858.CD007471.pub3) | Y | Y | Y | 1: Self-reported urinary or faecal incontinence, Urinary incontinence-specific quality of life, Faecal incontinence-specific quality of life 2: Self-reported severity of incontinence, Number of urinary or faecal incontinence episodes, Loss of urine under stress test, Self-reported measures of pelvic floor dysfunction, Other self-reported well-being measures, Adverse effects, particularly discomfort or pain associated with PFMT., Labour and delivery outcome for women who did antenatal PFMT. | Y | Urinary incontinence in late pregnancy | Y | Y | 6 |
| Xie | China | Integrative medicine research | No Profit | 30 | PROSPERO CRD42018117216 | N | N | Y | 1: The overall response rate measured through the Frenchay Dysarthria Assessment (FDA). 2: speech intelligibility and quality of life (measured using a validated questionnaire). Adverse events and serious adverse events | Y | Clinical response rate based on the FDA | Y | Y | 17 |
| Xu | China | Journal of the American Heart Association | No Profit | 6 | PROSPERO CRD42019128366 | N | N | N/A | 1: The overall response rate measured through the Frenchay Dysarthria Assessment (FDA). 2: speech intelligibility and quality of life (measured using a validated questionnaire). Adverse events and serious adverse events | Y | Disease-specific biomarkers (serum NT-proBNP) | Y | Y | 3 |
| Yan | China | PloS one | No Profit | 15 | PROSPERO CRD42019137191 | N | N | Y | 1: The overall response rate measured through the Frenchay Dysarthria Assessment (FDA). 2: speech intelligibility and quality of life (measured using a validated questionnaire). Adverse events and serious adverse events | Y | General cognitive mental status [Minimum Mental State Examination (MMSE) or the Montreal Cognitive Assessment (MoCA)] | Y | Y | 10 |
| Yang | China | Physical therapy | No Profit | 16 | PROSPERO CRD42018109786 | Y | N | N | Pain, stiffness, physical function, and QOL | Y | Pain | Y | Y | 15 |
| Yau | China | Annals of physical and rehabilitation medicine | None | 12 | PROSPERO CRD42019127780 | Y | N | N | Perioperative mortality, postoperative complications, quality of recovery, physical or functional performance, psychosocial status, frailty level and healthcare utilisation | Y | Perioperative mortality | N | N | 4 |
| Ye | China | Frontiers in physiology | No Profit | 7 | PROSPERO CRD42020162738 | N | N | N | Echocardiographic measures (LVEF, left ventricular end diastolic dimension [LVEDD]), exercise capacity (peak VO2, exercise duration), HRQOL, and/or adverse events (all-cause mortality, serious adverse events). | N | All-cause mortality | Y | Y | 3 |
| Yeh | Taiwan | Multiple sclerosis and related disorders | No Profit | 10 | PROSPERO CRD42019128766 | Y | N | N | Walking performance (gait parameters, balance, and ambulation capability), perceived fatigue, spasticity, global mobility, physical QOL, mental QOL, pain, ADL, Expanded Disability Status Scale (EDSS), and treatment acceptance | Y | Gait speed | Y | Y | 8 |
| Yoo | South Korea | Medicine | None | 8 | PROSPERO CRD42019093590 | N | N | Y | Pain intensity | Y | Pain intensity | Y | Y | 5 |
| Yu | China | American journal of physical medicine & rehabilitation | No Profit | 11 | PROSPERO CRD42019128161 | N | N | Y | Pain, depression and anxiety | Y | Pain | Y | Y | 4 |
| Yue | China | Journal of advanced nursing | No Profit | 17 | PROSPERO CRD42020161124 | N | N | Y | Heart rate, respiratory rate, and oxygen saturation. second outcomes: behavioural state, stress level, oral feeding volume, and maternal anxiety. | Y | Heart rate | Y | Y | 11 |
| Zhao_a | China | BioMed research international | No Profit | 16 | PROSPERO CRD42018095266 | Y | N | Y | Functional capacity, health-related QoL, pulmonary function, symptoms, acute exacerbations | Y | Functional capacity [6MWD] | Y | Y | 12 |
| Zhao_b | China | Psychology, health & medicine | No Profit | 5 | PROSPERO CRD42017074879 | N | N | Y | Pain, Bath Ankylosing Spondylitis Disease Activity Index (BASDAI), Bath Ankylosing Spondylitis Functional Index (BASFI) | Y | Pain | Y | Y | 4 |
| Zhou | China | Medicine | No Profit | 11 | PROSPERO CRD42018093902 | Y | N | Y | General cognition, executive functions, memory, language ability, and visuospatial ability | Y | General cognition [MMSE and MoCA scores] | N | N | 6 |
| Zhu | China | PloS one | No Profit | 18 | PROSPERO CRD42020159865 | Y | N | Y | Pain, disability, quality of life | Y | Pain | Y | Y | 6 |
| Ziebart | Canada | Archives of rehabilitation research and clinical translation | No Profit | 8 | PROSPERO CRD42019133515 | Y | N | N | Number of falls, incidence of falls, number of recurrent falls, fall hazard identification | Y | Incidence of falls | Y | N | 5 |

***Abbreviations***: N= No; N/A= Not Available; Y= Yes
