# Supplementary material for: Reasons for missing evidence in rehabilitation meta-analyses: a cross-sectional meta-research study
Source: BMC Med Res Methodol. 2023 Oct 21;23:245. doi: 10.1186/s12874-023-02064-7 (PMC10590516; doi:10.1186/s12874-023-02064-7)
Supplement: Supplementary file 3 — Additional file 3: Appendix 3. Assessment of the reason for omission. [file 12874_2023_2064_MOESM3_ESM.docx]

**Appendix 3 - Assessment of the reason for omission**

***Legend***: **1=** Review author; **2=** Author and year of publication of primary study; **3=** Is the study included in the systematic review?; **4=** Is the protocol available?; **5=** Agreement between protocol and publication on the review primary outcome; **6=** Reasons for omission; **7=** Justification for judgements.

| 1 | 2 | 3 | 4 | 5 | 6 | 7 |
| --- | --- | --- | --- | --- | --- | --- |
| Alghamdi | Aggarwal 2010 | Y | N | NA | D | Study report does not mention the outcome of interest and protocol not available for assessment. |
| Alghamdi | Cegla 2002 | Y |  |  | G | Article in German |
| Alghamdi | McCarroll 2005 | Y | N | NA | E | The outcome was measured with a different scale (the Pulmonary Function Status Scale) and therefore not included in meta-analysis. Only mean values have been reported with no standard deviation, yet the individual patient data have been provided. |
| Alghamdi | Svenningsen 2016 | Y | Y | Y | C | Outcome data reported for sub-population (sputum produced and not-sputum produced) and not for different intervention. |
| Alghamdi | Weiner 1996 | Y |  |  | H |  |
| Alghamdi | Wolkove 2004 | Y | N | NA | D | Study report does not mention the outcome of interest and protocol not available for assessment. |
| Almeida | McGarvey 2015 | Y | N | NA | C | The outcome measure is made of two subscales, yet the authors reported the overall score only, thus preventing the study from being included in meta-analysis. |
| Amedoro | Aidar 2018 | Y | N | NA | D | Study report does not mention the outcome of interest and protocol not available for assessment. |
| Amedoro | Bansi 2012 | Y | N | NA | E | The outcome was measured with a scale and at a follow-up timepoint different than the ones considered by the systematic review. |
| Amedoro | Bayraktar 2013 | Y | N | NA | E | The outcome was measured with a different scale than the one considered by the systematic review. |
| Amedoro | Castro-Sànchez 2012 | Y | N | NA | E | The outcome was measured at a different timepoint than the one considered by the systematic review. |
| Amedoro | Hejazi 2012 | Y | N | NA | D | Study report does not mention the outcome of interest and protocol not available for assessment. |
| Amedoro | Hejazi 2012b | Y | N | NA | D | Study report does not mention the outcome of interest and protocol not available for assessment. |
| Amedoro | Marandi 2013 | Y | N | NA | D | Study report does not mention the outcome of interest and protocol not available for assessment. |
| Amedoro | Razazian 2016 | Y | N | NA | E | The outcome was measured with a different scale than the one considered by the systematic review. |
| Arora | Adegoke 2001 | Y | N | NA | D | Study report does not mention the outcome of interest and protocol not available for assessment. |
| Arora | Ahmad 2008 | Y | N | NA | D | Study report does not mention the outcome of interest and protocol not available for assessment. |
| Arora | Baker 1996 | Y | N | NA | E | The trial has not been included because, quoting the review authors, "These data are not reliable as participants from the control group were reassigned to Experimental 1 or Experimental 2 after 28 days leading to duplication of data", but were available in the paper. |
| Arora | Carley 1985 | Y | N | NA | D | Study report does not mention the outcome of interest and protocol not available for assessment. |
| Arora | García-Pérez 2018 | Y | N | NA | D | Study report does not mention the outcome of interest and protocol not available for assessment. |
| Arora | Gentzkow 1991 | Y | N | NA | D | Study report does not mention the outcome of interest and protocol not available for assessment. |
| Arora | Jercinovic 1994 | Y | N | NA | D | Study report does not mention the outcome of interest and protocol not available for assessment. |
| Arora | Karba 1995 | Y | N | NA | D | Study report does not mention the outcome of interest and protocol not available for assessment. |
| Arora | Kloth 1998 | Y | N | NA | D | Study report does not mention the outcome of interest and protocol not available for assessment. |
| Avendaño-Coy | Lizis 2017 | Y | N | NA | E | The outcome was measured with a different scale (the WOMAC Pain subscale) and therefore not included in meta-analysis. |
| Azambuja | Stein 2009 | Y | N | NA | E | Secondary analysis of a previous study (Dall'Ago 2006) already included in meta-analysis, which reported the outcome of interest. |
| Barclay | Page 2000 | Y | N | NA | D | Study report does not mention the outcome of interest and protocol not available for assessment. |
| Barclay | Page 2009 | Y | N | NA | F | The outcome data have been measured and are available, yet the review authors "noted that Page 2009 alone appeared to be a cause of much of the heterogeneity for the upper extremity activity outcome" and therefore "decided to remove Page 2009 from the analysis of the upper extremity activity outcome in all comparisons and subgroups of mental practice in addition to other treatment versus other treatment (± placebo)". |
| Barclay | Page 2011 | Y | N | NA | C | The outcome data have been reported as means only with no standard deviation or measure of variability. |
| Barclay | Sun 2013 | Y | N | NA | D | Study report does not mention the outcome of interest and protocol not available for assessment. |
| Barclay | Wang 2019 | Y | Y | Y | A | Study report and protocol do not mention the outcome of interest. |
| Biazus-Sehn | Baker 2010 | Y | N | NA | D | The outcome was measured at baseline. Study report does not mention the outcome of interest as a follow up measure and protocol not available for assessment. |
| Biazus-Sehn | Brinke 2015 | Y | Y | NA | A | The outcome measure was used as an inclusion criterion, as stated in the paper "Women [...] were eligible for the study if they: [...] (3) scored ≥24/30 on the Mini-Mental State Examination (MMSE); (4) scored <26/30 on the Montreal Cognitive Assessment (MoCA)", and reported at baseline. Study report does not mention the outcome of interest as a follow up measure and protocol only states "Cognitive performance after 3 and 6 months of training" as primary outcome measure. |
| Biazus-Sehn | Damirchi 2018 | Y | N | NA | D | The outcome measure was used as an inclusion criterion, as stated in the paper " [...] considering the following inclusion criteria: [...] (3) Global Deterioration Scale–Short Form (GDS-SF) level 0 to 5" and "In order to diagnose MCI, Mini-Mental State Examination (MMSE) [...] was used here", and reported at baseline. Study report does not mention the outcome of interest as a follow up measure and protocol not available for assessment. |
| Biazus-Sehn | Davis 2013 | Y | Y | NA | A | The outcome measure was used as an inclusion criterion, as stated in the paper "Participants enrolled in the EXCEL study had a Mini Mental State Examination (MMSE) score >=24". Study report does not mention the outcome of interest as a follow up measure and protocol only states "Cognitive performance after 3 and 6 months of training" as primary outcome measure. |
| Biazus-Sehn | Hildreth 2015 | Y | Y | Y | E | The outcome data have been reported as mean change and 95% confidence interval, as stated as pag 56 "The only statistically significant difference in the secondary cognitive outcomes was greater improvement on the ADAS-Cog in EET [–1.6, 95% confidence interval (CI) –4.9, 1.6] compared with CON (–0.3, 95% CI –3.5, 3.0; p = 0.05)". |
| Biazus-Sehn | Nagamatsu 2012 | Y | Y | NA | A | The outcome measure was used as an inclusion criterion, as stated in the paper "Participants were classified as having probable mild cognitive impairment if they had a score lower than 26 out of 30 on the Montreal Cognitive Assessment", and reported at baseline. Study report does not mention the outcome of interest as a follow up measure and protocol only states "Cognitive performance after 3 and 6 months of training" as primary outcome measure. |
| Biazus-Sehn | Nagamatsu 2013 | Y | Y | NA | A | The outcome measure was used as an inclusion criterion, as stated in the paper "Women [...] were eligible for study entry if they: [...] (3) scored ≥24/30 on the Mini-Mental State Examination (MMSE); (4) scored <26/30 on the Montreal Cognitive Assessment (MoCA)", and reported at baseline. Study report does not mention the outcome of interest as a follow up measure and protocol only states "Cognitive performance after 3 and 6 months of training" as primary outcome measure. |
| Biazus-Sehn | Scherder 2005 | Y | N | NA | D | The outcome was used as inclusion criterion, as stated in the paper "Subjects were included in the study if they had an MMSE score of 7 or higher", and measured at baseline. Study report does not mention the outcome of interest as a follow up measure and protocol not available for assessment. |
| Biazus-Sehn | Sungkarat 2016 | Y | N | NA | D | The outcome was used as inclusion criterion, as stated in the paper "Participants were [...] included in the study if [...] had scores of 24 or greater on the Mini-Mental State Examination (MMSE) and less than 26 on the Montreal Cognitive Assessment (MoCA)", and reported at baseline. Study report does not mention the outcome of interest as a follow up measure and protocol not available for assessment. |
| Biazus-Sehn | van Uffelen 2008 | Y | Y | NA | A | The outcome was measured and reported at baseline only, as stated in the paper "The mini mental state examination (MMSE) was used to measure general cognitive function for descriptive purposes". Study report does not mention the outcome of interest as a follow up measure and protocol only states "Cognitive functioning" as primary outcome measure. |
| Biazus-Sehn | Xia 2019 | Y | N | NA | D | The outcome was measured and reported at baseline only. Study report does not mention the outcome of interest as a follow up measure and protocol not available for assessment. |
| Bjarnason-Wehrens | Austin 2005 | Y | N | NA | D | Study report does not mention the outcome of interest and protocol not available for assessment. Mortality has been reported as number of deaths in each group. |
| Bjarnason-Wehrens | Dalal 2018 | Y | Y | Y | A | Study report and protocol do not mention the outcome of interest. Mortality has been reported as number of deaths in each group. |
| Bjarnason-Wehrens | de Meirelles 2014 | Y | N | NA | D | Study report does not mention the outcome of interest and protocol not available for assessment. |
| Bjarnason-Wehrens | de Mello Franco 2006 | Y | N | NA | D | Study report does not mention the outcome of interest and protocol not available for assessment. Mortality has been reported as number of deaths in each group. |
| Bjarnason-Wehrens | Dehkordi 2015 | Y | N | NA | D | Study report does not mention the outcome of interest and protocol not available for assessment. |
| Bjarnason-Wehrens | Dracup 2007 | Y | N | NA | D | Study report does not mention the outcome of interest and protocol not available for assessment. Mortality has been reported as number of deaths in each group. |
| Bjarnason-Wehrens | Ellingsen 2016 | Y | Y | Y | A | Study report and protocol do not mention the outcome of interest. Mortality has been reported as number of deaths in each group. |
| Bjarnason-Wehrens | Flynn 2009 | Y | Y | Y | E | Paper reporting the secondary outcomes of O'Connor 2009, already included in meta-analysis. |
| Bjarnason-Wehrens | Giannuzzi 2003 | Y | N | NA | D | Study report does not mention the outcome of interest and protocol not available for assessment. Mortality has been reported as number of deaths in each group. |
| Bjarnason-Wehrens | Hambrecht 2000 | Y | N | NA | D | Study report does not mention the outcome of interest and protocol not available for assessment. Mortality has been reported as number of deaths in each group. |
| Bjarnason-Wehrens | Höllriegel 2016 | Y | Y | Y | A | Study report and protocol do not mention the outcome of interest. Mortality has been reported as number of deaths in each group. |
| Bjarnason-Wehrens | Jolly 2009 | Y | Y | Y | A | Study report and protocol do not mention the outcome of interest. Mortality has been reported as number of deaths in each group. |
| Bjarnason-Wehrens | Keteyian 1999 | Y | N | NA | D | Study report does not mention the outcome of interest and protocol not available for assessment. Mortality has been reported as number of deaths in each group. |
| Bjarnason-Wehrens | Klecha 2007 | Y | N | NA | D | Study report does not mention the outcome of interest and protocol not available for assessment. It has been reported that no death occurred. |
| Bjarnason-Wehrens | Klocek 2005 | Y | N | NA | D | Study report does not mention the outcome of interest and protocol not available for assessment. |
| Bjarnason-Wehrens | Koukouvou 2004 | Y | N | NA | D | Study report does not mention the outcome of interest and protocol not available for assessment. |
| Bjarnason-Wehrens | Lang 2018 | Y | Y | Y | A | Study report and protocol do not mention the outcome of interest. |
| Bjarnason-Wehrens | McKelvie 2002 | Y | N | NA | D | Study report does not mention the outcome of interest and protocol not available for assessment. Mortality has been reported as number of deaths in each group. |
| Bjarnason-Wehrens | Myers 2000 | Y | N | NA | D | Study report does not mention the outcome of interest and protocol not available for assessment. Mortality has been reported as number of deaths in each group. |
| Bjarnason-Wehrens | Nilsson 2008 | Y | N | NA | D | Study report does not mention the outcome of interest and protocol not available for assessment. Mortality has been reported as number of deaths in each group. |
| Bjarnason-Wehrens | Norman 2012 | Y | N | NA | D | Study report does not mention the outcome of interest and protocol not available for assessment. Mortality has been reported as number of deaths in each group. |
| Bjarnason-Wehrens | Passino 2006 | Y | N | NA | D | Study report does not mention the outcome of interest and protocol not available for assessment. |
| Bjarnason-Wehrens | Sabelis 2004 | Y | N | NA | D | Study report does not mention the outcome of interest and protocol not available for assessment. It has been reported that no death occurred. |
| Bossen | Armbrust 2017 | Y | N | NA | C | Outcome data have been reported as median and interquartile range, but no justification for doing so has been provided. |
| Bossen | Christison 2016 | Y | Y | Y | E | The outcome was measured in a different way (step counts) and therefore not included in meta-analysis. |
| Bricca | Abdelbasset 2019 | Y | N | NA | D | Study report does not mention the outcome of interest and protocol not available for assessment. |
| Bricca | Blumenthal 2012a | Y | Y | Y | A | Study report and protocol do not mention the outcome of interest. |
| Bricca | Blumenthal 2012b | Y | Y | N | B | Study protocol mentions the outcome to be measured "at baseline, months 3, 6, 9, 12, 15, 18, 21, 24, 36, and end of study", but study report does not mention it among the outcomes and reports it only at baseline. The outcome of interest was actually reported in different publications, which were not included in the systematic review, nor cited in the trial. |
| Bricca | Edelmann 2011 - Nolte 2014 | Y | Y | Y | F | The outcome was measured with one of the questionnaires considered by the SR authors (The Minnesota living with heart failure questionnaire), yet it was not included in meta-analysis. |
| Bricca | Gretebeck 2019 | Y | Y | Y | A | Study report and protocol do not mention the outcome of interest. |
| Bricca | Keihani 2014 | Y | N | NA | E | The outcome was measured with a different scale (the SF-36 questionnaire) and therefore not included in meta-analysis. |
| Bricca | Pibernik-Okanovic 2015 | Y | Y | Y | C | The outcome was measured with a scale (the SF-12v2 questionnaire) made of two subscales, yet the authors reported the score of one of them only, thus preventing the study from being included in meta-analysis. |
| Bricca | Rodriguez-Manas 2019 | Y | Y | Y | E | The outcome was measured with one of the questionnaires considered by the SR authors (the European Quality of Life-5 Dimensions-5 Levels (EQ-5D-5 L) questionnaire), but the results have been reported as mean difference (95% CI) and it was not included in meta-analysis. |
| Bricca | Schneider 2016 | Y | Y | N | B | Study protocol (PMID: 21765864) reports that "Quality of life will be assessed using the Short Form-v36", yet the study report does not mention it among the outcomes. |
| Bricca | Soliman 2019 | Y | N | NA | D | Study report does not mention the outcome of interest and protocol not available for assessment. |
| Burge | De Roos 2017 | Y | N | NA | D | Study report does not mention the outcome of interest and protocol not available for assessment. |
| Burge | Lum 2007 | N | N | NA | D | Study report does not mention the outcome of interest and protocol not available for assessment. |
| Burge | Rodriguez-Trigo 2011 | N | N | NA | D | Conference abstract only. Study report does not mention the outcome of interest and protocol not available for assessment. |
| Burge | Roman 2013 | N | Y | Y | A | Study report and protocol do not mention the outcome of interest. |
| Candelaria | Bettencourt 2005 | Y |  |  | H |  |
| Candelaria | Devi 2014 | Y | N | NA | E | The outcome is assessed with a different outcome measure (MacNew) and therefore not included in meta-analysis. |
| Candelaria | Hautala 2017 | Y | Y | Y | E | The outcome is assessed with a different outcome measure (5D questionnaire) and therefore not included in meta-analysis. |
| Candelaria | Houle 2012 | Y | N | NA | E | The outcome is assessed with a different outcome measure (Quality of Life Index-cardiac version III) and therefore not included in meta-analysis. |
| Candelaria | Mutwalli 2012 | Y | N | NA | C | The outcome is reported as a overall score and the subscores are not reported. |
| Candelaria | Oerkild 2012 | Y | Y | Y | C | SDs are not available |
| Candelaria | Reid 2012 | Y | N | NA | E | The outcome is assessed with a different outcome measure (MacNew) and therefore not included in meta-analysis. |
| Candelaria | Sandstrom 2005 | Y | N | NA | E | The outcome is assessed with different outcome measures (the EuroQol and Time Trade Off questionnaires) and therefore not included in meta-analysis. |
| Candelaria | Santaularia 2017 | Y | Y | Y | E | The outcome is assessed with a different outcome measure (EuroQoL-5D) and therefore not included in meta-analysis. |
| Casey | Alaranta 1994 | Y | N | NA | D | The outcome (pain) is measured together with another one (disability) on a cumulative index (Million index), which was considered a measure of disability by the review authors and therefore not included in meta-analysis. |
| Casey | Bendix 1995 | Y | N | NA | E | The first assessment was made at 4 months, exceeding the short-term follow-up (3 months) and therefore not included in meta-analysis at 3 months. |
| Casey | Bendix 1997 | Y | N | NA | E | One-year follow-up of Bendix 1995, already assessed (and not included in meta-analysis because the assessment timepoint exceeded the 3 months follow up). |
| Casey | Bendix 1998 | Y | N | NA | E | Two-year follow-up of Bendix 1995, already assessed (and not included in meta-analysis because the assessment timepoint exceeded the 3 months follow up). |
| Casey | Bendix 1998 | Y | N | NA | E | Five-year follow-up of Bendix 1995, already assessed (and not included in meta-analysis because the assessment timepoint exceeded the 3 months follow-up). |
| Casey | Bendix 2000 | Y | N | NA | E | The first assessment was made at 12 months, exceeding the short-term follow-up (3 months) and therefore not included in meta-analysis at 3 months. |
| Casey | Jousset 2004 | Y | N | NA | E | The first assessment was made at 6 months, exceeding the short-term follow-up (3 months) and therefore not included in meta-analysis at 3 months. |
| Casey | Pato 2010 | Y | N | NA | C | Trial authors did not report data separately for the intervention and control groups relevant to the review and the study was therefore not included in the meta-analysis. |
| Casey | Roche-Leboucher 2011 | Y | N | NA | D | Follow-up of Roche 2007, already included in meta-analysis. |
| Casey | Ronzi 2017 | Y | Y | Y | E | The first assessment was made at 12 months, exceeding the short-term follow-up (3 months) and therefore not included in meta-analysis at 3 months. |
| Casey | Schweikert 2006 | Y | N | NA | E | The outcome was measured and reported as mean change and standard deviation, yet it was not included in meta-analysis since the review authors found results in the text and in the tables not consistent with each other. |
| Casey | Smeets 2008 | Y | Y | Y | E | Six- and twelve-month follow-up of Smeets 2006, already included in meta-analysis. |
| Chae | Zhang 2016 | Y | N | NA | D | Study report does not mention the outcome of interest and protocol not available for assessment. |
| Chaovalit | Bhatt 2013 | Y | N | NA | D | Study report does not mention the outcome of interest and protocol not available for assessment. |
| Chiu | Gharib 2011 | Y | N | NA | D | Study report does not mention the outcome of interest and protocol not available for assessment. Gross Motor Function Classification System scale has been used at initial assessment. |
| Chiu | Hösl 2018 | Y | N | NA | C | Being a cross-over trial, no separate data from the first phase have been reported, thus preventing them to be included in meta-analysis with those from parallel-group trials. |
| Chow | Jeong 2016 | Y | Y | Y | E | Data have been reported as mean changes and standard deviations. |
| Chow | Jirayucharoensak 2019 | Y | N | NA | E | Data have been analyzed as repeated measures Time × treatment. |
| Chow | Park 2019 | Y | Y | Y | B | Data have been reported at 24 weeks follow-up only and not at the end of intervention (12 weeks). |
| Chow | Rovner 2018 | Y | Y | Y | A | Trial primary outcome is a "dichotomous variable based on the Hopkins Verbal Learning Test-Revised (HVLT-R) Total Recall measure (i.e., loss of ≥ 6 points) at 6, 12, 18, and 24 months", so the outcome of interest has not been planned to be measured nor measured. |
| Chow | Vidovich 2015 | Y | Y | Y | E | Data have been reported as mean changes and 95% Confidence Interval. |
| de Almeida | Callahan 2017 | Y | Y | N | B | Registered protocol (NCT01314950) lists the outcome as follows "Mini Mental State Examination [Time Frame: Baseline, 6 months, 12 months, 18 months, and 24 months]", but no results have been provided in the published manuscript. |
| de Almeida | D'Amico 2016 | Y | Y | Y | A | Outcome measured at baseline. Study report and protocol do not mention the outcome of interest as a follow up measure. |
| de Almeida | Dawson 2017 | Y | N | NA | D | Outcome measured at baseline. Study report does not mention the outcome of interest as a follow up measure and protocol not available for assessment. |
| de Almeida | Lowery 2014 | Y | Y | Y | A | Outcome measured at baseline. Study report and protocol do not mention the outcome of interest as a follow up measure. |
| de Almeida | McCurry 2011 | Y | Y | Y | A | Outcome measured at baseline. Study report and protocol do not mention the outcome of interest as a follow up measure. |
| de Almeida | Öhman 2017 | Y | N | NA | D | "This article evaluates the effects of exercise on NPS, depression, and institutionalizations in AD patients." Secondary analysis, outcome of interest not considered. |
| de Almeida | Padala 2017 | Y | N | NA | E | "There were no significant inter-group or intra-group differences for any of the other secondary outcomes". MMSE score reported as mean changes and 95% confidence intervals from baseline at 8 and 16 weeks and not as absolute values. |
| de Almeida | Pitkälä 2013 | Y | Y | N | A | Outcome measured at baseline. Study report and protocol do not mention the outcome of interest as a follow up measure. |
| de Almeida | Prick 2017 | Y | Y | Y | A | Study report and protocol do not mention the outcome of interest as a follow-up measure. |
| de Almeida | Steinberg 2009 | Y | N | NA | E | "Participants in the exercise group had a higher MMSE than those in the control group (Table 1: 21 vs 16 respectively, p = 0.02). Because MMSE significantly differed between the two groups, the main analyses included MMSE as a covariate". The outcome was supposed to be measured at 6 and 12 weeks, but considering the difference at baseline, it was not included between the secondary outcomes. |
| de Almeida | Suttanon 2013 | Y | Y | Y | A | Study report and protocol do not mention the outcome of interest. |
| de Almeida | Teri 2003 | Y | N | NA | E | "Patient cognitive status was assessed using the MMSE. No change was hypothesized in the MMSE as a function of treatment; it was obtained for descriptive purposes" |
| de Almeida | Wesson 2013 | Y | Y | Y | A | Outcome measured at baseline. Study report and protocol do not mention the outcome of interest as a follow up measure. |
| De Miguel-Rubio | D'Addio 2014 | Y | N | NA | E | Trial measure the outcome with a different scale (SCIM scale) than the one selected in the primary meta-analysis. |
| De Miguel-Rubio | Dimbwadyo-Terrer 2014 | Y | N | NA | E | Trial measure the outcome with a different scale (SCIM scale) than the one selected in the primary meta-analysis. |
| De Miguel-Rubio | Khurana 2017 | Y | N | NA | E | Trial measure the outcome with a different scale (SCIM scale) than the one selected in the primary meta-analysis. |
| De Miguel-Rubio | Prasad 2018 | Y | Y | Y | A | the outcome was not planned at all and nor reported in the trial reports |
| de Oliveira Silva | Kim 2016 | N | Y | Y | F | Outcome data have been presented as mean and SD in the trial paper, yet the review authors did not include it in the meta-analysis "due to inability to retrieve data (after 3 attempts to contact the authors)". |
| Di | Charvet 2017 | Y | Y | Y | A | Study report and protocol do not mention the outcome of interest. |
| Di | Pedullà 2016 | Y | N | NA | D | Study report does not mention the outcome of interest and protocol not available for assessment. |
| Di | Stuifbergen 2012 | Y | N | NA | D | Study report does not mention the outcome of interest and protocol not available for assessment. |
| Di | Stuifbergen 2018 | Y | Y | Y | A | Study report and protocol do not mention the outcome of interest. |
| Di | Tallner 2016 | Y | Y | Y | F | Secondary outcomes were muscle strength, aerobic capacity, lung function, physical activity, and fatigue. Data are presented by mean and SD. |
| Dobler | Cross 2012 | Y | Y | Y | A | No reference to the outcome measure in the trial nor in the protocol as a follow up measure. |
| Dos Santos | Abazar 2015 | Y | N | NA | D | Study report does not mention the outcome of interest and protocol not available for assessment. |
| Dos Santos | Gaeini 2014 | Y | N | NA | E | They measured others types of hormones: DHEASO4 and 17OH–progesterone |
| Duarte | De Ridder 2013 | Y | Y | Y | C | Data have been reported as mean raw improvement as well as the percentage change, but no measure of variability. |
| Duarte | Meier 2015 | Y | Y | NA | C | Outcome data have been reported as median and interquartile range, but no justification for doing so has been provided. |
| Duncan | Bath 2016 | Y | N | NA | E | The systematic review dichotomize the penetration-aspiration scale (PAS) to confirm the presence of aspiration post-intervention with a cut-off of 5, while the trial uses the same scale with a different cut-off (3 out of 8) to assess swallowing safety/aspiration. |
| Duncan | Carnaby 2006 | Y | Y | Y | A | Study report and protocol do not mention the outcome of interest. |
| Duncan | Chen 2016 | Y | Y | Y | A | Study report and protocol do not mention the outcome of interest. |
| Duncan | Du 2016 | Y | Y | Y | A | Study report and protocol do not mention the outcome of interest. |
| Duncan | Dziewas 2018 | Y | Y | Y | A | Study report and protocol do not mention the outcome of interest. |
| Duncan | Hwang 2007 | Y | N | NA | E | Incidence of aspiration was reported as number of patients with aspiration, yet not included in meta-analysis as the review authors only included studies that assessed the incidence of aspiration with a score >5 in the penetration-aspiration scale (PAS). |
| Duncan | Jayasekeran 2010 | Y | N | NA | D | Study report does not mention the outcome of interest and protocol not available for assessment. |
| Duncan | Kumar 2011 | Y | N | NA | D | Study report does not mention the outcome of interest and protocol not available for assessment. |
| Duncan | Li 2018 | Y | N | NA | D | Study report does not mention the outcome of interest and protocol not available for assessment. |
| Duncan | Moon 2017 | Y | N | NA | D | Study report does not mention the outcome of interest and protocol not available for assessment. The systematic review dichotomize the penetration-aspiration scale (PAS) to confirm the presence of aspiration post-intervention, while the trial uses the same scale "to evaluate the swallowing function of patients with dysphagia" and reports mean and standard deviations post-treatment. |
| Duncan | Moon 2018 | Y | N | NA | D | Study report does not mention the outcome of interest and protocol not available for assessment. |
| Duncan | Park 2013 | Y | N | NA | D | Study report does not mention the outcome of interest and protocol not available for assessment. |
| Duncan | Park 2019 | Y | N | NA | D | Study report does not mention the outcome of interest and protocol not available for assessment. |
| Duncan | Suntrup 2015 | Y | Y | Y | A | Study report and protocol do not mention the outcome of interest. |
| Duncan | Suntrup-Kreuger 2018 | Y | Y | Y | A | Study report and protocol do not mention the outcome of interest. |
| Duncan | Vasant 2016 | Y | N | NA | D | The systematic review dichotomize the penetration-aspiration scale (PAS) to confirm the presence of aspiration post-intervention with a cut-off of 5, while the trial uses the same scale with a different cut-off (3 out of 8) to assess swallowing safety. |
| Duncan | Wu 2011 | Y | N | NA | H |  |
| Duncan | Xia 2011 | Y | N | NA | D | Study report does not mention the outcome of interest and protocol not available for assessment. |
| Ebadi | Ansari 2006 | Y | N | NA | D | Study report does not mention the outcome of interest and protocol not available for assessment. |
| Ebadi | Licciardone 2013 | Y | N | NA | C | The outcome has been transformed into a categorical variable, yet being measured as a continuous variable, thus preventing it to be included in meta-analysis. |
| Elsner | Allman 2016 | Y | Y | N | B | The protocol reported the Stroke Impact Scale among the outcome measures, but the trial report does not report it. |
| Elsner | Alves 2017 | N | Y | Y | A | Study report and protocol do not mention the outcome of interest. |
| Elsner | Andrade 2017 | Y | Y | Y | C | The outcome data have been reported as percentage change and therefore not included in the meta-analysis with absolute values. |
| Elsner | Ang 2012 | Y | Y | Y | A | Study report and protocol do not mention the outcome of interest. |
| Elsner | Asseldonk 2016 | N | N | NA | D | Study report does not mention the outcome of interest and protocol not available for assessment. |
| Elsner | Au-Yeung 2014 | Y | N | NA | D | Study report does not mention the outcome of interest and protocol not available for assessment. |
| Elsner | Boggio 2007 | Y | N | NA | D | Study report does not mention the outcome of interest and protocol not available for assessment. |
| Elsner | Celnik 2009 | N | N | NA | D | Study report does not mention the outcome of interest and protocol not available for assessment. |
| Elsner | Chang 2015 | Y | N | NA | D | Study report does not mention the outcome of interest and protocol not available for assessment. |
| Elsner | D'Agata 2016 | Y | Y | Y | A | Study report and protocol do not mention the outcome of interest. |
| Elsner | Danzl 2012 | Y | N | NA | C | The outcome data have been reported as score change (and graphically only) and therefore not included in the meta-analysis with absolute values. |
| Elsner | Fregni 2005 | Y | N | NA | D | Study report does not mention the outcome of interest and protocol not available for assessment. |
| Elsner | Fujimoto 2015 | N | N | NA | D | Study report does not mention the outcome of interest and protocol not available for assessment. |
| Elsner | Fusco 2013 | Y | N | NA | D | Study report does not mention the outcome of interest and protocol not available for assessment. |
| Elsner | Fusco 2014 | Y | N | NA | E | The outcome data have been reported as score change (while absolute values have been reported graphically only) and therefore not included in the meta-analysis with absolute values. |
| Elsner | Geroin 2011 | Y | N | NA | D | Study report does not mention the outcome of interest and protocol not available for assessment. |
| Elsner | Giacobbe 2013 | N | N | NA | D | Study report does not mention the outcome of interest and protocol not available for assessment. |
| Elsner | Goodwill 2015 | N | N | NA | D | Conference abstract only. Study report does not mention the outcome of interest and protocol not available for assessment. |
| Elsner | Ilic 2016 | Y | Y | Y | A | Study report and protocol do not mention the outcome of interest. |
| Elsner | Jo 2008 | Y | N | NA | D | Study report does not mention the outcome of interest and protocol not available for assessment. |
| Elsner | Kang 2008 | Y | N | NA | D | Study report does not mention the outcome of interest and protocol not available for assessment. |
| Elsner | Kasashima 2012 | N | N | NA | D | Study report does not mention the outcome of interest and protocol not available for assessment. |
| Elsner | Kim 2009 | Y | N | NA | D | Study report does not mention the outcome of interest and protocol not available for assessment. |
| Elsner | Klomjai 2018 | Y | Y | Y | A | Study report and protocol do not mention the outcome of interest. |
| Elsner | Ko 2008 | Y | N | NA | D | Study report does not mention the outcome of interest and protocol not available for assessment. |
| Elsner | Kwon 2016 | N | N | NA | D | Study report does not mention the outcome of interest and protocol not available for assessment. |
| Elsner | Lindenberg 2010 | Y | Y | Y | A | Study report and protocol do not mention the outcome of interest. |
| Elsner | Madhavan 2011 | N | N | NA | D | Study report does not mention the outcome of interest and protocol not available for assessment. |
| Elsner | Mahmoudi 2011 | Y | N | NA | D | Study report does not mention the outcome of interest and protocol not available for assessment. |
| Elsner | Manji 2018 | Y | N | NA | D | Study report does not mention the outcome of interest and protocol not available for assessment. |
| Elsner | Mazzoleni 2019 | Y | Y | Y | A | Study report and protocol do not mention the outcome of interest. |
| Elsner | Montenegro 2016 | N | Y | Y | A | Study report and protocol do not mention the outcome of interest. |
| Elsner | Mortensen 2016 | Y | Y | Y | A | The authors assessed patients with the Stroke Impact Scale, using only the "hand function" items as a screening tool at baseline and not applying it afterwards (the "ADL" items, in particular). |
| Elsner | Nair 2011 | Y | N | NA | D | Study report does not mention the outcome of interest and protocol not available for assessment. |
| Elsner | Park 2013 | Y | N | NA | D | Study report does not mention the outcome of interest and protocol not available for assessment. |
| Elsner | Picelli 2015 | Y | N | NA | D | Study report does not mention the outcome of interest and protocol not available for assessment. |
| Elsner | Rabadi 2017 | Y | Y | Y | E | The outcome data have been reported as change scores and therefore not included in the meta-analysis with absolute values. |
| Elsner | Rossi 2013 | Y | N | NA | B | Trial report states that "the Barthel Index and the modified Rankin Scale were used as secondary outcome measures. These scales were assessed at onset, at 5 days after stroke and after 3 months.", but results at 3 months only have been reported as percentage of patients presenting a mRS score less than or equal to 2 and as mean and standard deviation for the BI. |
| Elsner | Saeys 2015 | Y | Y | Y | A | Study report and protocol do not mention the outcome of interest. |
| Elsner | Salazar 2019 | Y | Y | Y | A | Study report and protocol do not mention the outcome of interest. |
| Elsner | Sattler 2015 | Y | Y | N | B | The protocol states the outcome to be measured with the Barthel Index, yet the trial publication does not report it among the outcomes. |
| Elsner | Seo 2017 | Y | Y | N | B | The protocol states the outcome to be measured with the Modified Rankin Scale (mRS) "at baseline, 1 day and 4 weeks after treatment", but the manuscript does not include this data in the results. |
| Elsner | Shaheiwola 2018 | Y | Y | Y | A | Study report and protocol do not mention the outcome of interest. |
| Elsner | Sik 2015 | Y | N | NA | D | Study report does not mention the outcome of interest and protocol not available for assessment. |
| Elsner | Sohn 2013 | Y | N | NA | D | Study report does not mention the outcome of interest and protocol not available for assessment. |
| Elsner | Stagg 2012 | N | N | NA | D | Study report does not mention the outcome of interest and protocol not available for assessment. |
| Elsner | Sunwoo 2013 | Y | N | NA | D | Study report does not mention the outcome of interest and protocol not available for assessment. |
| Elsner | Tahtis 2012 | Y | N | NA | D | Study report does not mention the outcome of interest and protocol not available for assessment. |
| Elsner | Utarapichat 2018 | Y | N | NA | D | Study report does not mention the outcome of interest and protocol not available for assessment. |
| Elsner | Viana 2014 | Y | N | NA | D | Study report does not mention the outcome of interest and protocol not available for assessment. |
| Elsner | Wang 2014 | Y | N | NA | D | Study report does not mention the outcome of interest and protocol not available for assessment. |
| Elsner | Wong 2015 | Y | N | NA | D | Conference abstract only. Study report does not mention the outcome of interest and protocol not available for assessment. |
| Estévez-López | Haak 2008 | Y | N | NA | D | Study report does not mention the outcome of interest and protocol not available for assessment. |
| Estévez-López | Lynch 2012 | Y | Y | Y | C | Trial uses the FIQ scale, which is a 10-item validated scale that assesses physical functioning, pain, depression, anxiety, fatigue, morning tiredness, stiffness, job difficulty and overall well-being, but it does not report data for the subscales and, furthermore, uses graphs to report results. |
| Estévez-López | Sanudo 2015 | Y | N | NA | D | Study report does not mention the outcome of interest and protocol not available for assessment. |
| Fandim | Akhutina 2003 | Y | N | NA | D | Trial targeting spatial functioning, the upper limb is not involved in the intervention. |
| Fandim | Aran 2019 | Y | N | NA | D | Study report does not mention the outcome of interest and protocol not available for assessment. |
| Fandim | Arnori 2019 | Y | Y | Y | E | Trial targeting lower limbs and balance, the upper limb is not involved in the intervention. |
| Fandim | Atasavun 2016 | Y | N | NA | E | Trial targeting lower limbs and balance, the upper limb is not involved in the intervention. |
| Fandim | Chen 2012 | Y | N | NA | E | Trial targeting lower limbs, the upper limb is not involved in the intervention. |
| Fandim | Chen 2013 | Y | N | NA | E | Trial targeting lower limbs, the upper limb is not involved in the intervention. |
| Fandim | Cho 2016 | Y | N | NA | E | Trial targeting lower limbs, the upper limb is not involved in the intervention. |
| Fandim | Harris 2005 | Y | N | NA | D | Study report does not mention the outcome of interest and protocol not available for assessment. |
| Fandim | Jannink 2008 | Y | N | NA | C | The outcome has been transformed in a percentage score, thus preventing its possible inclusion in the meta-analysis. |
| Fandim | Klobucka 2013 | Y |  |  | G | Article in Slovak. |
| Fandim | Pin 2019 | Y | Y | Y | E | Trial targeting lower limbs and balance, the upper limb is not involved in the intervention. |
| Fandim | Pourazar 2018 | Y | N | NA | D | Study report does not mention the outcome of interest and protocol not available for assessment. |
| Fandim | Ren 2016 | Y |  |  | G | Article in Chinese. |
| Fandim | Tarakci 2016 | Y | N | NA | E | Trial targeting lower limbs and balance, the upper limb is not involved in the intervention. |
| Fernández López | Man 2006 | Y | N | NA | D | Study report does not mention the outcome of interest and protocol not available for assessment. |
| Fernández López | Yoo 2015 | Y | N | NA | F | "Digit span test" data were available in the trial report, but the review authors did not include them in meta-analysis. |
| Ferreira | Bower 2014 | Y | Y | Y | E | The outcome was measured in a different way (Step Test and Functional Reach Test) and therefore not included in meta-analysis. |
| Ferreira | Hung 2014 | Y | N | NA | E | The outcome was measured in a different way and therefore not included in meta-analysis, yet the outcome measure (Berg Balance Scale) was used at baseline for assessment. |
| Ferreira | McEwen 2014 | Y | Y | Y | E | The outcome was measured in a different way and therefore not included in meta-analysis, yet the outcome measure (Berg Balance Scale) was used at baseline for assessment. |
| Ferreira | Morone 2014 | Y | N | NA | C | The results have been reported graphically only. |
| Ferreira | Rajaratnam 2013 | Y | N | NA | E | Due to the not normal distribution of the data, mean and SD were not available and the outcome data were analyzed through non parametric tests. |
| Galeoto | Donkervoort 2001 | Y | N | NA | F | The Apraxia Test is based on a test of DeRenzi as well as those included in meta-analysis, and the results could have therefore included in meta-analysis as well. |
| Galeoto | Edmans 2000 | Y | N | NA | D | Study report does not mention the outcome of interest and protocol not available for assessment. |
| Galvão-Moreira | Andrade 2017 | N | Y | N | B | Trial protocol reporting the pain to be measured by the Visual Analogue Scale (VAS), but the outcome was not included in the trial report. |
| Galvão-Moreira | Gusi 2008 | N | Y | N | B | Trial protocol reporting the pain to be measured by the Visual Analogue Scale (VAS), but the outcome was not included in the trial report. |
| Galvão-Moreira | Letieri 2013 | N | N | NA | C | The results have been reported graphically only. |
| Galvão-Moreira | Mannerkorpi 2000 | Y | N | NA | E | The outcome was measured with a different scale (the FIQ Pain subscale) and therefore not included in meta-analysis. |
| Galvão-Moreira | Mannerkorpi 2009 | Y | N | NA | E | The outcome was measured with a different scale (the FIQ Pain subscale) and therefore not included in meta-analysis. |
| Galvão-Moreira | Munguía-Izquierdo 2008 | Y | N | NA | E | The outcome was measured in a different way (Pressure pain thresholds) and therefore not included in meta-analysis. |
| Galvão-Moreira | Tomas-Carus 2007 | Y | Y | Y | E | The outcome was measured with a different scale (SF-36 body pain subscale) and therefore not included in meta-analysis. |
| Galvão-Moreira | Tomas-Carus 2009 | N | Y | N | B | Trial protocol reporting the pain to be measured by the Visual Analogue Scale (VAS), but the outcome was not included in the trial report. |
| Galvão-Moreira | Zamuner 2015 | N | Y | Y | C | The results have been reported graphically only. |
| Gamble | Chung 2013 | Y | N | NA | D | Study report does not mention the outcome of interest and protocol not available for assessment. |
| Gamble | Dean 2007 | Y | N | NA | D | Study report does not mention the outcome of interest and protocol not available for assessment. |
| Gamble | Oh 2016 | Y | N | NA | D | Study report does not mention the outcome of interest and protocol not available for assessment. |
| García-Muñoz | Karami 2018 | Y | Y | N | B | Outcomes listed in the protocol: body balance, fatigue dimensions and fatigue severity; but the patients were evaluated by the Berg Balance Scale (BBS) to determine the existence of imbalance in these patients only before the intervention |
| Gates | Belchior 2007 | N | N | NA | D | Study report does not mention the outcome of interest and protocol not available for assessment. |
| Gates | Belchior 2008 | N |  |  | H | Thesis, not found and not possible to judge. |
| Gates | Belleville 2014 | N | N | NA | D | Study report does not mention the outcome of interest and protocol not available for assessment. |
| Gates | Cammarata 2011 | N |  |  | H | Conference abstract, not found and not possible to judge. |
| Gates | Casutt 2014 | N | N | NA | D | Study report does not mention the outcome of interest and protocol not available for assessment. |
| Gates | Combourieu 2014 | N | N | NA | D | Conference abstract. Study report does not mention the outcome of interest and protocol not available for assessment. |
| Gates | Desjardins-Crépeau 2016 | Y | N | NA | D | The outcome was measured at baseline. Study report does not mention the outcome of interest as a follow up measure and protocol not available for assessment. |
| Gates | Foerster 2009 | N | N | NA | D | Conference abstract. Study report mention the outcome for groups matching, but no other information has been provided, and protocol not available for assessment. |
| Gates | Forloni 2012 | N |  |  | H | Conference abstract, not found and not possible to judge. |
| Gates | Giovannini 2015 | N |  |  | H | Conference abstract, not found and not possible to judge. |
| Gates | Kim 2012 | N |  |  | H | Conference abstract, not found and not possible to judge. |
| Gates | Kim 2013a | N | N | NA | D | Conference abstract. Study report does not mention the outcome of interest and protocol not available for assessment. |
| Gates | Legault 2011 | Y | Y | Y | A | The outcome was measured at baseline. Study report and protocol do not mention the outcome of interest as a follow up measure. |
| Gates | Leung 2015 | Y | N | NA | D | Study report does not mention the outcome of interest and protocol not available for assessment. |
| Gates | Mombelli 2012 | N | N | NA | D | Conference abstract. Study report does not mention the outcome of interest and protocol not available for assessment. |
| Gates | NCT02462135 2014 | N | Y | NA | A | Registered protocol. It does not mention the outcome of interest. |
| Gates | NCT02512627 2015 | N | Y | NA | A | Registered protocol. It does not mention the outcome of interest. |
| Gates | Suo 2012 | N | N | NA | D | Conference abstract. Study report does not mention the outcome of interest and protocol not available for assessment. |
| Gates | Wild-Wall 2012 | N | N | NA | D | Study report does not mention the outcome of interest and protocol not available for assessment. |
| Gianola | Jansen 2013 | Y | Y | Y | C | The outcome was measured on multiple muscles and the score summed and not reported for each single muscle, thus preventing the study to be included in meta analysis. |
| Gianola | Kierkegaard 2011 | Y | N | NA | D | Study report does not mention the outcome of interest and protocol not available for assessment. |
| Gianola | Okkersen 2018 | Y | Y | Y | A | Study report and protocol do not mention the outcome of interest. |
| Grønfeldt | Sedghi 2017 | N | N | NA | C | Outcome data have been only reported as percentage change and p-value from pre- to post-test and no absolute value is available, thus preventing the study to be included in the meta-analysis. |
| Gutiérrez-Espinoza | Senbursa 2011 | Y | N | NA | C | "The Modified American Shoulder and Elbow Surgery (MASES) score was used in functional assessment". "The groups showed a significant difference in their MASES score at 4 weeks, while there was no difference at the 12 week follow-up (p>0.05) (Fig. 3)." The results have been reported graphically only. |
| Gutiérrez-Espinoza | Walther 2004 | Y | N | NA | C | "Shoulder function was assessed by use of the Constant-Murley score". "There was a significant improvement in the Constant-Murley score within 12 weeks in all three groups (P<.05); however, the improvement showed no difference among the three groups." The results have been reported graphically only. |
| Gutiérrez-Espinoza | Werner 2002 | Y | N | NA | G | Article in German. |
| Hall | Dijkstra‐Eshuis 2015 | Y | Y | NA | E | The outcome is available at a different timepoint (one year) and therefore not included in meta-analysis. |
| Hall | Laurenzio 2018 | Y | Y | NA | A | Study report and protocol do not mention the outcome of interest. |
| Hall | Laurienzo 2013 | Y | N | NA | D | Study report does not mention the outcome of interest and protocol not available for assessment. |
| Hall | Ocampo‐Trujillo 2014 | Y | N | NA | E | The outcome is available at a different timepoint and therefore not included in meta-analysis. |
| Han | Arrieta 2019 | Y | N | NA | D | Frailty status was measured with a slightly modified version of the Fried criteria only during the baseline assessment. Study report does not mention the outcome of interest as a follow up measure and protocol not available for assessment. |
| Han | Azad 2008 | Y | N | NA | D | Study report does not mention the outcome of interest and protocol not available for assessment. |
| Han | Blanc-Bisson 2008 | Y | N | NA | D | Study report does not mention the outcome of interest and protocol not available for assessment. |
| Han | Miller 2006 | Y | N | NA | D | Study report does not mention the outcome of interest and protocol not available for assessment. |
| Han | Milte 2016 | Y | N | NA | D | Study report does not mention the outcome of interest and protocol not available for assessment. Trial states that "A protocol for the design of the study has been published previously (17)" but the reference doesn't match. |
| Han | Rodriguez-Manas 2019 | Y | Y | Y | A | Study report and protocol do not mention the outcome of interest. |
| Han | Singh 2012 | Y | Y | Y | A | Study report and protocol do not mention the outcome of interest. |
| Han | Villareal 2011 | Y | Y | Y | E | The outcome has been measured but in a different way than the one used in the review (Frailty was assessed with the use of the modified Physical Performance Test, the measurement of VO2peak, and the Functional Status Questionnaire). |
| He | Cha 2015 | Y | N | NA | D | Study report does not mention the outcome of interest and protocol not available for assessment. |
| He | Fregni 2006 | Y | N | NA | D | Study report does not mention the outcome of interest and protocol not available for assessment. |
| He | Khedr 2009 | Y | N | NA | D | Study report does not mention the outcome of interest and protocol not available for assessment. |
| He | Khedr 2010 | Y | N | NA | D | Study report does not mention the outcome of interest and protocol not available for assessment. |
| He | Ludemann-Podubecka 2016 | Y | N | NA | D | Study report does not mention the outcome of interest and protocol not available for assessment. |
| Hislop | Callaghan 1995 | Y | N | NA | C | Data reported as median and range percentage change and therefore not included in meta-analysis. |
| Hislop | Özdinçler 2005 | Y |  |  | H |  |
| Hislop | Singh 2016 | Y | N | NA | C | The outcome measure is made of three subscales (one of which measured the outcome of interest), yet the authors reported the overall score only, thus preventing the study to be included in meta analysis. |
| Hopewell | Carpenter 1990 | Y | N | NA | D | Study report does not mention the outcome of interest and protocol not available for assessment. |
| Hopewell | Carter 1997 | Y |  |  | H |  |
| Hopewell | Ciaschini 2009 | Y | Y | Y | A | Study report and protocol do not mention the outcome of interest. |
| Hopewell | Close 1999 | Y | N | NA | D | Study report does not mention the outcome of interest and protocol not available for assessment. |
| Hopewell | Coleman 1999 | Y | N | NA | D | Study report does not mention the outcome of interest and protocol not available for assessment. |
| Hopewell | de Vries 2010 | Y | Y | Y | A | Study report and protocol do not mention the outcome of interest. |
| Hopewell | Fabacher 1994 | Y | N | NA | D | Study report does not mention the outcome of interest and protocol not available for assessment. |
| Hopewell | Jitapunkul 1998 | Y |  |  | H |  |
| Hopewell | Metzelthin 2013 | Y | Y | N | A | Study report and protocol do not mention the outcome of interest. |
| Hopewell | Newbury 2001 | Y | N | NA | D | Study report does not mention the outcome of interest and protocol not available for assessment. |
| Hopewell | Perula 2012 | Y | N | NA | D | Study report does not mention the outcome of interest and protocol not available for assessment. |
| Hopewell | Rubenstein 2007 | Y | N | NA | D | Study report does not mention the outcome of interest and protocol not available for assessment. |
| Hopewell | Russell 2010 | Y | N | NA | D | Study report does not mention the outcome of interest and protocol not available for assessment. |
| Hopewell | Shyu 2010 | Y | Y | Y | A | Study report and protocol do not mention the outcome of interest. |
| Hopewell | Spice 2009 | Y | N | NA | D | Study report does not mention the outcome of interest and protocol not available for assessment. |
| Hopewell | van Haastregt 2000 | Y | N | NA | D | Study report does not mention the outcome of interest and protocol not available for assessment. |
| Hopewell | van Rossum 1993 | Y | N | NA | D | Study report does not mention the outcome of interest and protocol not available for assessment. |
| Hopewell | Vetter 1992 | Y | N | NA | D | Study report does not mention the outcome of interest and protocol not available for assessment. |
| Hopewell | Wagner 1994 | Y | N | NA | D | Study report does not mention the outcome of interest and protocol not available for assessment. |
| Hopewell | Wyman 2005 | Y |  |  | H |  |
| Huang | Jeppesen 2016 | Y | Y | Y | C | Outcome data reported as mean percentage difference and not as absolute values. |
| Huang | Moretti 2009 | Y | Y | Y | B | Outcome data were reported only at the end of the intervention period, consisting of 20 weeks of standard care and 3 applications of shockwave therapy (approximately 10 days), even if the protocol states that "the outcomes were measured every 10 days". Moreover, the wound surface area was reported for patients with complete healing. |
| Huang | Nossair 2013 | Y | N | NA | D | Outcome data were reported only at the end of the whole intervention period, consisting of 12 weeks of standard care and 3 applications of shockwave therapy (3 weeks), and not at the end of the experimental intervention. |
| Huang | Tian 2013 | Y |  |  | H |  |
| Huang | Wang 2009 | Y | N | NA | B | Outcome data were reported at baseline only, even if the paper reports that "The size and depth of the ulcers [...] were carefully assessed before treatment and at different time intervals after treatment". |
| Huang | Wang 2011 | Y | Y | NA | A | Outcome data were planned to be measured and reported at baseline only, as stated in the paper "Pre-treatment evaluations included a complete history and physical examination [...]. The size, depth, and appearance of the ulcer were quantitatively assessed with physical examination and photo-documentation." |
| Imamura | Bestall 2003 | Y | N | NA | E | The outcome was measured with a different test (the shuttle walking test) and therefore not included in the meta-analysis. |
| Imamura | Linneberg 2012 | Y | Y | Y | E | The outcome was measured with a different test (the endurance shuttle walking time test) and therefore not included in the meta-analysis. |
| Jansen | Collins 2012 | Y | N | NA | F | Study repot includes the outcome data at the selected timepoint (12 weeks) even if the intervention lasts 24 weeks, but the review author did not include it in the meta-analysis at 12 weeks. |
| Jansen | Delaney 2014 | N | N | NA | D | Study report does not mention the outcome of interest and protocol not available for assessment. |
| Jansen | Delaney 2015 | Y | N | NA | D | Study report does not mention the outcome of interest and protocol not available for assessment. |
| Jansen | Jones 1996 | N |  |  | H |  |
| Jansen | McDermott 2009 | Y | N | NA | E | Study measures the outcome at 24 weeks, thus it was not included in the primary meta-analysis which consider the timepoint being 12 weeks. |
| Jansen | Nawaz 2001 | N | N | NA | D | Study report does not mention the outcome of interest and protocol not available for assessment. |
| Jansen | Sanderson 2006 | Y | N | NA | E | Study measures the outcome at 6 weeks, thus it was not included in the primary meta-analysis which consider the timepoint being 12 weeks. The outcome was reported graphically only. |
| Jansen | Saxton 2008 | N | N | NA | E | The study measures the outcome differently (maximum walking distance during an incremental shuttle-walk test) than planned by the systematic review (Maximal treadmill walking distance). |
| Jansen | Saxton 2011 | N | N | NA | E | The study measures the outcome differently (maximum walking distance during an incremental shuttle-walk test) than planned by the systematic review (Maximal treadmill walking distance). |
| Jansen | Treat-Jacobson 2011 | N | N | NA | E | Study report clearly states that they were not going to measure the outcome in the way planned by the systematic review. |
| Jansen | Walker 2000 | N | N | NA | E | The study measures the outcome differently (maximum walking distance during an incremental shuttle-walk test) than planned by the systematic review (Maximal treadmill walking distance). |
| Jaqueline da Cunha | Sharif 2017 | Y | N | NA | D | Study report does not mention the outcome of interest and protocol not available for assessment. |
| Kamonseki | Ajimsha 2011 | Y | N | NA | D | Study report does not mention the outcome of interest and protocol not available for assessment. |
| Kamonseki | Espí-López 2014b | Y | Y | Y | E | Same participants and data of Espí-López 2014a, already included in the meta-analysis. |
| Khattab | Chan 2017 | Y | N | NA | D | Study report does not mention the outcome of interest and protocol not available for assessment. |
| Khattab | Chan 2018 | Y | Y | Y | A | Study report and protocol do not mention the outcome of interest. |
| Khattab | Debreceni-Nagy 2019 | Y | N | NA | E | "Cognitive functions were evaluated by tests validated in the Hungarian practice including FIM cognitive subtest (FIM-cog, max. point 35) as a subjective measure; and Wechsler Adult Intelligence Scale-Fourth Edition (WAIS-IV) Working Memory (Digit Span) and Processing Speed subtests (Symbol Search and Coding; Wechsler, 2008) as objective measures". "There was no significant difference between the groups in either of the tests". Due to the non-normal distribution of the data, results were expressed by median and inter-quartile range. |
| Khattab | Kim 2017 | Y | N | NA | D | Study report does not mention the outcome of interest and protocol not available for assessment. |
| Khattab | Liu-Ambrose 2015 | Y | Y | N | E | "We used the verbal digits forward and backward tests to index working memory. Participants repeated progressively longer random number sequences in the same order as presented (forward) and the reversed order (backward). Successful performance on the verbal digits span backward test represents a measure of central executive function due to the additional requirement of manipulation of information within temporary storage (25). Thus, we subtracted the verbal digits backward test score from the verbal digits forward test score to provide an index of working memory with smaller difference scores indicating better performance." "At the end of the six-month intervention period, there were significant between-group differences in both selective attention and conflict resolution (p=0.02) and working memory (p=0.04)" Numerical data (mean and SD) for the difference between the Digit forward and the digit backward tests have been reported by the manuscript, but not included in the review as the review authors judged Verbal Digit Span Backward to be a measure of executive function. |
| Khattab | Mead 2007 | Y | N | NA | D | The trial was not included in any meta-analyses because mean and SDs are not available, but the outcome of interest was not mentioned by the study report and the protocol was not available for assessment. |
| Khattab | Nilsson 2001 | Y | N | NA | D | Study report does not mention the outcome of interest and protocol not available for assessment. |
| Khattab | Ploughman 2019 | Y | N | NA | D | Study report does not mention the outcome of interest and protocol not available for assessment. |
| Khattab | Quaney 2009 | Y | N | NA | E | Study report states "Trail-Making Task (A, B) assessed visual search ability, working memory, and attention switching" and reported the outcome as a difference between Trial-Making Task B and Trial-Making Task A. The review authors consider Trial Making Test A to be a measure of attention and processing speed and Trial Making Test B to be a measure of executive function, so it was not included in meta-analysis. |
| Khattab | Rabadi 2008 | Y | Y | Y | A | Study report and protocol do not mention the outcome of interest. |
| Kim | Avelaro 2010 | Y | Y | Y | C | The results (means only) have been reported graphically only. |
| Kim | Bergamin 2013 | Y | N | NA | D | Study report does not mention the outcome of interest and protocol not available for assessment. |
| Kim | Pérez de la Cruz 2017 | Y | Y | Y | A | Study report and protocol do not mention the outcome of interest. |
| Kim | Pérez de la Cruz 2018 | Y | N | NA | D | Study report does not mention the outcome of interest and protocol not available for assessment. |
| Kim | Simmons 1996 | Y | N | NA | D | Study report does not mention the outcome of interest and protocol not available for assessment. |
| Kim | Volpe 2014 | Y | N | NA | D | Study report does not mention the outcome of interest and protocol not available for assessment. |
| Kim | Volpe 2017 | Y | Y | Y | A | Study report and protocol do not mention the outcome of interest. |
| Kim | Zivi 2017 | Y | Y | Y | A | Study report and protocol do not mention the outcome of interest. |
| Laver | Cramer 2019 | Y | Y | Y | A | Study report and protocol do not mention the outcome of interest. |
| Laver | Forducey 2012 | Y | N | NA | C | Study report only states that "Significant pre-post differences were found for both the videophone and standard of care treatment conditions on the FIM and SF-12 (all ps < .05)", but no means and standard deviations have been reported. |
| Laver | Llorens 2015 | Y | N | NA | D | Study report does not mention the outcome of interest and protocol not available for assessment. |
| Laver | Meltzer 2018 | Y | N | NA | D | Study report does not mention the outcome of interest and protocol not available for assessment. |
| Laver | Piron 2008 | Y |  |  | H |  |
| Laver | Piron 2009 | Y | N | NA | D | Study report does not mention the outcome of interest and protocol not available for assessment. |
| Laver | Vauth 2016 | Y |  |  | G | Article in German. |
| Lee | Amieva 2016 | Y | N | NA | F | Trial uses a different scale from the ones used in the SR: depressive symptoms (Montgomery-Asberg Depression Rating Scale; MADRS) |
| Lee | Aslakson 2010 | Y | N | NA | D | Study report does not mention the outcome of interest and protocol not available for assessment. |
| Lee | Bakshy 2004 | Y |  |  | H | Doctoral dissertation partially available. |
| Lee | Hong & Choi 2011 | Y | N | NA | D | Study report does not mention the outcome of interest and protocol not available for assessment. |
| Lee | Hsu 2015 | Y | Y | Y | A | The NPI-NH is a semi-structured interview for use in nursing homes that collects data on residents’ neuro- psychiatric symptoms of dementia. The interview assesses 12 areas of behaviour and neuropsychiatric functioning: delusions, hallucinations, agitation, depression, anxiety, euphoria, apathy, disinhibition, irritability, aberrant motor behaviour, night‐time behaviour and ap- petite disturbance |
| Lee | Ito 2007 | Y | N | NA | D | Study report does not mention the outcome of interest and protocol not available for assessment. |
| Lee | Kallio 2018 | Y | Y | Y | A | the 15-dimensional instrument (15D), to assess HRQoL. The 15D correlates well with other HRQoL measures such as the Medical Outcomes Study 20-item Short-Form Survey, EuroQol-5D, and Nottingham Health Profile It is a standardized questionnaire consisting of 15 multiple-choice items to measure mobility, vision, hearing, breathing, sleeping, eating, speech, elimination, usual activities, mental function, discomfort and symptoms, depression, distress, vitality, and sexual activity |
| Lee | Lai 2004 | Y | N | NA | D |  |
| Lee | Lyu 2018 | Y | Y | Y | A | Study report does not mention the outcome of interest and protocol not available for assessment. |
| Lee | Maseda 2014 | Y | N | NA | D | Study report does not mention the outcome of interest and protocol not available for assessment. |
| Lee | Ridder 2013 | Y | N | NA | D |  |
| Lee | Sanchez 2016 | Y | N | NA | D | Study report does not mention the outcome of interest and protocol not available for assessment. |
| Lee | Shiltz 2018 | Y |  |  | H |  |
| Lee | Staal 2007 | Y | N | NA | D | Study report does not mention the outcome of interest and protocol not available for assessment. |
| Lee | van Der Ploeg 2013 | Y | Y | Y | A | Study report and protocol do not mention the outcome of interest. |
| Lee | Wang 2018 | Y | N | NA | D | Study report does not mention the outcome of interest and protocol not available for assessment. |
| Lee | Weise 2019 | Y | N | NA | D | Study report does not mention the outcome of interest and protocol not available for assessment. |
| Liao | Bruce-Brand 2012 | Y | Y | Y | A | Study report and protocol do not mention the outcome of interest. |
| Liao | Choi 2015 | Y | N | NA | D | Study report does not mention the outcome of interest and protocol not available for assessment. |
| Liao | Gür 2002 | Y | N | NA | D | Study report does not mention the outcome of interest and protocol not available for assessment. |
| Liao | Mahmoud 2017 | Y | N | NA | D | The outcome was planned to be measured and measured at baseline only. |
| Liao | Malas 2013 | Y | N | NA | D | Study report does not mention the outcome of interest and protocol not available for assessment. |
| Liao | Tsukagoshi 2014 | Y | N | NA | D | Study report does not mention the outcome of interest and protocol not available for assessment. |
| Liao | Valtonen 2011 | Y | Y | Y | A | Study report and protocol do not mention the outcome of interest. |
| Liao | Walls 2010 | Y | N | NA | D | Study report does not mention the outcome of interest and protocol not available for assessment. |
| Luo | Hornby 2016 | Y | N | NA | D | Study report does not mention the outcome of interest and protocol not available for assessment. |
| Luo | Lamberti 2017 | Y | N | NA | D | Study report does not mention the outcome of interest and protocol not available for assessment. |
| Martinez-Calderon | Bieler 2016 | Y | Y | Y | E | Trial report only states "When change in self-efficacy was measured with the Arthritis Self-efficacy Scale, there were no between-group differences at any time point except at 12 months, where improvements in the Pain and Function subscale were greater in the NW group compared with the ST group [pain: 11.1 points (95% CI: 0.1-22.2), P = 0.0471; function: 7.6 points (95% CI: 0.7–14.4), P = 0.0307]." and no absolute value has been reported, thus preventing the study to be included in the meta-analysis with post-treatment values. |
| Martinez-Calderon | Jones 2012 | Y | Y | N | E | Outcome data have been reported as mean change and 95% Confidence Interval only and no absolute value was available, thus preventing the study to be included in the meta-analysis. |
| Martinez-Calderon | Schachter 2003 | Y | N | NA | B | The outcome was planned to be measured at 8 and 16 weeks, yet only data at 16 weeks have been provided. |
| Mateo | Dimbwadyo-Terrer 2016 | Y | Y | Y | E | The outcome was measured with the upper limb part of the Motricity Index, which was probably not considered a measure of Hand-arm function by the review authors and therefore not included in the meta-analysis. |
| Mateo | DiPasquale-Lehnerz 1994 | Y | N | NA | C | No baseline data have been reported. The test consists of 7 subtests and only the means for each subtest have been reported, with no measure of variability nor total score. |
| Mateo | Hicks 2003 | Y | N | NA | D | Study report does not mention the outcome of interest and protocol not available for assessment. |
| Mateo | Klose 1993 | Y | N | NA | D | Study report does not mention the outcome of interest and protocol not available for assessment. |
| McGregor | Asbury 2012 | Y | N | NA | B | The outcome measure is listed among the outcomes in the methods section and clearly measured (some of the items of the scale have been discussed), yet data have been reported at baseline only. |
| McGregor | Belardinelli 2001 | Y | N | NA | E | The outcome was measured with two different questionnaires (Duke Activity Status Index and MOS short-form General Health Survey) and therefore not included in meta-analysis. |
| McGregor | Bettencourt 2005 | Y |  |  | H |  |
| McGregor | Briffa 2005 | Y | N | NA | E | Data reported at baseline for the whole population and not for the intervention and control group separately, but at follow up as mean change and 95% Confidence interval. |
| McGregor | Devi 2014 | Y | Y | Y | E | The outcome was measured with two different questionnaires (The MacNew questionnaire and The Seattle Angina Questionnaire (SAQ)) and therefore not included in meta-analysis. |
| McGregor | Hassan 2016 | Y | N | NA | D | The outcome was measured and the data were reported as means and standard deviations at follow up only (12 months) and not at the end of the intervention (6 months), but the protocol is not available for assessment. |
| McGregor | Hautala 2017 | Y | Y | Y | E | The outcome was measured with a different questionnaire (the 15D questionnaire) and therefore not included in meta-analysis. |
| McGregor | Højskov 2016 | Y | Y | Y | C | The outcome measure is made of eight subscales, yet the authors grouped them into two scales (mental health and physical health), thus preventing the study to be included in meta-analysis. |
| McGregor | Højskov 2019 | Y | Y | Y | C | The outcome measure is made of eight subscales, yet the authors grouped them into two scales (physical activity and mental health), thus preventing the study to be included in meta-analysis. |
| McGregor | Houle 2012 | Y | N | NA | E | The outcome was measured with a different questionnaire (The Quality of Life Index-cardiac version III) and therefore not included in meta-analysis. |
| McGregor | Mutwalli 2012 | Y | N | NA | C | The outcome measure is made of eight subscales, yet the authors reported the overall score only, thus preventing the study to be included in meta-analysis. |
| McGregor | Oerkild 2012 | Y | Y | Y | C | The outcome measure is made of eight subscales, yet the authors grouped them into two scales (physical component and mental component), thus preventing the study to be included in meta analysis. |
| McGregor | Peixoto 2015 | Y | N | NA | E | The outcome was measured with a different questionnaire (The MacNew Heart Disease HRQL questionnaire) and therefore not included in meta-analysis. |
| McGregor | Reid 2011 | Y | Y | Y | E | The outcome was measured with a different questionnaire (The 27-item MacNew instrument) and therefore not included in meta-analysis. |
| McGregor | Salvati 2016 | Y | N | NA | E | The outcome was measured with a different questionnaire (The Iranian version of the MacNew Heart Disease HRQoL questionnaire) and therefore not included in meta-analysis. |
| McGregor | Sandström 2005 | Y | N | NA | E | The outcome was measured with two different questionnaires (the EuroQol and Time Trade Off questionnaires) and therefore not included in meta-analysis. |
| McGregor | Santaularia 2016 | Y | Y | Y | E | The outcome was measured with a different questionnaire (the EuroQol5) and therefore not included in meta-analysis. |
| McGregor | Seki 2003 | N | N | NA | C | The results have been reported graphically only. |
| McGregor | West 2011 | Y | N | NA | D | The outcome was measured and the data were reported as means and standard deviations at follow up only (12 months) and not at the end of the intervention (6/8 weeks), but the protocol is not available for assessment. |
| McGregor | Zwisler 2008 | Y | Y | N | C | The outcome measure is made of eight subscales, yet the authors grouped them into two scales (physical component and mental component) and reported them at follow up (12 months) and not at the end of the intervention (6 weeks), thus preventing the study to be included in meta-analysis. |
| Mehrholz | Aprile 2017 | Y | N | NA | D | All participants were ambulatory at start of study. Study report does not mention the outcome of interest and protocol not available for assessment. |
| Mehrholz | Aprile 2019 | Y | N | NA | D | All participants were ambulatory at start of study. Study report does not mention the outcome of interest and protocol not available for assessment. Functional Ambulation Category (FAC) scores were reported as medians and interquartile ranges, and not as percentage/absolute number of patients achieving each score. |
| Mehrholz | Aschbacher 2006 | Y | N | NA | F | As reported in the systematic review, "No effect estimate (odds ratio (OR)) was feasible because no events (e.g., no participant reached the ability to walk) or only events (e.g., all participants regained walking) were reported", and the study was therefore included in the meta-analysis as "Non estimable", thus not contributing to the effect estimate. |
| Mehrholz | Bang 2016 | Y | N | NA | F | As reported in the systematic review, "No effect estimate (odds ratio (OR)) was feasible because no events (e.g., no participant reached the ability to walk) or only events (e.g., all participants regained walking) were reported", and the study was therefore included in the meta-analysis as "Non estimable", thus not contributing to the effect estimate. |
| Mehrholz | Belas Dos Santos 2018 | Y | Y | Y | A | Unclear if participants were ambulatory at start of study. Study report and protocol do not mention the outcome of interest. Functional Independence Measure (FIM) scores were reported as an overall score and not as an individual score for every item. |
| Mehrholz | Bergmann 2018 | Y | Y | Y | A | All participants were non-ambulatory at start of study. Study report and protocol do not mention the outcome of interest. Functional Ambulation Category (FAC) scores were reported as medians and interquartile ranges, as justified in the statistical analysis section of the methods ("Nonparametric statistics were used for all outcome variables because of the ordinal scale quality of the measures (SCP, BLS, POMA, FAC)"), but not as percentage/absolute number of patients achieving each score. |
| Mehrholz | Brincks 2011 | Y | N | NA | F | As reported in the systematic review, "No effect estimate (odds ratio (OR)) was feasible because no events (e.g., no participant reached the ability to walk) or only events (e.g., all participants regained walking) were reported", and the study was therefore included in the meta-analysis as "Non estimable", thus not contributing to the effect estimate. |
| Mehrholz | Buesing 2015 | Y | Y | NA | F | As reported in the systematic review, "No effect estimate (odds ratio (OR)) was feasible because no events (e.g., no participant reached the ability to walk) or only events (e.g., all participants regained walking) were reported", and the study was therefore included in the meta-analysis as "Non estimable", thus not contributing to the effect estimate. |
| Mehrholz | Calabrò 2018 | Y | Y | Y | A | All participants were not ambulatory at start of study. Study report and protocol do not mention the outcome of interest. Rivermead Mobility Index (RMI) scores were reported as overall score and graphically only. |
| Mehrholz | Cho 2015 | Y | N | NA | F | As reported in the systematic review, "No effect estimate (odds ratio (OR)) was feasible because no events (e.g., no participant reached the ability to walk) or only events (e.g., all participants regained walking) were reported", and the study was therefore included in the meta-analysis as "Non estimable", thus not contributing to the effect estimate. |
| Mehrholz | Dias 2006 | Y | N | NA | F | As reported in the systematic review, "No effect estimate (odds ratio (OR)) was feasible because no events (e.g., no participant reached the ability to walk) or only events (e.g., all participants regained walking) were reported", and the study was therefore included in the meta-analysis as "Non estimable", thus not contributing to the effect estimate. |
| Mehrholz | Erbil 2018 | Y | N | NA | D | All participants were ambulatory at start of study. Study report does not mention the outcome of interest at follow up and protocol not available for assessment. Functional Ambulation Category (FAC) scores were reported at baseline only. |
| Mehrholz | Forrester 2014 | Y | N | NA | F | As reported in the systematic review, "No effect estimate (odds ratio (OR)) was feasible because no events (e.g., no participant reached the ability to walk) or only events (e.g., all participants regained walking) were reported", and the study was therefore included in the meta-analysis as "Non estimable", thus not contributing to the effect estimate. |
| Mehrholz | Gandolfi 2019 | Y | Y | N | A | All participants were ambulatory at start of study. Study report and protocol do not mention the outcome of interest. The protocol states the Functional Ambulation Category (FAC) scores among the outcomes, but they were not reported in the manuscript. |
| Mehrholz | Geroin 2011 | Y | N | NA | F | As reported in the systematic review, "No effect estimate (odds ratio (OR)) was feasible because no events (e.g., no participant reached the ability to walk) or only events (e.g., all participants regained walking) were reported", and the study was therefore included in the meta-analysis as "Non estimable", thus not contributing to the effect estimate. |
| Mehrholz | Hidler 2009 | Y | N | NA | F | As reported in the systematic review, "No effect estimate (odds ratio (OR)) was feasible because no events (e.g., no participant reached the ability to walk) or only events (e.g., all participants regained walking) were reported", and the study was therefore included in the meta-analysis as "Non estimable", thus not contributing to the effect estimate. |
| Mehrholz | Hornby 2008 | Y | N | NA | F | As reported in the systematic review, "No effect estimate (odds ratio (OR)) was feasible because no events (e.g., no participant reached the ability to walk) or only events (e.g., all participants regained walking) were reported", and the study was therefore included in the meta-analysis as "Non estimable", thus not contributing to the effect estimate. |
| Mehrholz | Husemann 2007 | Y | N | NA | F | As reported in the systematic review, "No effect estimate (odds ratio (OR)) was feasible because no events (e.g., no participant reached the ability to walk) or only events (e.g., all participants regained walking) were reported", and the study was therefore included in the meta-analysis as "Non estimable", thus not contributing to the effect estimate. |
| Mehrholz | Jayaraman 2019 | Y | Y | Y | A | Ambulatory and non-ambulatory at start of study. Study report and protocol do not mention the outcome of interest. |
| Mehrholz | Kayabinar 2019 | Y | N | NA | D | All participants were ambulatory at start of study. Study report does not mention the outcome of interest and protocol not available for assessment. Rivermead Mobility Index (RMI) scores were reported as median, first and third quartile of the overall score only and not as percentage/absolute number of patients achieving each score. |
| Mehrholz | Kelley 2013 | Y | N | NA | D | All participants were ambulatory at start of study. Study report does not mention the outcome of interest and protocol not available for assessment. The Functional Independence Measure locomotion (FIM-L) score was listed among the secondary outcomes in the methods sections, but no results have been reported for that outcome. |
| Mehrholz | Kim 2015 | Y | Y | Y | F | As reported in the systematic review, "No effect estimate (odds ratio (OR)) was feasible because no events (e.g., no participant reached the ability to walk) or only events (e.g., all participants regained walking) were reported", and the study was therefore included in the meta-analysis as "Non estimable", thus not contributing to the effect estimate. |
| Mehrholz | Kim 2019a | Y | Y | Y | A | Ambulatory and not ambulatory at start of study. Study report and protocol do not mention the outcome of interest. Functional Ambulation Category (FAC) scores were reported both as means and standard deviations and as medians and interquartile ranges, but not as percentage/absolute number of patients achieving each score. |
| Mehrholz | Kim 2019b | Y | Y | Y | A | Ambulatory and not ambulatory at start of study. Study report and protocol do not mention the outcome of interest. Functional Ambulation Category (FAC) scores were reported as means and standard deviations, but not as percentage/absolute number of patients achieving each score. |
| Mehrholz | Kwon 2018 | Y | N | NA | D | All participants were non-ambulatory at start of study. Study report does not mention the outcome of interest and protocol not available for assessment. Functional Ambulation Category (FAC) scores were reported as means and standard deviations, but not as percentage/absolute number of patients achieving each score. |
| Mehrholz | Lee 2019 | Y | Y | N | A | All participants were ambulatory at start of study. Study report and protocol do not mention the outcome of interest. Functional Ambulation Category (FAC) scores were reported at baseline only despite being listed in the secondary outcomes in the registered protocol. |
| Mehrholz | Lu 2017 | Y |  |  | G | Unclear if participants were ambulatory at start of study. Article in Japanese. |
| Mehrholz | Nam 2019 | Y | Y | Y | A | All participants were non-ambulatory at start of study. Study report and protocol do not mention the outcome of interest. Functional Ambulation Category (FAC) scores were reported as means and standard deviations, but not as percentage/absolute number of patients achieving each score. Trial authors stated that "All values are presented as mean +/- SD. Although the primary outcome of FAC is scored on an ordinal scale as 1, 2, 3, 4, 5, and 6, we expressed the mean and SD because FAC is one of the most popular tools to measure the ambulatory function". |
| Mehrholz | Nam 2020 | Y | Y | Y | F | All participants were ambulatory at start of study. Functional Ambulation Category (FAC) scores were reported as percentage/absolute number of patients achieving each score as requested by the systematic review, yet the study was not included in meta-analysis. |
| Mehrholz | Noser 2012 | Y | Y | NA | F | As reported in the systematic review, "No effect estimate (odds ratio (OR)) was feasible because no events (e.g., no participant reached the ability to walk) or only events (e.g., all participants regained walking) were reported", and the study was therefore included in the meta-analysis as "Non estimable", thus not contributing to the effect estimate. |
| Mehrholz | Park 2018 | Y | N | NA | D | All participants were ambulatory at start of study. Study report does not mention the outcome of interest and protocol not available for assessment. Modified Barthel Index (mBI) scores were reported as means and standard deviations of the overall score only and not as percentage/absolute number of patients achieving each score to the scale items. |
| Mehrholz | Picelli 2016 | Y | N | NA | F | As reported in the systematic review, "No effect estimate (odds ratio (OR)) was feasible because no events (e.g., no participant reached the ability to walk) or only events (e.g., all participants regained walking) were reported", and the study was therefore included in the meta-analysis as "Non estimable", thus not contributing to the effect estimate. |
| Mehrholz | Sczesny-Kaiser 2019 | Y | Y | N | A | Ambulatory and non-ambulatory at start of study. Study report and protocol do not mention the outcome of interest. Functional Ambulation Category (FAC) scores were reported as means and standard deviations, but not as percentage/absolute number of patients achieving each score at post-treatment (at baseline only). |
| Mehrholz | Stein 2014 | Y | N | NA | F | As reported in the systematic review, "No effect estimate (odds ratio (OR)) was feasible because no events (e.g., no participant reached the ability to walk) or only events (e.g., all participants regained walking) were reported", and the study was therefore included in the meta-analysis as "Non estimable", thus not contributing to the effect estimate. |
| Mehrholz | Stolz 2019 | Y | N | NA | D | All participants were ambulatory at start of study. Study report does not mention the outcome of interest and protocol not available for assessment. Functional Independence Measure (FIM) scores were reported as medians and interquartile ranges for grouped scores (locomotion score, including locomotion and stairs) and not as an individual scores for every item. |
| Mehrholz | Tanaka 2012 | Y | N | NA | F | As reported in the systematic review, "No effect estimate (odds ratio (OR)) was feasible because no events (e.g., no participant reached the ability to walk) or only events (e.g., all participants regained walking) were reported", and the study was therefore included in the meta-analysis as "Non estimable", thus not contributing to the effect estimate. |
| Mehrholz | Tanaka 2019 | Y | N | NA | D | All participants were ambulatory at start of study. Study report does not mention the outcome of interest and protocol not available for assessment. |
| Mehrholz | Taveggia 2016 | Y | Y | N | A | All participants were ambulatory at start of study. Study report and protocol do not mention the outcome of interest. Functional Ambulation Category (FAC) scores were reported at baseline only despite being listed in the secondary outcomes in the registered protocol. |
| Mehrholz | Ucar 2014 | Y | N | NA | F | As reported in the systematic review, "No effect estimate (odds ratio (OR)) was feasible because no events (e.g., no participant reached the ability to walk) or only events (e.g., all participants regained walking) were reported", and the study was therefore included in the meta-analysis as "Non estimable", thus not contributing to the effect estimate. |
| Mehrholz | Waldman 2013 | Y | N | NA | F | As reported in the systematic review, "No effect estimate (odds ratio (OR)) was feasible because no events (e.g., no participant reached the ability to walk) or only events (e.g., all participants regained walking) were reported", and the study was therefore included in the meta-analysis as "Non estimable", thus not contributing to the effect estimate. |
| Mehrholz | Watanabe 2014 | Y | N | NA | F | As reported in the systematic review, "No effect estimate (odds ratio (OR)) was feasible because no events (e.g., no participant reached the ability to walk) or only events (e.g., all participants regained walking) were reported", and the study was therefore included in the meta-analysis as "Non estimable", thus not contributing to the effect estimate. |
| Mehrholz | Westlake 2009 | Y | N | NA | F | As reported in the systematic review, "No effect estimate (odds ratio (OR)) was feasible because no events (e.g., no participant reached the ability to walk) or only events (e.g., all participants regained walking) were reported", and the study was therefore included in the meta-analysis as "Non estimable", thus not contributing to the effect estimate. |
| Mehrholz | Yeung 2018 | Y | Y | Y | A | All participants were ambulatory at start of study. Study report and protocol do not mention the outcome of interest. Functional Ambulation Category (FAC) scores were reported as means and standard deviations at baseline and as mean change at post training and follow up. Trial authors stated that "More than half of the patients in Robotic Group showed improvements in gait independency and all reached FAC ≥ 5 post-training, while only one out of ten patients in Sham Group showed improved FAC post-training", so it was not possible to infer how many patients reached the cutoff of 5 points (as the scale was interpreted as 1 to 6 instead of 0 to 5) in the control group. |
| Mehrholz | Yun 2018 | Y | N | NA | D | All participants were non-ambulatory at start of study. Study report does not mention the outcome of interest and protocol not available for assessment. |
| Mendes | Burridge 1997 | N | N | NA | D | Study report does not mention the outcome of interest and protocol not available for assessment. |
| Mendes | Kluding 2013 | Y | Y | Y | B | Data at 12 weeks is not available in the report |
| Mendes | Kottink 2007 | Y | N | NA | C | The results have been reported graphically only. |
| Mendonça | Tauton 2003 | Y | N | NA | D | The outcome has been indirectly measured within the Victorian Institute of Sport Assessment-Patella score, which is a measure of function, and no separate assessment of pain has been conducted. No protocol is available for assessment. |
| Moucheboeuf | Belas Dos Santos 2018 | Y | Y | Y | A | Study report and protocol do not mention the outcome of interest. |
| Moucheboeuf | Chang 2012 | Y | N | NA | D | Study report does not mention the outcome of interest and protocol not available for assessment. |
| Moucheboeuf | Han 2016 | Y | N | NA | D | Study report does not mention the outcome of interest and protocol not available for assessment. |
| Moucheboeuf | Morone 2012 | Y | N | NA | D | Study report does not mention the outcome of interest and protocol not available for assessment. |
| Moucheboeuf | Morone 2018 | Y | N | NA | D | Study report does not mention the outcome of interest and protocol not available for assessment. |
| Muñoz-Vigueras | Baumgartner 2001 | Y | N | NA | D | Study report does not mention the outcome of interest and protocol not available for assessment. |
| Muñoz-Vigueras | Dumer 2014 | Y | N | NA | D | Study report does not mention the outcome of interest and protocol not available for assessment. |
| Muñoz-Vigueras | Johnson 1990 | Y | N | NA | E | The outcome was measured during different tasks (reading and monologue) and therefore not included in the meta-analysis for the sustained phonation subtask. |
| Muñoz-Vigueras | Levy 2020 | Y | Y | NA | A | Study report and protocol do not mention the outcome of interest. |
| Muñoz-Vigueras | Robertson 1984 | Y | N | NA | D | Study report does not mention the outcome of interest and protocol not available for assessment. |
| Muñoz-Vigueras | Sackley 2018 | Y | Y | Y | A | Study report and protocol do not mention the outcome of interest. |
| Muñoz-Vigueras | Saffarian 2019 | Y | N | NA | D | Study report does not mention the outcome of interest and protocol not available for assessment. |
| Muñoz-Vigueras | Sapir 2002 | Y | N | NA | D | Study report does not mention the outcome of interest and protocol not available for assessment. |
| Muñoz-Vigueras | Sapir 2007 | Y | N | NA | E | The outcome was measured during different tasks (phonation of vowels /i/, /u/, and /a/) and therefore not included in the meta-analysis for the sustained phonation subtask. |
| Nascimento_b | Jung 2014 | Y | N | NA | D | Study report does not mention the outcome of interest and protocol not available for assessment. |
| Nascimento_b | Lee 2010 | Y | N | NA | D | Study report does not mention the outcome of interest and protocol not available for assessment. |
| Nascimento_b | Lee 2018 | Y | N | NA | D | Study report does not mention the outcome of interest and protocol not available for assessment. |
| Nascimento_b | Noh 2008 | Y | N | NA | D | Study report does not mention the outcome of interest and protocol not available for assessment. |
| Nascimento_b | Paizan 2009 | N | N | NA | D | Study report does not mention the outcome of interest and protocol not available for assessment. |
| Nascimento_b | Tripp 2014 | Y | N | NA | D | Study report does not mention the outcome of interest and protocol not available for assessment. |
| Nascimento_b | Zhang 2016 | Y | N | NA | D | Study report does not mention the outcome of interest and protocol not available for assessment. |
| Navarro-Santana | Zegarra-Parodi 2016 | Y | N | NA | C | the outcome is present but data are not available. |
| Nayak | Saleh 2019 | Y | Y | Y | E | The outcome was measured with a different scale (the dynamic balance indices using the Biodex Balance System) and therefore not included in meta-analysis. |
| Nayak | Zangh 2016 | Y | N | NA | D | Study report does not mention the outcome of interest and protocol not available for assessment. |
| Orgeta | Costa 2014 | Y | N | NA | D | Study report does not mention the outcome of interest and protocol not available for assessment. |
| Pan | Katz 2013 | Y | Y | Y | E | The outcome was measured with a different scale (the pain score of the Knee Injury and Osteoarthritis Outcome Scale (KOOS)) and therefore not included in meta-analysis. |
| Parmenter | Hiatt 1994 | Y | N | NA | F | Trial states that "Subjects were tested on both a graded and constant-load treadmill protocol (conducted on separate days) on entry and after 12 and 24 weeks" and changes in both protocols have been reported (Table 3 and table 5), yet only data on graded-load treadmill protocol have been used by the review authors. |
| Parmenter | Holm 1973 | Y |  |  | H |  |
| Parmenter | McDermott 2009 | Y | Y | N | E | Only the progressive grade treadmill protocol has been used in the trial and data have been therefore not included in the meta-analysis on constant grade treadmill protocol. |
| Parmenter | McGuigan 2001 | Y | N | NA | E | Only the progressive grade treadmill protocol has been used in the trial and data have been therefore not included in the meta-analysis on constant grade treadmill protocol. |
| Parmenter | Parr 2009 | Y | N | NA | E | Only the progressive grade treadmill protocol has been used in the trial and data have been therefore not included in the meta-analysis on constant grade treadmill protocol. |
| Pazzianotto-Forti | Baetge 2017 | Y | N | NA | D | Study report does not mention the outcome of interest and protocol not available for assessment. |
| Pazzianotto-Forti | Baillot 2016 | Y | Y | Y | A | Study report and protocol do not mention the outcome of interest. |
| Pazzianotto-Forti | Carroll 2012 | Y | N | NA | D | Study report does not mention the outcome of interest and protocol not available for assessment. |
| Pazzianotto-Forti | Castello-Simões 2013 | Y | N | NA | D | Study report does not mention the outcome of interest and protocol not available for assessment. |
| Pazzianotto-Forti | Daniels 2017 | Y | N | NA | D | Study report does not mention the outcome of interest and protocol not available for assessment. |
| Pazzianotto-Forti | Frimel 2008 | Y | N | NA | D | Study report does not mention the outcome of interest and protocol not available for assessment. |
| Pazzianotto-Forti | Hassannejad 2017 | Y | Y | Y | A | Study report and protocol do not mention the outcome of interest. The trial authors measured the distance covered during the Twelve-Minute Walk-Run Test, which cannot be considered as a measure of walking speed only if transformed. |
| Pazzianotto-Forti | Herring 2014 | Y | N | NA | D | Study report does not mention the outcome of interest and protocol not available for assessment. |
| Pazzianotto-Forti | Herring 2017 | Y | N | NA | D | Study report does not mention the outcome of interest and protocol not available for assessment. |
| Pazzianotto-Forti | Labrunée 2012 | Y | N | NA | D | Study report does not mention the outcome of interest and protocol not available for assessment. |
| Pazzianotto-Forti | Manini 2010 | Y | Y | Y | C | The outcome data have been reported graphically and as adjusted difference and p-value only, and were therefore not included in the meta-analysis. |
| Pazzianotto-Forti | Marcon 2016 | Y | Y | Y | A | Study report and protocol do not mention the outcome of interest. |
| Pazzianotto-Forti | Ozcelik 2015 | Y | N | NA | D | Study report does not mention the outcome of interest and protocol not available for assessment. |
| Pazzianotto-Forti | Plotnikoff 2010 | Y | Y | Y | A | Study report and protocol do not mention the outcome of interest. |
| Pazzianotto-Forti | Scanga 1998 | Y | N | NA | D | Study report does not mention the outcome of interest and protocol not available for assessment. |
| Pazzianotto-Forti | Shah 2011 | Y | N | NA | D | Study report does not mention the outcome of interest and protocol not available for assessment. |
| Pazzianotto-Forti | Shah 2011 | Y | Y | Y | A | Study report and protocol do not mention the outcome of interest. |
| Pazzianotto-Forti | Snel 2012 | Y | N | NA | D | Study report does not mention the outcome of interest and protocol not available for assessment. |
| Pazzianotto-Forti | Svensson 2017 | Y | N | NA | D | Study report does not mention the outcome of interest and protocol not available for assessment. |
| Pazzianotto-Forti | Thomson 2016 | Y | Y | Y | A | Study report and protocol do not mention the outcome of interest. |
| Pazzianotto-Forti | Villareal 2011 | Y | Y | N | E | Outcome data have been reported as mean change and standard deviation, and were therefore not included in meta-analysis. |
| Pazzianotto-Forti | Villareal 2017 | Y | Y | Y | E | Outcome data have been reported as mean change and standard deviation, and were therefore not included in meta-analysis. |
| Pazzianotto-Forti | Woodlief 2015 | Y | N | NA | D | Study report does not mention the outcome of interest and protocol not available for assessment. |
| Pazzianotto-Forti | Wycherley 2010 | Y | N | NA | D | Study report does not mention the outcome of interest and protocol not available for assessment. |
| Pogrebnoy | Langhammer 2008 | Y | N | NA | E | The outcome was planned but measured in a different way than the one considered by the SR. |
| Pogrebnoy | Marzolini 2018 | Y | N | NA | E | The outcome was planned but measured in a different way than the one considered by the SR. |
| Pogrebnoy | Pang 2005 | Y | N | NA | C | They use a scale which the others trials use to assess speed velocity but they don't cite speed velocity: the 6-minute walk test (6MWT) was used to assess mobility. The distance walked in 6 minutes was recorded. The 6MWT has been shown to be a reliable method of assessing walking performance in individuals with stroke. |
| Pozuelo-Carrascosa | Britto 2011 | Y | Y | Y | A | Study report and protocol do not mention the outcome of interest. |
| Pozuelo-Carrascosa | Cho 2018 | Y | Y | Y | A | Study report and protocol do not mention the outcome of interest. |
| Pozuelo-Carrascosa | Guillen-Sola 2017 | Y | Y | Y | A | Study report and protocol do not mention the outcome of interest. |
| Pozuelo-Carrascosa | Jung 2015 | N | N | NA | D | Study report does not mention the outcome of interest and protocol not available for assessment. |
| Pozuelo-Carrascosa | Jung 2017 | Y | N | NA | D | Study report does not mention the outcome of interest and protocol not available for assessment. |
| Pozuelo-Carrascosa | Kulnik 2015 | Y | Y | N | B | Study protocol mentions the outcome to be measured "at baseline, completion of training (4 weeks) and at 3 months to assess sustainability of effect", but study report does not mention it among the outcomes and reports it only at baseline. |
| Pozuelo-Carrascosa | Lee 2018 | Y | N | NA | D | Study report does not mention the outcome of interest and protocol not available for assessment. |
| Pozuelo-Carrascosa | Menezes 2019 | Y | Y | Y | A | Study report and protocol do not mention the outcome of interest. |
| Pozuelo-Carrascosa | Messagi-Sartor 2015 | Y | Y | Y | A | Study report and protocol do not mention the outcome of interest. |
| Roberts | Bower 2012 | N | N | NA | D | Study report does not mention the outcome of interest and protocol not available for assessment. |
| Roberts | Desbiens 2017 | N | N | NA | D | Study report does not mention the outcome of interest and protocol not available for assessment. |
| Roberts | Goodwin 2014 | N | N | NA | D | Study report does not mention the outcome of interest and protocol not available for assessment. |
| Roberts | Harrigan 2016 | N | N | NA | D | Study report does not mention the outcome of interest and protocol not available for assessment. |
| Roberts | Kiecolt‐Glaser 2014 | N | N | NA | D | Study report does not mention the outcome of interest and protocol not available for assessment. |
| Roberts | Ligibel 2011 | N | N | NA | D | Study report does not mention the outcome of interest and protocol not available for assessment. |
| Roberts | Lohrisch 2011 | Y | N | NA | C | Conference abstract only. Trial report only states that "A 48 week supervised exercise intervention did not have a measurable improvement in related A/M symptoms as measured by 12W SF36 bodily pain scores", but did not report numerical values. |
| Roberts | Paulo 2019 | N | Y | Y | A | Study report and protocol do not mention the outcome of interest. |
| Roberts | Payne 2008 | N | N | NA | D | Study report does not mention the outcome of interest and protocol not available for assessment. |
| Roberts | Reeves 2017 | N | N | NA | D | Study report does not mention the outcome of interest and protocol not available for assessment. |
| Roberts | Rogers 2009 | N | N | NA | F | Joint pain data are available at baseline and post intervention for the experimental and control groups as means and standard deviations, yet not included in the systematic review because of "Wrong outcomes. Baseline AIMSS not investigated". |
| Roberts | Segal 2011 | N | N | NA | D | Conference abstract only. Study report does not mention the outcome of interest and protocol not available for assessment. |
| Roberts | Varadarajan 2016 | Y | N | NA | C | Conference abstract only. Trial report only states that "No significant differences were observed in pain scale (PS) and pain disability index (PDI)", but did not report numerical values. |
| Robson | Toda 1998 | Y |  |  | H |  |
| Robson | Wolf 2010 | Y | Y | Y | A | The trial used the WOMAC scale, yet planned to use it to assess only function (as stated in the protocol: "We used the Function Scale only for this study"). The outcome of interest was therefore not measured. |
| Rueda | Goswami 2019 | Y | Y | Y | C | The outcome was planned and reported in the trial reports with the same scale, but not in a way that allowed its inclusion in the meta-analysis (data are available only in %). |
| Shahabi | DeMeyer 2015 | N | N | NA | D | Study report does not mention the outcome of interest and protocol not available for assessment. |
| Shahabi | Lee 2014 | N | N | NA | D | Study report does not mention the outcome of interest and protocol not available for assessment. |
| Shahabi | Nikamp 2019 | Y | Y | N | E | Secondary analysis of a previous study (Nikamp 2017) already included in meta-analysis, which reported the outcome of interest. |
| Silva | Braun 2012 | Y | N | NA | C | Trial states to use the 10MWT as a measure of walking speed, but reports it as seconds and not as meters/seconds, thus preventing it to be included in meta-analysis. |
| Silva | Cho 2012 | Y | N | NA | C | Trial uses the 10MWT, but reports it as seconds to walk 10 meters and not as walking speed. |
| Silva | Dickstein 2014 | Y | N | NA | C | Cross-over trial. The outcome of interest has been included as seconds taken for walking 10 meters only and not as walking speed at the end of the full trial and not at the end of the first phase. |
| Silva | Kim 2013a | Y | N | NA | F | Gait speed reported as cm/s. |
| Silva | Kim 2013b | N | N | NA | D | Study report does not mention the outcome of interest and protocol not available for assessment. |
| Silva | Kumar 2013a | Y |  |  | H |  |
| Silva | Kumar 2013b | N | N | NA | D | The Functional Gait Assessment is a 10-item test that contains 7 of the 8 items (except walking around obstacles) from the Dynamic Gait Index and 3 additional tasks, including walking with a narrow base of support, walking with the eyes closed, and ambulating backward. Walking speed has not been assessed. |
| Silva | Lee 2010 | Y |  |  | H |  |
| Silva | Lee 2015 | Y | N | NA | D | Study report does not mention the outcome of interest and protocol not available for assessment. |
| Silva | Liu 2004 | Y | N | NA | D | Study report does not mention the outcome of interest and protocol not available for assessment. |
| Silva | Liu 2009 | Y | N | NA | D | Study report does not mention the outcome of interest and protocol not available for assessment. |
| Silva | Malouin 2004 | N | N | NA | D | Study report does not mention the outcome of interest and protocol not available for assessment. |
| Silva | Malouin 2009 | N | N | NA | D | Study report does not mention the outcome of interest at follow up and protocol not available for assessment. The outcome is reported at baseline only ("A clinical assessment was conducted at baseline"). |
| Silva | Mihara 2012 | N | N | NA | D | Study report does not mention the outcome of interest and protocol not available for assessment. |
| Silva | Page 2005 | N | N | NA | D | Study report does not mention the outcome of interest and protocol not available for assessment. |
| Silva | Park 2019 | Y | Y | Y | A | Study report and protocol do not mention the outcome of interest. |
| Silva | Saito 2013 | N | N | NA | D | Study report does not mention the outcome of interest and protocol not available for assessment. |
| Silva | Schuster 2009 | N | Y | Y | A | Study report and protocol do not mention the outcome of interest. |
| Silva | Schuster 2012 | Y | Y | Y | A | Study report and protocol do not mention the outcome of interest. |
| Silva | Suvadeep 2017 | Y | N | NA | C | The outcome of interest has been assessed as seconds taken for walking 10 meters, but the results have been reported as post-intervention means for the two groups, t-value and p-value. |
| Silva | Yan 2013 | Y |  |  | G |  |
| Silva | Zhang 2013 | Y |  |  | H |  |
| Silva | Zhu 2017 | Y |  |  | G | Chinese full text. |
| Su | Antypas 2014 | Y | Y | Y | E | The main outcome measure was self-reported overall physical activity measured with the International Physical Activity Questionnaire (IPAQ). Data have been reported as median and IQR given that normal distribution was not assumed. |
| Su | Dale 2015 | Y | Y | Y | A | Study report and protocol do not mention the outcome of interest. |
| Su | Frederix 2015 | Y | N | NA | D | Study report does not mention the outcome of interest and protocol not available for assessment. |
| Su | Johnston 2016 | Y | Y | Y | C | Data are presented as "median change in exercise minutes per week". |
| Su | Lear 2014 | Y | Y | Y | C | Data are reported as median and interquartile ranges. |
| Su | Lindsay 2009 | Y | N | NA | E | Exercise frequency was measured in terms of ‘how many days during a typical week you spend in moderate exercise’ where a higher score indicates more time spent in exercise. The systematic review would have required the data reported as minutes/week. |
| Su | Maddison 2015 | Y | Y | Y | C | Data are presented with mean with no measure of variability (standard deviation). |
| Su | Southard 2003 | Y | N | NA | C | Data is reported without SD |
| Su | Varnfield 2014 | Y | Y | Y | A | Study report and protocol do not mention the outcome of interest. |
| Su | Vernooij 2012 | Y | Y | Y | A | Study report and protocol do not mention the outcome of interest. |
| Surace | Cosentino 2003 | Y | N | NA | B | The outcome has been measured with the Constant and Murley pain subscale, yet only the mean value (and no standard deviation) for the experimental group, but no data for the control group, have been reported. |
| Surace | Gerdesmeyer 2003 | Y | N | NA | D | Study report does not mention the outcome of interest at the selected timepoint and protocol not available for assessment. |
| Surace | Hearnden 2009 | Y | N | NA | B | The outcome has been measured with the Visual Analogue Scale according to the methods section "at the end of 1st week, 6 weeks and 6 months", yet the data have been reported graphically only (with no standard deviation) for the experimental group, but no data have been reported for the control group. |
| Surace | Kolk 2013 | Y | N | NA | D | Study report does not mention the outcome of interest at the selected timepoint and protocol not available for assessment. |
| Surace | Kvalvaag 2017 | Y | Y | Y | B | The protocol states that the outcome should have been measured at 6 weekds ("Pain and function measured on a 11 point Likert type scale [ Time Frame: 6 weeks, 12 weeks, 24 weeks and 1 year]"), but results have been reported at 12 and 24 weeks only. |
| Surace | Peters 2004 | Y | N | NA | D | The outcome has been assessed only during the interventions and not before and after it, and the protocol is not available for assessment. |
| Takahashi | Cameron 2011 | Y | N | NA | C | Trial states that "One participant died during the 4 months", but did not specify in which group he was allocated. |
| Takahashi | Ekinci 2016 | Y | N | NA | D | Study report does not mention the outcome of interest and protocol not available for assessment. |
| Takahashi | Flodin 2015 | Y | N | NA | D | Study report does not mention the outcome of interest and protocol not available for assessment. |
| Takahashi | Hirooka 2017 | Y | N | NA | H |  |
| Takahashi | Malafarina 2017 | Y | Y | Y | C | Trial states "During admission 6 patients died (5.6%)", but did not specify in which group they were allocated. |
| Takahashi | Milte 2016 | Y | N | NA | D | Study report does not mention the outcome of interest and protocol not available for assessment. |
| Tomazoni | Soriano 2008 | Y | N | NA | C | The outcome has been transformed in a categorical variable, yet being measured as a continuous variable, thus preventing it to be included in meta-analysis. |
| Tomazoni | Toya 2004 | Y | N | NA | C | The outcome has been transformed in a categorical variable, yet being measured as a continuous variable, thus preventing it to be included in meta-analysis. |
| van Nispen | Bradley 2005 | Y | N | NA | B | Trial methods state that the patients have been assessed with two items of the Macular Disease Dependent Quality of Life (MacDQoL) questionnaire, but no results have been provided except for no significant differences ("With this small sample, no significant findings were anticipated from the questionnaire data"). |
| van Nispen | Brody 1999 | Y | N | NA | D | Study report does not mention the outcome of interest and protocol not available for assessment. |
| van Nispen | Kaluza 1996 | Y | N | NA | D | Study report does not mention the outcome of interest and protocol not available for assessment. |
| van Nispen | Kamga 2017 | Y | Y | Y | A | Study report and protocol do not mention the outcome of interest. |
| van Nispen | Mozaffar Jalali 2014 | Y | N | NA | D | Study report does not mention the outcome of interest and protocol not available for assessment. |
| van Nispen | Rumrill 1999 | Y |  |  | H |  |
| Waldauf | Dong 2016 | Y | N | NA | D | Study report does not mention the outcome of interest and protocol not available for assessment. |
| Waldauf | Gerovasili 2009 | Y | Y | Y | A | Outcome measured at baseline. No reference to the outcome measure in the trial nor in the protocol as a follow up measure. |
| Waldauf | Goll 2015 | Y | N | NA | D | conference abstract only |
| Waldauf | Shaolin 2019 | Y |  |  | H | The reference is not available in the review's bibliography |
| Waldauf | Yosef-Brauner 2015 | Y | N | NA | D | Study report does not mention the outcome of interest and protocol not available for assessment. |
| Wang | Aline 2018 | Y | N | NA | H | Reference of the study is not reported in the SR. |
| Wang | Brummeel 2014 | Y | Y | Y | A | Study report and protocol do not mention the outcome of interest. |
| Wang | Camilla 2012 | Y | N | NA | H | Reference of the study is not reported in the SR. |
| Wang | Chen 2012 | Y | N | NA | H | Reference of the study is not reported in the SR. |
| Wang | Chen 2015 | Y | N | NA | H | Reference of the study is not reported in the SR. |
| Wang | Dong 2014 | Y | N | NA | H | Reference of the study is not reported in the SR. |
| Wang | Dong 2016 | Y | N | NA | D | Study report does not mention the outcome of interest and protocol not available for assessment. |
| Wang | Feng 2018 | Y | N | NA | H | Reference of the study is not reported in the SR. |
| Wang | Guo 2016 | Y | N | NA | H | Reference of the study is not reported in the SR. |
| Wang | Huang 2016 | Y | N | NA | H | Reference of the study is not reported in the SR. |
| Wang | Jiang 2017 | Y | N | NA | H |  |
| Wang | Kayambu 2016 | Y | Y | Y | A | Study report and protocol do not mention the outcome of interest. |
| Wang | Kho 2019 | Y | Y | Y | A | Study report and protocol do not mention the outcome of interest. |
| Wang | Ma 2018 | Y | N | NA | H | Reference of the study is not reported in the SR. |
| Wang | McWilliams 2018 | Y | Y | Y | E | Trial used the Medical Research Council (MRC) sum score and hand grip strength as a measure of ICU-acquired weakness. |
| Wang | Morris 2016 | Y | Y | Y | A | Study report and protocol do not mention the outcome of interest. |
| Wang | Moss 2015 | Y | N | NA | H | Reference of the study is not reported in the SR. |
| Wang | Nidahl 2019 | Y | Y | Y | A | Study report and protocol do not mention the outcome of interest. |
| Wang | Patman 2001 | Y | N | NA | D | Study report does not mention the outcome of interest and protocol not available for assessment. |
| Wang | Quan 2016 | Y | N | NA | H | Reference of the study is not reported in the SR. |
| Wang | Rao 2017 | Y | N | NA | H |  |
| Wang | Shao 2015 | Y | N | NA | H | Reference of the study is not reported in the SR. |
| Wang | Sun 2017 | Y | N | NA | H | Reference of the study is not reported in the SR. |
| Wang | Templeton 2007 | Y | N | NA | H | Reference of the study is not reported in the SR. |
| Wang | Wu 2017 | Y | N | NA | H |  |
| Wang | Yu 2016 | Y | N | NA | H |  |
| Wang | Zeng F 2017 | Y | N | NA | H | Reference of the study is not reported in the SR. |
| Wang | Zeng H 2017 | Y | N | NA | H | Reference of the study is not reported in the SR. |
| Wang | Zhang A 2017 | Y | N | NA | H | Reference of the study is not reported in the SR. |
| Wang | Zhang Y 2013 | Y | N | NA | H | Reference of the study is not reported in the SR. |
| Wang | Zhu Y 2016 | Y | N | NA | H | Reference of the study is not reported in the SR. |
| Wilhelm | Ris 2016 | Y | Y | Y | A | Study report and protocol do not mention a measure of the outcome of interest. |
| Woodley | Agur 2005 | N |  |  | H | Conference abstract, not found and not possible to judge. |
| Woodley | Barakat 2011 | Y | N | NA | D | The outcome has been reported at a different timepoint [early postnatal period (0-3 months)]. |
| Woodley | Barakat 2015 | N | Y | Y | A | Study report and protocol do not mention the outcome of interest. |
| Woodley | Barakat 2016 | N | Y | Y | A | Study report and protocol do not mention the outcome of interest. |
| Woodley | Barakat 2018 | N | Y | Y | A | Study report and protocol do not mention the outcome of interest. |
| Woodley | Brik 2019 | N | Y | Y | A | Study report and protocol do not mention the outcome of interest. |
| Woodley | Dias 2011 | N | Y | Y | A | Study report and protocol do not mention the outcome of interest. |
| Woodley | Dias 2018 | N | N | Y | D | Study report does not mention the outcome of interest and protocol not available for assessment. |
| Woodley | Dieb 2017 | N | Y | NA | A | Study report not found and protocol does not mention the outcome of interest |
| Woodley | Domingues 2015 | N | Y | NA | A | Study protocol only. It does not mention the outcome of interest. |
| Woodley | El-Shamy 2018 | N | N | NA | D | Study report does not mention the outcome of interest and protocol not available for assessment. |
| Woodley | Gaier 2010 | Y | N | NA | H | Conference abstract, not found and not possible to judge. |
| Woodley | Huang 2014 | N |  |  | H |  |
| Woodley | Leon-Larios 2017 | N | N | NA | D | Study report does not mention the outcome of interest and protocol not available for assessment. |
| Woodley | Li 2010 | N |  |  | H |  |
| Woodley | Liu 2013 | N |  |  | H |  |
| Woodley | Nielsen 1988 | N | N | NA | D | Study report does not mention the outcome of interest and protocol not available for assessment. |
| Woodley | Reilly 2002 | Y | N | NA | E | The outcome has been reported at different timepoints [mid-postnatal period (> 3-6 months) and long term (> 5 years)]. |
| Woodley | Ruiz 2013 | N | Y | Y | A | Study report and protocol do not mention the outcome of interest. |
| Woodley | Santos-Rocha 2015 | N |  |  | H |  |
| Woodley | Siva 2014 | N |  |  | H |  |
| Woodley | Stothers 2002 | Y | N | NA | H | Conference abstract, not found and not possible to judge. |
| Woodley | Wang 2014 | N | N | NA | D | Study report does not mention the outcome of interest and protocol not available for assessment. |
| Xie | Chen 2014 | Y |  |  | G | Article is available in Chinese. |
| Xie | Cui 2016 | Y |  |  | G | Article is available in Chinese. |
| Xie | Ge 2011 | Y |  |  | G | Article is available in Chinese. |
| Xie | Guo 2017 | Y |  |  | G | Article is available in Chinese. |
| Xie | Hao 2018 | Y |  |  | G | Article is available in Chinese. |
| Xie | Lao 2013 | Y |  |  | G | Article is available in Chinese. |
| Xie | Liu 2012 | Y |  |  | G | Article is available in Chinese. |
| Xie | Luo 2017 | Y |  |  | G | Article is available in Chinese. |
| Xie | Qian 2012 | Y |  |  | G | Article is available in Chinese. |
| Xie | Sun 2014 | Y |  |  | G | Article is available in Chinese. |
| Xie | Zhang 2018 | Y |  |  | G | Article is available in Chinese. |
| Xu | Dulfer 2014 | Y | Y | Y | A | Study report does not mention the outcome of interest and protocol not available for assessment. |
| Xu | Therrien 2003 | Y |  |  | H |  |
| Xu | Westhoff-Bleck 2013 | Y | Y | Y | F | The outcome was measured and reported in the trial, yet it was not included in the meta-analysis. |
| Xu | Winter 2011 | Y | Y | Y | E | Outcome data reported as median and range being no symmetrically distributed. |
| Yan | Chen 2019 | Y |  |  | H |  |
| Yan | Hosseinzadeh 2018 | Y | N | NA | D | Study report does not mention the outcome of interest and protocol not available for assessment. |
| Yan | Park 2013 | Y | N | NA | C | Outcome data have been reported as post/pre ratio of scores and not as absolute values. |
| Yan | Shaker 2018 | Y | N | NA | D | The outcome measure was used as an inclusion criterion, as stated in the paper "Included in this study were patients with [...] mini mental state examination score ranged from 19 to 24". Study report does not mention the outcome of interest as a follow up measure and protocol not available for assessment. |
| Yan | Zheng 2017 | Y |  |  | H |  |
| Yang | Pavlovic 2012 | Y | N | NA | C | Outcome data have been reported as "middle values" only with no measure of variability and no justification for non-normality of the data, thus preventing the study to be included in meta-analysis. |
| Yau | Arthur 2000 | Y | N | NA | C | The trial reports a death "of noncardiac causes between the 6- to 8-week postoperative follow-up and the 6-month postoperative follow-up", but the group he had been assigned to was not specified. |
| Yau | Ku 2002 | Y | N | NA | C | The trial reports that "A total of 70 patients were selected [...]. 10 of these patients had previously undergone heart surgery or had experienced severe complications or death during the operation and were therefore excluded", but it is not specified how many patients died and the group they had been assigned to. |
| Yau | Rosenfeldt 2011 | Y | N | NA | F | The trial does not report death among the outcomes, but it is stated that "None [of the 117 patients] were lost to follow-up", so it can be assumed that none of the patients died. |
| Yau | Sawatzky 2014 | Y | N | NA | C | The trial reports that "data on operative and postoperative complications were collected from hospital chart reviews and included 30-day mortality, [...]" in the methods section and that "Neither surgery parameters nor prevalent postoperative complications differed between the two groups (Table 1)" in the results section. Table 1 does not present data on mortality, stating that "Only data on complications that were prevalent are presented". |
| Yau | Stammers 2016 | Y | Y | N | B | This is a master thesis and uses a subset of patients from an RCT, which protocol lists death as an outcome, yet there is no mention of this outcome in the paper. |
| Ye | Belardinelli 2006 | Y | N | NA | D | Study report does not mention the outcome of interest and protocol not available for assessment. |
| Ye | Conraads 2007 | Y | N | NA | D | Study report does not mention the outcome of interest and protocol not available for assessment. |
| Ye | Patwala 2009 | Y | N | NA | D | Study report does not mention the outcome of interest and protocol not available for assessment. |
| Ye | Zeilter 2015 | Y | N | NA | F | The outcome was listed in the trial reports, but there was no event (e.g., the outcome was death but no death occurred). |
| Yeh | Pompa 2016 | Y | N | NA | D | Study report does not mention the outcome of interest and protocol not available for assessment. |
| Yue | Chorna 2014 | Y | Y | Y | A | Study report and protocol do not mention the outcome of interest. |
| Yue | Jabraeili 2016 | Y | N | NA | D | Study report does not mention the outcome of interest and protocol not available for assessment. |
| Zhao_a | Dong 2009 | Y |  |  | H |  |
| Zhao_a | Liu 2014 | Y |  |  | H |  |
| Zhao_a | Ma 2014 | Y |  |  | H |  |
| Zhao_a | Xiao 2019 | Y |  |  | H |  |
| Zhao_b | Gurcay 2008 | Y | N | NA | D | Study report does not mention the outcome of interest and protocol not available for assessment. |
| Zhou | Eyre 2017 | Y | Y | Y | A | The outcome measure was used as an exclusion criterion, as stated in the paper "Exclusion criteria included the following: [...] Mini Mental Health Examination score of 24 or below". Study report and protocol do not mention the outcome of interest as a follow up measure. |
| Zhou | Kang 2014 | Y |  |  | G | Article in Korean. |
| Zhou | Liu-Ambrose 2016 | Y | Y | Y | E | The outcome was measured with a different scale (the Alzheimer disease assessment scale-cognition [ADAS-cog]) and therefore not included in meta-analysis. |
| Zhou | Sungkarat 2017 | Y | N | NA | D | The outcome was measured at baseline. Study report does not mention the outcome of interest as a follow up measure and protocol not available for assessment. |
| Zhou | Sungkarat 2018 | Y | Y | Y | A | The outcome was measured at baseline. No reference to the outcome measure in the trial nor in the protocol as a follow up measure. |
| Zhu | Galantino 2004 | Y | N | NA | D | Study report does not mention the outcome of interest and protocol not available for assessment. |
| Zhu | Jacobs 2004 | Y | N | NA | B | Study methods report that "The outcomes were assessed at baseline and at 1, 3, and 6 months follow-up", but there were no data in the results section. |
| Zhu | Saper 2017 | Y | Y | Y | C | Study protocol reporting the outcome to be measured at 6 weeks, but study report reporting the outcome as mean and SD at 12 weeks only and other timepoints graphically only. |
| Zhu | Teut 2016 | Y | Y | Y | E | Intervention duration exceeding the 4 to 8 weeks timepoint of assessment and the first assessment was at 12 weeks. |
| Zhu | Tilbrook 2011 | Y | Y | Y | E | Intervention duration exceeding the 4 to 8 weeks timepoint of assessment and the first assessment was at 12 weeks. |
| Zhu | Williams 2005 | Y | N | NA | E | Intervention duration exceeding the 4 to 8 weeks timepoint of assessment and the first assessment was at 16 weeks. |
| Zhu | Williams 2009 | Y | N | NA | E | Intervention duration exceeding the 4 to 8 weeks timepoint of assessment and the first assessment was at 24 weeks. |
| Ziebart | Duff 2010 | Y | Y | Y | F | The outcome was available but not included in the review |
| Ziebart | La Grow 2006 | Y | Y | Y | F | The outcome was available but not included in the review |
| Ziebart | Stevens 2001 | Y | N | NA | E | The trial is a secondary analysis of Stevens 2001 already included in the MA. |

***Legend:*** A= Inadequate planning; B= Selective reporting; C= Incomplete reporting; D= Unable to distinguish between selective reporting and inadequate planning; E= Justified to be not included; F= Other situations; G= Not assessed – Language; H= Not assessed – Not found and not possible to judge.

***Abbreviations:*** N= No; NA= Not able to assess; Y= Yes
