# Supplementary material for: Reasons for missing evidence in rehabilitation meta-analyses: a cross-sectional meta-research study
Source: BMC Med Res Methodol. 2023 Oct 21;23:245. doi: 10.1186/s12874-023-02064-7 (PMC10590516; doi:10.1186/s12874-023-02064-7)
Supplement: Supplementary file 5 — Additional file 5: Supplementary Table 1. Characteristics of included reviews. [file 12874_2023_2064_MOESM5_ESM.docx]

**Supplementary Table 1 – Characteristics of included reviews**

| *Country of the corresponding author* | | N | % |
| --- | --- | --- | --- |
|  | Europe | 51 | 38,9% |
|  | Asia | 33 | 25,2% |
|  | South America | 21 | 16% |
|  | Oceania | 17 | 13% |
|  | North America | 9 | 6,9% |
|  | Africa | 0 | 0% |
| *Studies included in the systematic review* | | |  |
|  | Min | 2 | |
|  | Max | 76 | |
|  | Median | 14 | |
|  | Interquartile range (I quartile - III quartile) | 8 - 22,5 | |
|  | Total | 2399 | |
| *Source of funding* | | **N** | **%** |
|  | No Profit | 68 | 51,9% |
|  | None | 44 | 33,6% |
|  | Not reported | 15 | 11,5% |
|  | Mixed | 3 | 2,3% |
|  | Profit | 1 | 0,8% |
| *Source of the protocol* | | **N** | **%** |
|  | PROSPERO | 106 | 80,9% |
|  | Published protocol | 12 | 9,2% |
|  | Previous version of the revision | 10 | 7,6% |
|  | Other registries | 3 | 2,3% |
| *Consultation of grey literature and unpublished sources* | | **N** | **%** |
|  | Yes | 59 | 45% |
|  | No | 72 | 55% |
| *List of excluded studies with detailed reasons for exclusion for each study* | | **N** | **%** |
|  | Yes | 37 | 28,2% |
|  | No | 94 | 71,8% |
| *Studies excluded because they do not report any outcome of interest* | | **N** | **%** |
|  | Yes | 77 | 58,8% |
|  | No | 45 | 34,4% |
|  | Information not available | 9 | 6,9% |
| *Reporting biases mentioned by the authors* | | **N** | **%** |
|  | Yes | 112 | 85,5% |
|  | No | 19 | 14,5% |
