# Supplementary material for: Reasons for missing evidence in rehabilitation meta-analyses: a cross-sectional meta-research study
Source: BMC Med Res Methodol. 2023 Oct 21;23:245. doi: 10.1186/s12874-023-02064-7 (PMC10590516; doi:10.1186/s12874-023-02064-7)
Supplement: Supplementary file 6 — Additional file 6: Supplementary Table 2. Absolute frequencies, relative frequencies and column percentages obtained by cross-referencing the information on the list of excluded studies with detailed exclusion reasons for each study and the exclusion of studies because they do not report any outcome of interest. [file 12874_2023_2064_MOESM6_ESM.docx]

**Supplementary Table 2 – Absolute frequencies, relative frequencies and column percentages obtained by cross-referencing the information on the list of excluded studies with detailed exclusion reasons for each study and the exclusion of studies because they do not report any outcome of interest.**

|  | | *Studies excluded because they do not report any outcome of interest* | | |  |
| --- | --- | --- | --- | --- | --- |
|  |  | Yes | No | Information not available |  |
| *List of excluded studies with detailed reasons for exclusion for each study* | Yes | 17 (13%) 22,1% | 20 (15,3%) 44,4% | 0 (0%) 0% | 37 (28,2%) |
|  | No | 60 (45,8%) 77,9% | 25 (19,1%) 55,6% | 9 (6,9%) 100% | 94 (71,8%) |
|  | | 77 (58,8%) 100% | 45 (34,4%) 100% | 9 (6,9%) 100% | 131 (100%) |
