# Supplementary material for: Reasons for missing evidence in rehabilitation meta-analyses: a cross-sectional meta-research study
Source: BMC Med Res Methodol. 2023 Oct 21;23:245. doi: 10.1186/s12874-023-02064-7 (PMC10590516; doi:10.1186/s12874-023-02064-7)
Supplement: Supplementary file 7 — Additional file 7: Supplementary Table 3. Characteristics of the index meta–analyses. [file 12874_2023_2064_MOESM7_ESM.docx]

**Supplementary Table 3 – Characteristics of the index meta–analyses**

|  | *Studies included in the IMAs* | | | *Studies omitted from the IMAs and assessed for omission* | | | *Eligible studies for the IMAs* | | | *Omitted studies compared to all eligible studies* | | |
| --- | --- | --- | --- | --- | --- | --- | --- | --- | --- | --- | --- | --- |
|  | *CSRs* | *nCSRs* | *Overall* | *CSRs* | *nCSRs* | *Overall* | *CSRs* | *nCSRs* | *Overall* | *CSRs* | *nCSRs* | *Overall* |
| Min | 2 | 2 | 2 | 0 | 0 | 0 | 2 | 2 | 2 | 0% | 0% | 0% |
| Max | 32 | 41 | 41 | 52 | 31 | 52 | 71 | 43 | 71 | 90,5% | 92,3% | 92,3% |
| Median | 5 | 6 | 6 | 5,5 | 2 | 3 | 11 | 10 | 10 | 53,6% | 25,0% | 31,3% |
| IQR  (IQ – IIIQ) | 2 – 7,5 | 4 – 11 | 3 – 10,5 | 1,25 – 12,5 | 1 – 6 | 1 – 7 | 5,5 – 20,75 | 7–16 | 7 – 17 | 21,3% – 74,6% | 5,3% – 50% | 6,1% – 55,6% |
| Total | 159 | 885 | 1044 | 231 | 486 | 717 | 390 | 1371 | 1761 | 59,2% | 35,4% | 40,7% |

**Legend**: CSR: Cochrane Systematic Review; IMA: Index Meta-Analysis; IQR: interquartile range; IQ: first quartile; IIIQ: third quartile; Max: maximum; Min: minimum; nCSR: non-Cochrane Systematic Review
