# Supplementary material for: Reasons for missing evidence in rehabilitation meta-analyses: a cross-sectional meta-research study
Source: BMC Med Res Methodol. 2023 Oct 21;23:245. doi: 10.1186/s12874-023-02064-7 (PMC10590516; doi:10.1186/s12874-023-02064-7)
Supplement: Supplementary file 8 — Additional file 8: Supplementary Table 4. Information concerning a registered protocol. [file 12874_2023_2064_MOESM8_ESM.docx]

**Supplementary Table 4 – Information concerning a registered protocol**

| *Registered protocol* | Total | | Publication year | | | | |
| --- | --- | --- | --- | --- | --- | --- | --- |
|  |  |  | < 2014 | | ≥ 2014 | | Δ* |
|  | N | % | N | % | N | % | % |
| Yes | 210 | 29,3% | 66 | 18,0% | 144 | 41,1% | 23,1% |
| No | 423 | 59,0% | 260 | 70,8% | 163 | 46,6% | -24,2% |
| Information not available | 84 | 11,7% | 41 | 11,2% | 43 | 12,3% | 1,1% |
| TOTAL | 717 |  | 367 |  | 350 |  |  |

* Δ%= %(≥ 2014) – %(<2014)
