# Supplementary material for: Reasons for missing evidence in rehabilitation meta-analyses: a cross-sectional meta-research study
Source: BMC Med Res Methodol. 2023 Oct 21;23:245. doi: 10.1186/s12874-023-02064-7 (PMC10590516; doi:10.1186/s12874-023-02064-7)
Supplement: Supplementary file 9 — Additional file 9: Supplementary Table 5. Comparison of the proportion of omitted RCTs for each reason for omission according to year of publication. [file 12874_2023_2064_MOESM9_ESM.docx]

**Supplementary Table 5– Comparison of the proportion of omitted RCTs for each reason for omission according to year of publication**

| *Reason for omission* | Publication year | | | |  |
| --- | --- | --- | --- | --- | --- |
|  | <2014 | | ≥ 2014 | | Δ* |
|  | N | % | N | % | % |
| Inadequate planning | 36 | 9,8% | 86 | 24,6% | 14,8% |
| Selective reporting | 13 | 3,5% | 11 | 3,1% | -0,4% |
| Incomplete reporting | 35 | 9,5% | 25 | 7,1% | -2,4% |
| Unable to distinguish between selective reporting and inadequate planning | 161 | 43,9% | 121 | 34,6% | -9,3% |
| Justified to be not included | 59 | 16,1% | 49 | 14,0% | -2,1% |
| Other situations | 22 | 6,0% | 15 | 4,3% | -1,7% |
| Not assessed - Language | 8 | 2,2% | 12 | 3,4% | 1,2% |
| Not assessed - Not found and not possible to judge | 33 | 9,0% | 31 | 8,9% | -0,1% |
| TOTAL | 367 | 100% | 350 | 100% |  |

* Δ%= %(≥ 2014) – %(<2014)
